# Irinotecan hydrochloride liposome HR070803 in combination with 5-fluorouracil and leucovorin in locally advanced or metastatic pancreatic ductal adenocarcinoma following prior gemcitabine-based therapy (PAN-HEROIC-1): a phase 3 trial

**DOI:** 10.1038/s41392-024-01948-4

**Published:** 2024-09-19

**Authors:** Jiujie Cui, Shukui Qin, Yuhong Zhou, Shuang Zhang, Xiaofeng Sun, Mingjun Zhang, Jiuwei Cui, Weijia Fang, Kangsheng Gu, Zhihua Li, Jufeng Wang, Xiaobing Chen, Jun Yao, Jun Zhou, Gang Wang, Yuxian Bai, Juxiang Xiao, Wensheng Qiu, Bangmao Wang, Tao Xia, Chunyue Wang, Li Kong, Jiajun Yin, Tao Zhang, Xionghu Shen, Deliang Fu, Chuntao Gao, Huan Wang, Quanren Wang, Liwei Wang

**Affiliations:** 1 Oncology Department and State Key Laboratory of Systems Medicine for Cancer of Shanghai Cancer Institute, Renji Hospital, School of Medicine, Shanghai Jiaotong University, Shanghai, China; 2 GI Cancer Center, Nanjing Tianyinshan Hospital, China Pharmaceutical University, Nanjing, China; 3 Medical Oncology, Zhongshan Hospital Affiliated with Fudan University, Shanghai, China; 4 Department of Biotherapy, West China School of Medicine/West China Hospital of Sichuan University, Chengdu, China; 5 Internal Medicine, Jiangsu Cancer Hospital & Jiangsu Institute of Cancer Research & The Affiliated Cancer Hospital of Nanjing Medical University, Nanjing, China; 6 Medical Oncology, The Second Affiliated Hospital of Anhui Medical University, Hefei, China; 7 Department of Oncology, The First Hospital of Jilin University, Changchun, China; 8 Medical Oncology III, The First Affiliated Hospital, Zhejiang University School of Medicine, Hangzhou, China; 9 Medical Oncology, The First Affiliated Hospital of Anhui Medical University, Hefei, China; 10 Department of Oncology, Sun Yat-sen Memorial Hospital, Sun Yat-sen University, Guangzhou, China; 11 Department of Oncology, The Affiliated Cancer Hospital, Zhengzhou University, Zhengzhou, China; 12 Department of Oncology, The First Affiliated Hospital of Henan University of Science and Technology, Luoyang, China; 13 Department of Gastrointestinal Oncology, Peking University Cancer Hospital & Institute, Beijing, China; 14 Department of Oncology, The First Affiliated Hospital of USTC Anhui Provincial Hospital, Hefei, China; 15 Department of Oncology, Harbin Medical University Cancer Hospital, Harbin, China; 16 Medical Oncology, The First Affiliated Hospital of Xi’an Jiaotong University, Xi’an, China; 17 Department of Oncology, The Affiliated Hospital of Qingdao University, Qingdao, China; 18 Department of Gastroenterology, Tianjin Medical University General Hospital, Tianjin, China; 19 Division of Gastrointestinal and Pancreatic Surgery, Zhejiang Provincial People’s Hospital, Hangzhou, China; 20 Medical Oncology, The First Affiliated Hospital of Xiamen University, Xiamen, China; 21 Department of Oncology, Shandong Cancer Hospital and Institute, Shandong First Medical University and Shandong Academy of Medical Sciences, Jinan, China; 22 General Surgery, Affiliated Zhongshan Hospital of Dalian University, Dalian, China; 23 Cancer Center, Union Hospital, Tongji Medical College, Huazhong University of Science and Technology, Wuhan, China; 24 Department of Oncology, Affiliated Hospital of Yanbian University, Yanji, China; 25 Pancreatic Surgery, Huashan Hospital, Fudan University, Shanghai, China; 26 Pancreatic Surgery, Tianjin Medical University Cancer Institute & Hospital, Tianjin, China; 27 Jiangsu Hengrui Pharmaceuticals Co., Ltd, Shanghai, China

**Keywords:** Clinical trials, Drug development

## Abstract

Liposomal irinotecan has shown promising antitumor activity in patients with advanced or metastatic pancreatic ductal adenocarcinoma (PDAC) who have undergone prior gemcitabine-based therapies. This randomized, double-blind, parallel-controlled, multicenter phase 3 study (NCT05074589) assessed the efficacy and safety of liposomal irinotecan HR070803 combined with 5-fluorouracil (5-FU) and leucovorin (LV) in this patient population. Patients with unresectable, locally advanced, or metastatic PDAC who had previously received gemcitabine-based therapies were randomized 1:1 to receive either HR070803 (60 mg/m^2^ anhydrous irinotecan hydrochloride, equal to 56.5 mg/m^2^ free base) or placebo, both in combination with 5-FU (2000 mg/m^2^) and LV (200 mg/m^2^), all given intravenously every two weeks. The primary endpoint of the study was overall survival (OS). A total of 298 patients were enrolled and received HR070803 plus 5-FU/LV (HR070803 group, n = 149) or placebo plus 5-FU/LV (placebo group, n = 149). Median OS was significantly improved in the HR070803 group compared to the placebo group (7.4 months [95% CI 6.1–8.4] versus 5.0 months [95% CI 4.3–6.0]; HR 0.63 [95% CI 0.48–0.84]; two-sided p = 0.0019). The most common grade ≥ 3 adverse events in the HR070803 group were increased gamma-glutamyltransferase (19.0% versus 11.6% in placebo group) and decreased neutrophil count (12.9% versus 0 in placebo group). No treatment-related deaths occurred in the HR070803 group, while the placebo group reported one treatment-related death (abdominal infection). HR070803 in combination with 5-FU/LV has shown promising efficacy and manageable safety in advanced or metastatic PDAC in the second-line setting, representing a potential option in this patient population.

## Introduction

Pancreatic cancer remains one of the most lethal malignancies, characterized by a poor prognosis and high mortality rate, representing a significant global health challenge.^1–3^ It ranks as the seventh leading cause of cancer-related death worldwide. In 2020, approximately 495,773 new cases of pancreatic cancer were diagnosed, and 466,003 individuals died due to pancreatic cancer. The insidious nature of this disease often results in late-stage diagnosis, and most patients presenting with advanced or metastatic disease. As a consequence, the 5-year survival rate of pancreatic cancer is dismally low at around 10%, underscoring the urgent need for more effective treatments.^1–4^ The current standard of care for treatment-naive patients with locally advanced or metastatic pancreatic ductal adenocarcinoma (PDAC) includes chemotherapy regimens such as AG (nab-paclitaxel plus gemcitabine) and FOFLIRINOX (a combination of leucovorin [LV], fluorouracil [FU], irinotecan hydrochloride, and oxaliplatin).^5–9^ Despite the initial efficacy of these treatments, the majority of patients eventually experience disease progression or relapse, underscoring the necessity for developing effective second-line treatment strategies for those after front-line therapy.^10^

Liposomal irinotecan represents a significant advancement in pancreatic cancer treatment by leveraging nanotechnology to optimize drug delivery. Liposomal formulations encapsulate the drug in liposomes, enhancing delivery to tumor sites while minimizing systemic exposure and associated toxicities. This encapsulation not only protects the active drug from premature degradation but also facilitates its preferential accumulation in tumor tissues through the enhanced permeability and retention effect.^11–18^ Onivyde, an irinotecan hydrochloride liposome injection, exemplifies the clinical potential of this technology. When used in combination with FU/LV, Onivyde has been demonstrated to improve overall survival (OS) in patients with metastatic PDAC previously treated with gemcitabine-based chemotherapy.^14,15^ Based on the positive outcomes of this NAPOLI-1 study, the U.S. Food and Drug Administration (FDA) granted approval for the use of Onivyde in combination with FU/LV in this population. However, despite its proven efficacy and regulatory approval in the United States, Onivyde has not been universally adopted worldwide for the treatment of metastatic PDAC. This disparity in adoption underscores the need for continuous development of new therapeutic agents that can provide more effective treatment options.^4,19^

HR070803 is a novel irinotecan hydrochloride liposome that has been under development since 2008, building upon the foundation of existing commercial irinotecan liposome formulations. This innovative formulation utilizes advanced nano-liposomal technology, incorporating surface-modified polyethylene glycol (PEG)-phospholipids. These modifications are designed to enhance both drug loading and stability by shielding the liposomes from recognition and uptake by the reticuloendothelial system, which is responsible for clearing foreign particles from the bloodstream. This shielding effect allows the drug to circulate in the body for a longer period, increasing the likelihood of it reaching and penetrating tumor tissues. One of the notable advantages of HR070803 over other irinotecan liposomes, including Onivyde, is its smaller particle size. While Onivyde and similar formulations have particle sizes exceeding 100 nanometers (nM), HR070803 has been engineered to have a particle size of approximately 80–90 nM. This reduction in size is not merely a technical improvement but has significant therapeutic implications. Smaller particles exhibit better permeability and retention within tumor tissues, allowing the drug to accumulate more efficiently at the tumor site, thereby prolonging drug exposure and enhancing its therapeutic impact. The increased retention within tumors facilitates a more targeted delivery to cancer cells, which is crucial for maximizing the efficacy of drug while minimizing its side effects on healthy tissues. Moreover, HR070803 has shown potential in improving the therapeutic index of irinotecan by reducing the maximum plasma concentration (C_max_),^20^ potentially mitigating dose-related toxicities. The unique properties of HR070803, therefore, position it as a promising candidate for enhancing both the efficacy and safety of irinotecan-based cancer therapies. Preclinical studies of HR070803 have demonstrated its potent antitumor efficacy and favorable toxicity profile in nude mouse xenograft models, which were mainly attributed to its extended half-life and reduced C_max_ achieved through encapsulation in nanosized liposomes (data on file, Hengrui). Phase 1 clinical studies further validated the therapeutic potential of HR070803, showing significant antitumor activity with a favorable safety profile, both as a monotherapy and in combination with 5-FU/LV, in patients with advanced or metastatic solid tumors, including PDAC (data on file, Hengrui).^20^ These promising results highlight HR070803 as a viable candidate for enhancing the efficacy and safety of irinotecan-based cancer therapies.

In light of these findings, we conducted this randomized phase 3 PAN-HEROIC-1 study to evaluate the efficacy and safety of HR070803 combined with 5-FU/LV compared to placebo combined with 5-FU/LV in patients with unresectable, locally advanced, or metastatic PDAC who had received gemcitabine-based therapy. Based on the results of the interim analysis of this study, HR070803 in combination with 5-FU/LV received approval from the China National Medical Products Administration for treating locally advanced or metastatic PDAC in the second-line setting in January 2024. Here, we present the results of this interim analysis from the PAN-HEROIC-1 study.

## Results

### Patients

**Fig. 1:**
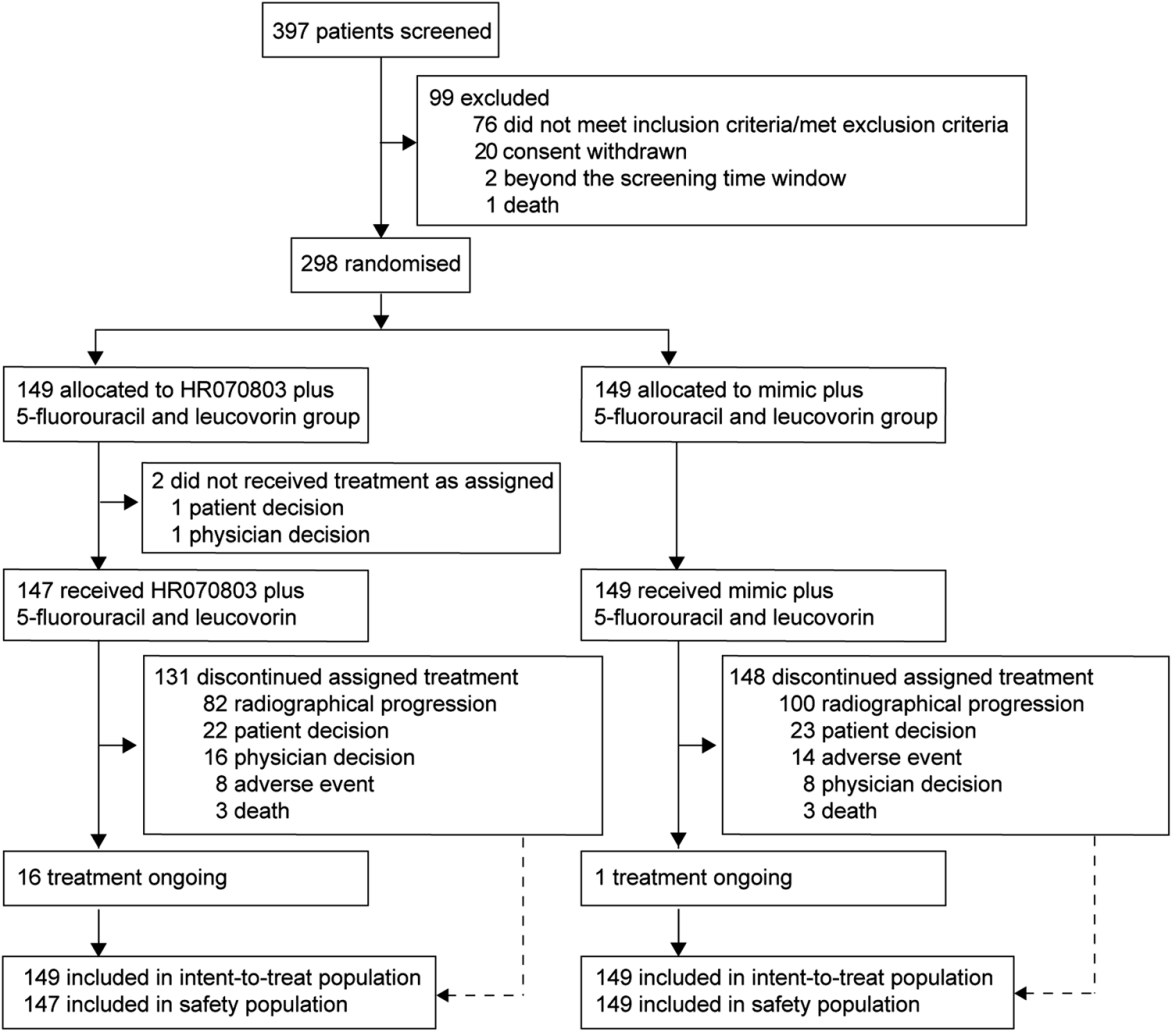
Trial profile

The study enrolled 298 patients between January 25, 2018 and May 26, 2021, with 149 patients randomized to receive HR070803 in combination with 5-FU/LV (HR070803 group) and 149 patients to placebo with 5-FU/LV (placebo group; Fig. 1). In the HR070803 group, two patients did not receive the study drug. Baseline characteristics and previous treatment history were well-balanced across the two study groups (Table 1, Supplementary Table S1). Five patients (3.4%) in the HR070803 group and 6 patients (4.0%) in the placebo group had locally advanced pancreatic cancer, while 144 patients (96.6%) in the HR070803 group and 142 patients (95.3%) in the placebo group were diagnosed with metastatic pancreatic cancer. Prior treatment regimens were similar between the groups: 18.1% versus 14.8% had received prior gemcitabine monotherapy, 93.3% versus 91.9% had gemcitabine combination therapy, 10.1% had FU-based therapy in both groups, 2.0% versus 0.7% had paclitaxel monotherapy, and 48.3% versus 45.6% had undergone pancreatic cancer surgery. As of the interim analysis cutoff date (November 18, 2021), the median duration of follow-up was 12.8 months (IQR 8.9–15.4). Discontinuation of treatment, which was mainly due to disease progression, occurred in 131 patients (87.9%) in HR070803 group and 148 patients (99.3%) in the placebo group. Post-discontinuation therapy was administered to 77 patients (51.7%) in the HR070803 group and 102 patients (68.5%) in the placebo group, with chemotherapy being the most common post-discontinuation treatment (Supplementary Table S2).

**Table 1:** Baseline characteristics

	HR070803 group (*n* = 149)	Placebo group (*n* = 149)
Age, years	60 (52–66)	59 (54–65)
Male	95 (63.8)	93 (62.4)
ECOG performance status		
0	25 (16.8)	26 (17.5)
1	124 (83.2)	123 (82.6)
Pancreatic tumor location		
Head	60 (40.3)	65 (43.6)
Body	6 (4.0)	7 (4.7)
Tail	62 (41.6)	59 (39.6)
Multicentric	21 (14.1)	18 (12.1)
Clinical TNM stage		
Stage II-III (locally advanced)	5 (3.4)	6 (4.0)
Stage IV (metastatic)	144 (96.6)	142 (95.3)
Site of metastatic lesions		
Liver	110 (73.8)	100 (67.1)
Lung	38 (25.5)	40 (26.9)
Lymph node, regional	49 (32.9)	44 (29.5)
Lymph node, distant	42 (28.2)	55 (36.9)
Peritoneum	21 (14.1)	12 (8.1)
Other	54 (36.2)	55 (36.9)
Number of metastatic sites		
0	1 (0.7)	3 (2.0)
1	40 (26.8)	45 (30.2)
2	58 (38.9)	49 (32.9)
3	33 (22.1)	33 (22.1)
≥ 4	17 (11.4)	19 (12.8)
UGT1A1 gene mutation		
UGT1A1*28	4 (2.7)	5 (3.4)
UGT1A1*6^a^	2 (1.3)	3 (2.0)
CA19-9, U/mL	267.3 (64.5–1523.5)	503.8 (68.6–2056.0)
Albumin		
< 40 g/L	45 (30.2)	46(30.9)
≥ 40 g/L	104 (69.8)	103(69.1)
Previous antitumor therapy		
Gemcitabine monotherapy	27 (18.1)	22 (14.8)
Gemcitabine combination	139 (93.3)	137 (91.9)
Fluorouracil-based	15 (10.1)	15 (10.1)
Paclitaxel monotherapy	3 (2.0)	1 (0.7)
Surgery for pancreatic cancer	72 (48.3)	68 (45.6)
Duration since the diagnosis of the disease, months	6.7 (4.5–9.9)	6.9 (3.7–9.7)

Data are median (IQR) or *n* (%)

*ECOG* Eastern Cooperative Oncology Group, *IQR* Interquartile range

^a^Data were missing for one patient in the placebo group

### Efficacy

**Fig. 2:**
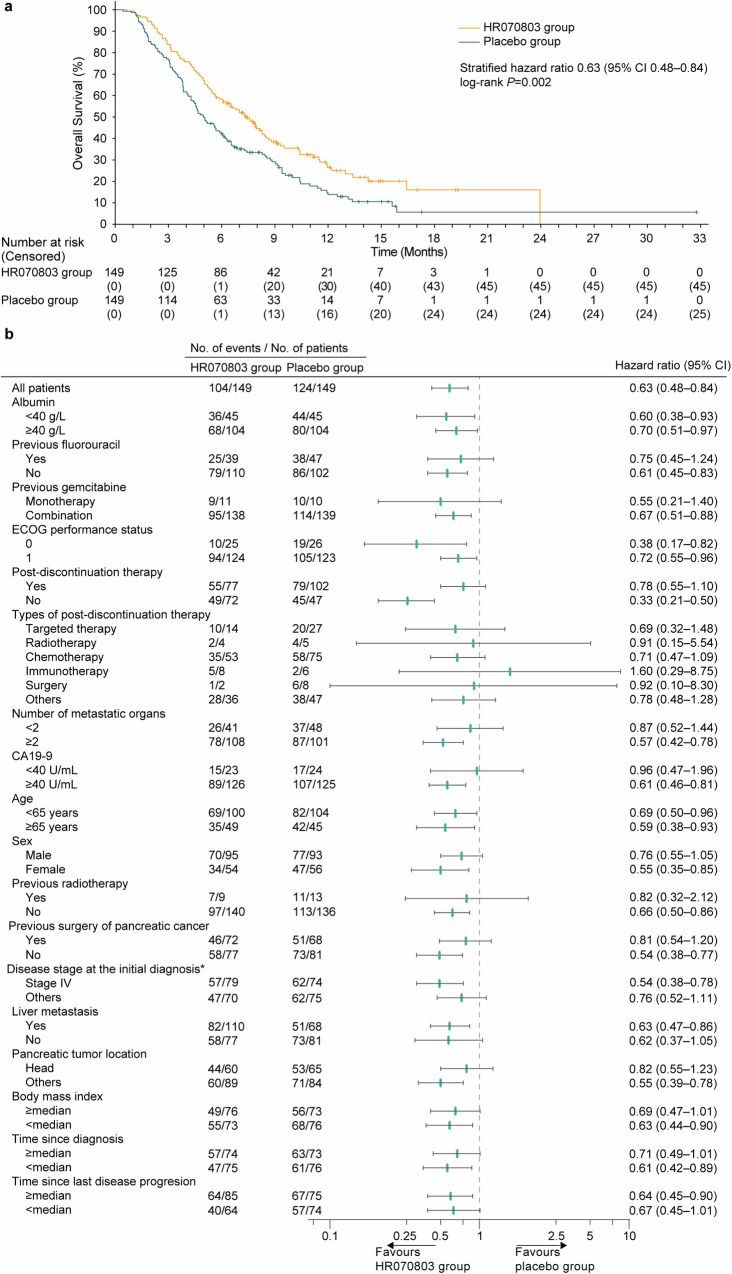
OS. (**a**) Kaplan-Meier plot of OS. (**b**) Subgroup analysis. *It indicates the number of patients who were diagnosed with stage IV at the initial pancreatic cancer diagnosis, not during the enrollment period of this study

As of the data cutoff for the interim analysis, 104 of the 149 patients (69.8%) in the HR070803 group and 124 of the 149 patients (83.2%) in the placebo group had died. The combination of HR070803 with 5-FU/LV significantly improved OS compared to the placebo plus 5-FU/LV (median, 7.4 months [95% CI 6.1–8.4] versus 5.0 months [95% CI 4.3–6.0]; hazard ratio [HR], 0.63 [95% CI 0.48–0.84]; one-sided *P* = 0.002; Fig. 2a). Subgroup analyzes indicated that OS benefits favored the HR070803 group versus placebo group across all predefined subgroups (Fig. 2b).

**Fig. 3:**
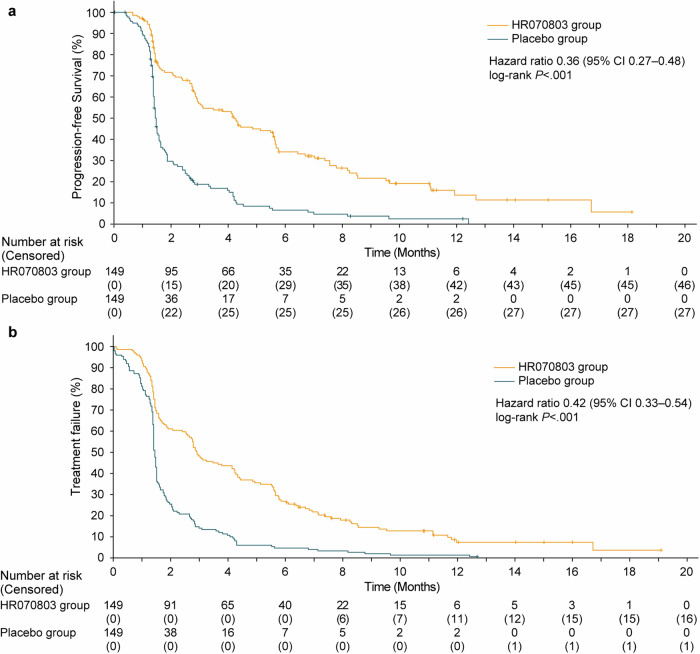
Kaplan-Meier plots of PFS and TTF (**a**) PFS. (**b**) TTF

Progression-free survival (PFS) also showed improvement with HR070803 plus 5-FU/LV compared to the placebo plus 5-FU/LV (median, 4.2 months [95% CI 2.9–5.6] versus 1.5 months [95% CI 1.4–1.6]; HR, 0.36 [95% CI 0.27–0.48]; two-sided *P* < 0.001; Fig. 3a). The median time to treatment failure (TTF) was 2.9 months (95% CI 2.6–4.2) in the HR070803 group and 1.5 months (95% CI 1.4–1.5) in the placebo group (HR, 0.42 [95% CI 0.33–0.54]; Fig. 3b).

The objective response rate (ORR) was 12.8% (19/149; 95% CI 7.9–19.2) in the HR070803 group and 0.7% (1/149; 95% CI 0–3.7) in the placebo group; all were partial responses (PR) (Supplementary Table S3). The CA19-9 responses were achieved in 41 (31.5%, 95% CI 23.7–40.3) out of 130 evaluable patients from HR070803 group and 5 (3.8%, 95% CI 1.2–8.6) out of 132 evaluable patients from placebo group.

### Safety

The relative dose intensity of HR070803 and placebo was 90.5% ± 10.7 (mean ± SD) and 96.4% ± 8.2, respectively. Additionally, the relative dose intensity of leucovorin was 92.3% ± 9.0 and 96.2% ± 7.5 in the HR070803 and placebo group, respectively. The relative dose intensity of 5-FU was 90.3% ± 11.1 and 95.5% ± 9.0 in each group, respectively.

Adverse events (AEs) were similar in both the HR070803 and placebo groups, with 99.3% (146/147) and 97.3% (145/149) of patients in each group, respectively (Table 2). In the HR070803 group, the most frequent AEs were nausea (61.9%, 91/147]), vomiting (57.8%, [85/147]), and asthenia (53.1%, [78/147]); while the placebo group reported asthenia (42.3%, [63/149]), nausea (40.3%, [60/149]), and anemia (34.9%, [52/149]) as the most common AEs.

**Table 2:** Adverse event

	HR070803 group (*n* = 147)		Placebo group (*n* = 149)	
	Any grade	Grade 3–5	Any grade	Grade 3–5
Any	146 (99.3)	78 (53.1)	145 (97.3)	69 (46.3)
Nausea	91 (61.9)	2 (1.4)	60 (40.3)	0
Vomiting	85 (57.8)	7 (4.8)	48 (32.2)	3 (2.0)
Asthenia	78 (53.1)	6 (4.1)	63 (42.3)	3 (2.0)
Decreased appetite	67 (45.6)	4 (2.7)	50 (33.6)	2 (1.3)
Diarrhea	67 (45.6)	6 (4.1)	36 (24.2)	4 (2.7)
Anemia	59 (40.1)	9 (6.1)	52 (34.9)	4 (2.7)
Neutrophil count decreased	49 (33.3)	19 (12.9)	12 (8.1)	0
Weight decreased	48 (32.7)	1 (0.7)	36 (24.2)	1 (0.7)
White blood cell count decreased	46 (31.3)	12 (8.2)	15 (10.1)	1 (0.7)
Alanine aminotransferase increased	41 (27.9)	6 (4.1)	27 (18.1)	3 (2.0)
Aspartate aminotransferase increased	40 (27.2)	2 (1.4)	32 (21.5)	2 (1.3)
Gamma-glutamyltransferase increased	38 (25.9)	28 (19.1)	33 (22.2)	17 (11.4)
Constipation	33 (22.5)	0	46 (30.9)	1 (0.7)
Hypoalbuminemia	31 (21.1)	0	34 (22.8)	2 (1.3)
Abdominal pain	28 (19.1)	6 (4.1)	32 (21.5)	5 (3.4)
Platelet count decreased	25 (17.0)	0	21 (14.1)	0
Pyrexia	24 (16.3)	0	16 (10.7)	1 (0.7)
Hypokalemia	23 (15.7)	7 (4.8)	19 (12.8)	4 (2.7)
Back pain	21 (14.3)	0	25 (16.8)	4 (2.7)
Abdominal pain upper	21 (14.3)	1 (0.7)	17 (11.4)	1 (0.7)
Blood bilirubin increased	20 (13.6)	11 (7.5)	23 (15.4)	8 (5.4)
Blood alkaline phosphatase increased	18 (12.2)	6 (4.1)	29 (19.5)	5 (3.4)
Dizziness	17 (11.6)	0	20 (13.4)	0
Insomnia	15 (10.2)	0	11 (7.4)	0
Oedema peripheral	15 (10.2)	0	4 (2.7)	0
Abdominal distension	14 (9.5)	1 (0.7)	25 (16.8)	0
Hyponatremia	13 (8.8)	4 (2.7)	16 (10.7)	4 (2.7)
Lymphocyte count decreased	10 (6.8)	2 (1.4)	15 (10.1)	2 (1.3)

Data are *n* (%). Events of any grade occurring in 10% or more patients in either group are listed

Grade 3 or higher AEs occurred in 53.1% (78/147) of patients in the HR070803 group and 46.3% (69/149) in the placebo group. The most frequent grade ≥3 AEs were increased gamma-glutamyltransferase (19.0%, [28/147]) and decreased neutrophil count (12.9%, [19/147]) in the HR070803 group, and increased gamma-glutamyltransferase (11.6%, [17/149]), increased blood bilirubin, and increased bilirubin conjugated (5.4%, [8/149] for each) in the placebo group. Serious AEs occurred in 24.5% (36/147) of the HR070803 group and 17.5% (26/149) of the placebo group (Supplementary Table S4), and treatment-related serious AEs occurred in 12.2% (18/147) of the HR070803 group and 4.0% (6/149) of the placebo group. Dose reduction due to AEs were reported in 21.1% (31/147) and 5.4% (8/149) of patients in each group, respectively. Treatment interruption due to AEs was reported in 31.3% (46/147) of patients in HR070803 group compared to 18.1% (27/149) of patients in placebo group. The incidence of treatment discontinuation due to AEs was 4.1% (6/147) in the HR070803 group and 9.4% (14/149) in the placebo group (Supplementary Table S5). Deaths due to AEs occurred in 2.7% (4/147) of the HR070803 group and 6.0% (9/149) of the placebo group, with one death in the placebo group due to abdominal infection considered treatment-related (Supplementary Table S6).

### Quality of life

All the 298 patients were included in the QLQ-C30 questionnaire analysis. Baseline quality of life measurements, evaluated using the QLQ-C30 scales and single items, showed no significant differences between the HR070803 and placebo groups. At 6 and 12 weeks, mean scores on the QLQ-C30 scales and single items showed no clinically appreciable differences from baseline, indicating minimal impact of the treatments on functional scale scores (Supplementary Fig. S1).

## Discussion

Results of this study demonstrated that HR070803 combined with 5-FU and leucovorin significantly extended OS among patients with unresectable locally advanced, or metastatic PDAC who have failed gemcitabine-based therapy, as compared with placebo plus 5-FU and leucovorin. The HR070803 group exhibited a 37% reduction in the risk of death and a median OS extension of 2.4 months relative to the placebo group. Moreover, patients assigned to HR070803 group showed numerically superior PFS, ORR, TTF, and CA19-9 response. The safety profile of HR070803 plus 5-FU and leucovorin was manageable.

Both this study and the NAPOLI-1 study recruited patients with metastatic PDAC who were previously treated with gemcitabine-based therapies.^14,15^ In the NAPOLI-1 study, treatment with Onivyde (nanoliposomal irinotecan [70 mg/m^2^] combined with FU and folic acid) demonstrated advantages in OS (median 6.2 versus 4.2 months, HR 0.63), PFS (median 3.1 versus 1.5 months, HR 0.56), and TTF (2.3 versus 1.4 months, HR 0.6) compared to FU plus folic acid group, and thus became the standard of care in this patient population.^14,15^ Our study indicated that the efficacy of HR070803 combined with 5-FU and leucovorin was comparable to this standard therapy, despite a lower dose of liposomal irinotecan (56.5 mg/m^2^). The promising efficacy of HR070803 may partially be attributed to the long median drug exposure time (17.4 weeks), as well as the small liposome particle size (approximately 80–90 nM), which could improve penetration of the drug to the target tumor lesion and thus contribute to a favorable efficacy profile. While cross-trial comparisons should be interpreted with caution, our results suggest that HR070803 plus 5-FU and leucovorin might be a promising alternative in regions where Onivyde is not readily available.

AEs that occurring more frequently (≥ 10%) in the HR070803 group compared to the placebo group included nausea, vomiting, diarrhea, decreased white blood cell count, decreased neutrophil count, loss of appetite, and fatigue. These AEs were consistent with those known for irinotecan, and no new toxicities were observed. The slow-release nature of HR070803 liposome maintains the blood concentrations of the active metabolite SN-38 and total irinotecan at stable lower levels, resulting in relative lower incidences of decreased neutrophil count, diarrhea, and cholinergic syndrome compared to that of irinotecan hydrochloride (54%–96.9%, 72.4%–88%, and 28.3%, respectively).^21–23^

The AE profile in our trial aligned with previous reports for irinotecan liposome.^14–18^ In our study, neither treatment discontinuation nor serious events resulting from neutropenia and diarrhea were observed. The incidence of diarrhea and decreased neutrophil count with HR070803 combination treatment was 45.6% (grade ≥ 3: 4.1%) and 33.3% (grade ≥ 3: 12.9%), respectively, compared to 59% (grade ≥ 3: 13%) and 39% (grade ≥ 3: 27%) in NAPOLI-1.^14,15^ Additionally, the proportion of patients who required drug discontinuation and dose reduction due to AEs in our study was 4.1% and 21.1%, respectively, while in the NAPOLI-1 study, it was 11% and 33%, respectively.^14,15^ The low incidence of toxicity observed suggests that HR070803 may offer an extended therapeutic window, as evidenced by a median drug exposure duration of 17.4 weeks in our study and 8.7 weeks in the NAPOLI-1 trial. This extension of the therapeutic window could potentially enhance the antitumor efficacy of HR070803-based therapy.

UGT1A1 is a crucial enzyme in irinotecan metabolism. Gene mutations in the UGT1A1 gene and decreased enzyme activity can lead to an increased incidence of diarrhea and decreased neutrophil count caused by irinotecan.^24–26^ In our study, six patients in the HR070803 group had UGT1A1 homozygous mutation, with four having UGT1A1*28 mutation and two having UGT1A1*6 mutation. Among them, only one patient with UGT1A1*28 mutation experienced neutropenia event (grade 3), indicating that the UGT1A1 polymorphism had a limit impact on neutropenia incidence in our study.

Despite the increased incidence of certain AEs associated with HR070803, there was no significant difference in quality of life between the HR070803 and placebo groups. This further suggested that the AEs related to HR070803 combination therapy are acceptable and manageable.

We selected irinotecan placebo for the control group to avoid the possibility of unblinding due to inconsistent appearance between the control drug and the HR070803, and therefore to achieve a double-blind design, and to minimize bias to the greatest extent.

One of the limitations in this study is the small number of patients with UGT1A1 mutations, which did not allow for the exploration of the necessity of dose adjustment of irinotecan hydrochloride liposome in this population. Another limitation of this study is that the current dosing regimen of 2000 mg/m^2^ intravenously over 46 h every two weeks for 5-FU may result in less potent outcomes when extrapolating the efficacy of our HR070803 combination therapy to a global population. This is because the standard of care (Onivyde/FU/leucovorin combination therapy), approved based on the NAPOLI-1 study, uses a dose of 2400 mg/m^2^ of FU under the same administration schedule, although a different dose of FU was administered in the control arm in the NAPOLI-1 study. In our study, the dose and schedule of 5-FU in both arms (same dose and schedule to facilitate a double-blind design, enhance patient compliance, and strengthen the rigor of the study design) were chosen based on clinical practice in Chinese pancreatic cancer patients, who generally have poor tolerance, to improve patient tolerance while ensuring efficacy. Additionally, 5-FU is a time-dependent chemotherapy drug and was administered by continuous infusion over 46 h every two weeks in this study, so we speculate that the efficacy will not be compromised and the toxicities will be more tolerable by reducing the dose of 5-FU to 2000 mg/m^2^. Due to the lack of a direct comparison between our study and NAPOLI-1, the use of this HR070803 combination therapy in racially diverse populations remains to be further clarified.

In conclusion, HR070803 plus 5-FU and leucovorin significantly extended OS and improved other efficacy endpoints in unresectable, locally advanced, or metastatic PDAC who have received gemcitabine-based therapy, compared to placebo plus 5-FU and leucovorin. The safety was acceptable and manageable. This HR070803 combination therapy might represent a novel standard second-line treatment option for this patient population.

## Methods

### Study design and patients

This randomized, double-blind, parallel-controlled, multicenter phase 3 trial was conducted at 54 sites across China.

Eligible patients were adults (≥ 18 years) with histologically confirmed PDAC; had unresectable, locally advanced, or metastatic disease; and had progressed on or shown intolerance to prior first-line gemcitabine-based chemotherapy in locally advanced or metastatic settings. Additional eligibility criteria included having ≥ 1 measurable lesion per Response Evaluation Criteria In Solid Tumors (RECIST) version 1.1, an Eastern Cooperative Oncology Group performance status of 0 or 1, a life expectancy of ≥ 12 weeks, and adequate organ function. Key exclusion criteria were active central nervous system metastases, other malignant tumors within five years; active hepatitis B viral infection; ascites requiring clinical intervention; numerical rating scale pain score ≥ 4 after standardized treatment with analgesic drugs; severe gastrointestinal diseases; uncontrolled cardiovascular and cerebrovascular diseases; and known allergies to any component of irinotecan liposomes or other liposomes, 5-FU, or calcium folinate.

The study adhered to the Declaration of Helsinki and Good Clinical Practice Guidelines. Ethics committee approval was obtained from each study site, and all participants provided written informed consent (Supplementary information).

### Randomization and masking

Patients were randomized 1:1 to receive either HR070803 plus 5-FU/LV or placebo (irinotecan hydrochloride liposome mimic) plus 5-FU/LV. Randomization was conducted using a centralized interactive web-response system, stratified by albumin levels (≥ 40 g/L versus <40 g/L), history of FU-based therapy (used versus never used), and history of gemcitabine-based therapy (gemcitabine monotherapy versus gemcitabine combination therapy). A third party generated the randomization sequence using central block randomization with a block size of 4 per stratum. Blinding was maintained for patients, investigators, and the sponsor’s study team until the interim analysis database lock.

### Procedures

Patients were intravenously infused with HR070803 (60 mg/m^2^ over 90 min, equivalent to 56.5 mg/m^2^ of the irinotecan free-base) combined with 5-FU (2000 mg/m^2^ for 46 ± 4 h) and LV (200 mg/m^2^ for 30 ± 10 min) or placebo combined with 5-FU/LV every two weeks (one cycle) in the order of HR070803 or placebo, leucovorin, and 5-FU. All patients underwent uridine diphosphate glucuronosyltransferase 1A1 (UGT1A1) genotype testing before starting treatment; those homozygous for UGT1A1*28 or *6 began with a reduced HR070803 dose (50 mg/m²) in the first cycle, increasing to the standard dose if no drug-related toxicity occurred. The placebo (irinotecan hydrochloride liposome mimic) was provided by the sponsor and was consistent with HR070803 in appearance.

All patients were given dexamethasone and antiemetics before study drug medication for the prevention of vomiting. Patients received the study drug until disease progression or intolerable toxicity. Dosage adjustment due to toxicity is allowed up to 21 days during the treatment. When the administration of HR070803 needs to be delayed, the administration of 5-FU/LV would be delayed accordingly, and the two agents could not be administered alone.

Tumor response was evaluated every six weeks via CT or MRI per RECIST version 1.1, with complete response (CR) and PR requiring confirmation by subsequent imaging. Carbohydrate antigen 19-9 (CA19-9) was evaluated every six weeks. Survival was tracked monthly until death, and AEs were monitored for 30 days post-treatment, graded according to the National Cancer Institute Common Terminology Criteria for Adverse Events, version 4.03. Quality of life was assessed every 3 cycles throughout the study according to the EORTC QLQ-C30, version 3.0.

### Outcomes

The primary endpoint was OS, defined as the interval from randomization to death from any cause. Secondary endpoints included PFS (the interval from randomization to either disease progression or death, whichever occurred first), TTF (the interval from randomization to disease progression, study termination due to toxicity, or death whichever occurred first), ORR (percentage of patients whose best overall response was CR or PR), CA19-9 response (≥ 50% decrease in CA19-9 levels from baseline during treatment), quality of life, and safety.

### Statistical analysis

Based on an assumption of median OS of 3.5 months for placebo plus 5-FU and leucovorin and 5.0 months for HR070803 plus 5-FU/LV, it was calculated that 253 death events would give the trial with an 80% power to detect the significance in OS between the HR070803 plus 5-FU/LV group and placebo plus 5-FU/LV group, with a one-sided α level of 0.025. The prespecified enrollment duration was 24 months. With an estimated dropout rate of 20%, the target enrollment was set at 272 patients.

Efficacy was assessed in the intent-to-treat set, which included all randomized patients, while safety was assessed in the safety set, consisting of patients who received ≥1 dose of the study medication. CA19-9 response was assessed in patients with baseline CA19-9 level > 30 U/mL, and quality of life outcomes were analyzed for all randomized patients. The Kaplan-Meier method was used to estimate median OS, PFS, and TTF, with 95% CIs calculated using the Brookmeyer-Crowley method. Differences in OS, PFS, and time to treatment failure between groups were evaluated using a stratified log-rank test. Hazard ratios (HRs) with two-sided 95% CIs were determined using stratified Cox proportional-hazards models. ORR and disease control rate were presented with 95% CIs which was calculated using the exact probability-based method.

An interim analysis was prespecified after 70% (177 events) of the expected OS events had taken place. By July 9, 2021, the safety committee identified 188 death events during the periodic review, accounting for 74.3% of the expected number. The safety committee reviewed the data, reporting that the efficacy boundary for OS in the interim analysis had been crossed and the toxicities were acceptable. At that time, the data were considered sufficient for a new drug registration application submission to the National Medical Products Administration (the Chinese counterpart of U.S. FDA). Following the pre-submission to the National Medical Products Administration and the subsequent adjudication process, a total of 228 death events had occurred by November 18, 2021. This data cutoff date was used for the interim analysis and the formal submission of the new drug registration application to the National Medical Products Administration. The pre-specified two-sided significance threshold was 0.036 for the interim analysis, according to the Lan-DeMets (O’Brien-Fleming) α spending function and based on the observed 228 events. The results of the pre-specified interim analysis are reported here. All analyzes were conducted using SAS 9.4 (SAS Institute, Inc, Cary, North Carolina).

## Supplementary information

Supplementary information_Supplementary Tables and Figures

Supplementary information_Protocol and Statistical analysis plan

CONSORT Checklist

COI_disclosure

## Data Availability

Dataset of this study could be obtained from corresponding author with a reasonable request.
