# Supplementary material for: Irinotecan hydrochloride liposome HR070803 in combination with 5-fluorouracil and leucovorin in locally advanced or metastatic pancreatic ductal adenocarcinoma following prior gemcitabine-based therapy (PAN-HEROIC-1): a phase 3 trial
Source: Signal Transduct Target Ther. 2024 Sep 19;9:248. doi: 10.1038/s41392-024-01948-4 (PMC11412970; doi:10.1038/s41392-024-01948-4)
Supplement: Supplementary file 1 — Supplementary information_Supplementary Tables and Figures [file 41392_2024_1948_MOESM1_ESM.docx]

**Supplementary Materials for**

**Irinotecan hydrochloride liposome HR070803 in combination with 5-fluorouracil and leucovorin in locally advanced or metastatic pancreatic ductal adenocarcinoma following prior gemcitabine-based therapy (PAN-HEROIC-1): a phase 3 trial**

Shukui Qin, Jiujie Cui, Yuhong Zhou, Shuang Zhang, Xiaofeng Sun, Mingjun Zhang, Jiuwei Cui, Weijia Fang, Kangsheng Gu, Zhihua Li, Jufeng Wang, Xiaobing Chen, Jun Yao, Jun Zhou, Gang Wang, Yuxian Bai, Juxiang Xiao, Wensheng Qiu, Bangmao Wang, Tao Xia, Chunyue Wang, Li Kong, Jiajun Yin, Tao Zhang, Xionghu Shen, Deliang Fu, Chuntao Gao, Huan Wang, Quanren Wang, Liwei Wang

Correspondence to: Liwei Wang, PhD, MD. Oncology Department and State Key Laboratory of Systems Medicine for Cancer of Shanghai Cancer Institute, Renji Hospital, School of Medicine, Shanghai Jiaotong University, 160 Pujian Road, Shanghai, 200127 China. Phone: +86 13761254228. E-mail: liweiwang@shsmu.edu.cn

**This PDF file includes:**

Tables S1 to S6

Fig. S1

List of participating sites

**Table of contents**

[Table S1. Summary of first-line therapy 2](#_Toc172901356)

[Table S2. Summary of post-discontinuation therapy 3](#_Toc172901357)

[Table S3. Tumor responses 4](#_Toc172901358)

[Table S4. Serious adverse events 5](#_Toc172901359)

[Table S5. Adverse events which led to treatment discontinuation 7](#_Toc172901360)

[Table S6. Adverse events which led to death 8](#_Toc172901361)

[Fig. S1. Changes from baseline in quality of life assessed using the EORTC QLQ-C30 questionnaire 9](#_Toc172901362)

[List of participating sites 10](#_Toc172901363)

## Table S1. Summary of first-line therapy

|  | **HR070803 group (n=149)** | **Placebo group (n=149)** |
| --- | --- | --- |
| Chemotherapy | 149 (100) | 149 (100) |
| Gemcitabine monotherapy | 23 (15.4) | 21 (14.1) |
| Gemcitabine combination | 138 (92.6) | 137 (91.9) |
| Fluorouracil-based | 13 (8.7) | 8 (5.4) |
| Paclitaxel monotherapy | 3 (2.0) | 1 (0.7) |
| Targeted therapy | 9 (6.0) | 5 (3.4) |
| Immunotherapy | 11 (7.4) | 7 (4.7) |

Data are n (%). The percentages of these treatments add up to more than 100% because some patients received treatment regimens that included multiple drugs ang were listed in multiple groups. All patients had received at least one treatment of gemcitabine-based therapy.

## Table S2. Summary of post-discontinuation therapy

|  | **HR070803 group (n=149)** | **Placebo group (n=149)** |
| --- | --- | --- |
| Total | 77 (51.7) | 102 (68.5) |
| Chemotherapy | 53 (35.6) | 75 (50.3) |
| Targeted therapy | 14 (9.4) | 27 (18.1) |
| Immunotherapy | 8 (5.4) | 6 (4.0) |
| Surgery | 2 (1.3) | 8 (5.4) |
| Radiotherapy | 4 (2.7) | 5 (3.4) |
| Others | 36 (24.2) | 47 (31.5) |

Data are n (%).

## Table S3. Tumor responses

|  | **HR070803 group (n=149)** | **Placebo group (n=149)** |
| --- | --- | --- |
| Best overall response |  |  |
| Complete response | 0 | 0 |
| Partial response | 19 (12.8) | 1 (0.7) |
| Stable disease | 77 (51.7) | 39 (26.2) |
| Progressive disease | 35 (23.5) | 80 (53.7) |
| Not evaluable | 15 (10.1) | 25 (16.8) |
| Objective response | 19 (12.8, 7.9–19.2) | 1 (0.7, 0–3.7) |
| Disease control | 96 (64.4, 56.2–72.1) | 40 (26.8, 19.9–34.7) |

Data are n (%) or n (%, 95% CI).

## Table S4. Serious adverse events

|  | **HR070803 group (n=147)** | **Placebo group (n=149)** |
| --- | --- | --- |
| Any | 36 (24.5) | 26 (17.5) |
| Disease progression | 4 (2.7) | 0 |
| Diarrhea | 3 (2.0) | 1 (0.7) |
| Pyrexia | 2 (1.4) | 2 (1.3) |
| Jaundice cholestatic | 2 (1.4) | 2 (1.3) |
| Hepatic function abnormal | 2 (1.4) | 1 (0.7) |
| Abdominal pain | 2 (1.4) | 1 (0.7) |
| Upper gastrointestinal hemorrhage | 2 (1.4) | 0 |
| White blood cell count decreased | 2 (1.4) | 0 |
| Alanine aminotransferase increased | 2 (1.4) | 0 |
| Blood bilirubin increased | 2 (1.4) | 0 |
| Hypokalemia | 2 (1.4) | 0 |
| Sepsis | 2 (1.4) | 0 |
| Anemia | 2 (1.4) | 0 |
| Hypoalbuminemia | 1 (0.7) | 1 (0.7) |
| Nausea | 1 (0.7) | 0 |
| Abdominal pain upper | 1 (0.7) | 0 |
| Impaired gastric emptying | 1 (0.7) | 0 |
| Small intestinal obstruction | 1 (0.7) | 0 |
| Asthenia | 1 (0.7) | 0 |
| Chest discomfort | 1 (0.7) | 0 |
| Bile duct stenosis | 1 (0.7) | 0 |
| Biliary obstruction | 1 (0.7) | 0 |
| Hepatic failure | 1 (0.7) | 0 |
| Gamma-glutamyltransferase increased | 1 (0.7) | 0 |
| Aspartate aminotransferase increased | 1 (0.7) | 0 |
| Hypoglycemia | 1 (0.7) | 0 |
| Hyperglycemia | 1 (0.7) | 0 |
| Biliary tract infection | 1 (0.7) | 0 |
| Myelosuppression | 1 (0.7) | 0 |
| Hypertensive encephalopathy | 1 (0.7) | 0 |
| Lacunar infarction | 1 (0.7) | 0 |
| Hypotension | 1 (0.7) | 0 |
| Hypertension | 1 (0.7) | 0 |
| Tumor hemorrhage | 1 (0.7) | 0 |
| Fracture | 1 (0.7) | 0 |
| Acute kidney injury | 1 (0.7) | 0 |
| Device occlusion | 1 (0.7) | 0 |
| Suicide attempt | 1 (0.7) | 0 |
| Intestinal obstruction | 0 | 3 (2.0) |
| Gastrointestinal hemorrhage | 0 | 2 (1.3) |
| Death | 0 | 2 (1.3) |
| Neoplasm progression | 0 | 2 (1.3) |
| Constipation | 0 | 1 (0.7) |
| Ascites | 0 | 1 (0.7) |
| Ileus paralytic | 0 | 1 (0.7) |
| Obstruction gastric | 0 | 1 (0.7) |
| Cholangitis acute | 0 | 1 (0.7) |
| Abdominal infection | 0 | 1 (0.7) |
| Septic shock | 0 | 1 (0.7) |
| Cerebral infarction | 0 | 1 (0.7) |
| Hypovolemic shock | 0 | 1 (0.7) |
| Fall | 0 | 1 (0.7) |
| Ureterolithiasis | 0 | 1 (0.7) |
| Respiratory failure | 0 | 1 (0.7) |
| Pleural effusion | 0 | 1 (0.7) |
| Anaphylactic shock | 0 | 1 (0.7) |

Data are n (%).

## Table S5. Adverse events which led to treatment discontinuation

|  | **HR070803 group (n=147)** | **Placebo group (n=149)** |
| --- | --- | --- |
| Any | 6 (4.1) | 14 (9.4) |
| Jaundice cholestatic | 1 (0.7) | 2 (1.3) |
| Hepatic function abnormal | 1 (0.7) | 0 |
| Diarrhea | 1 (0.7) | 0 |
| Upper gastrointestinal hemorrhage | 1 (0.7) | 0 |
| Tumor hemorrhage | 1 (0.7) | 0 |
| Myocardial infarction | 1 (0.7) | 0 |
| Infusion related reaction | 0 | 3 (2.0) |
| Hypersensitivity | 0 | 2 (1.3) |
| Anaphylactic shock | 0 | 1 (0.7) |
| Abdominal infection | 0 | 1 (0.7) |
| Septic shock | 0 | 1 (0.7) |
| Gamma-glutamyltransferase increased | 0 | 1 (0.7) |
| Cerebral infarction | 0 | 1 (0.7) |
| Pulmonary embolism | 0 | 1 (0.7) |
| Hypovolemic shock | 0 | 1 (0.7) |

Data are n (%).

## Table S6. Adverse events which led to death

|  | **HR070803 group (n=147)** | **Placebo group (n=149)** |
| --- | --- | --- |
| Any | 4 (2.7) | 9 (6.0) |
| Disease progression | 4 (2.7) | 0 |
| Death | 0 | 2 (1.3) |
| Neoplasm progression | 0 | 2 (1.3) |
| Abdominal infection | 0 | 1 (0.7) |
| Septic shock | 0 | 1 (0.7) |
| Fall | 0 | 1 (0.7) |
| Respiratory failure | 0 | 1 (0.7) |
| Gastrointestinal hemorrhage | 0 | 1 (0.7) |

Data are n (%).

## Fig. S1. Changes from baseline in quality of life assessed using the EORTC QLQ-C30 questionnaire

(**a**) Global health status and function scales on day 15 of cycle 3. (**b**) Symptom and single-item scales on day 15 of cycle 3. (**c**) Global health status and function scales on day 15 of cycle 6. (**d**) Symptom and single-item scales on day 15 of cycle 6.

A high score on the global health status and functional scales indicates a good condition, while a high score on the symptom scale or single items indicates a worse condition. The data are presented as mean ± SD.


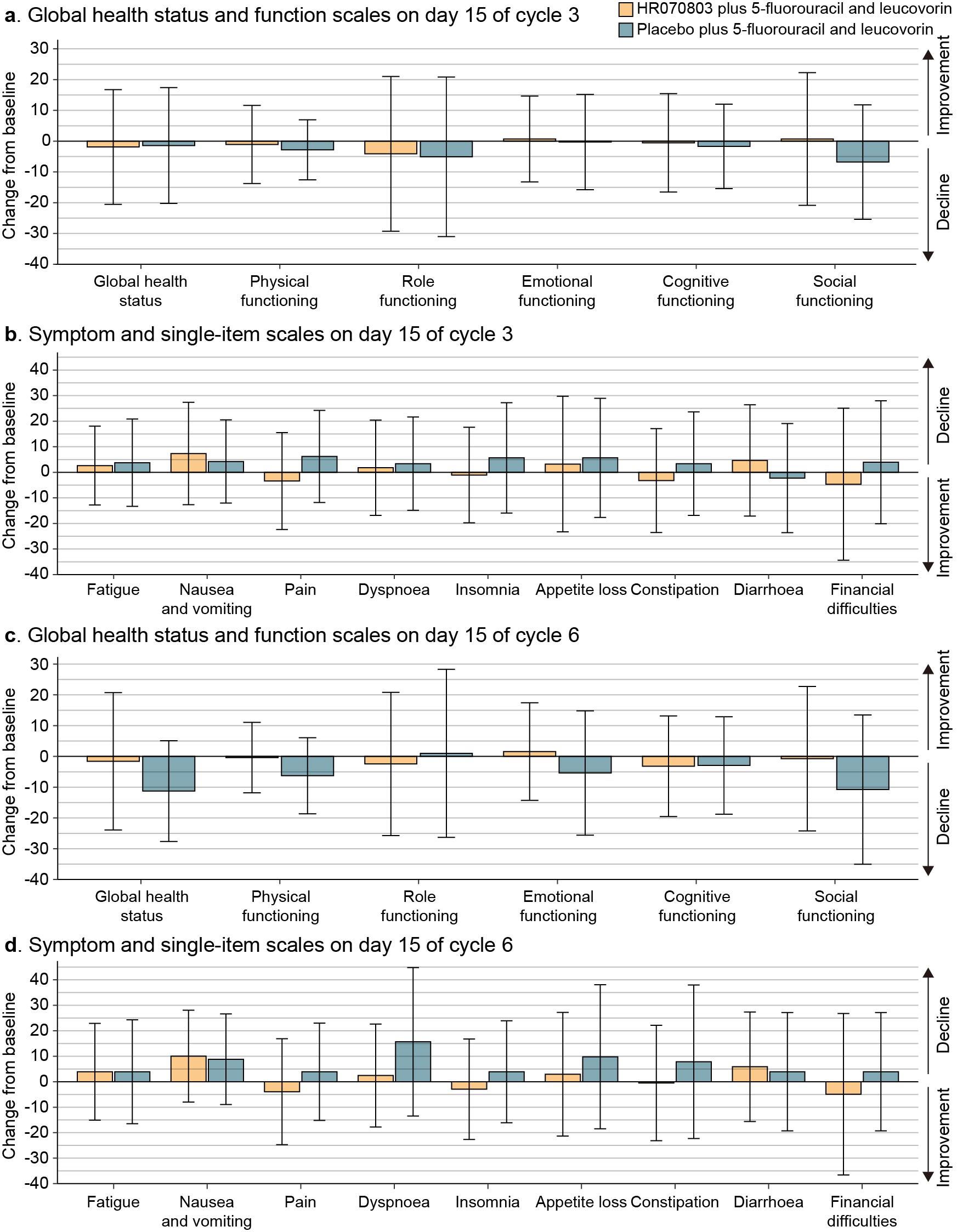


## List of participating sites

| **Investigator** | **Study site** | **Number of patients randomized** |
| --- | --- | --- |
| Liwei Wang | Renji Hospital, School of Medicine, Shanghai Jiaotong University | 31 |
| Yuhong Zhou | Zhongshan Hospital Affiliated with Fudan University | 21 |
| Shuang Zhang | West China School of Medicine/West China Hospital of Sichuan University | 18 |
| Xiaofeng Sun | Jiangsu Cancer Hospital | 16 |
| Zhendong Chen | The Second Affiliated Hospital of Anhui Medical University | 13 |
| Jiuwei Cui | The First Hospital of Jilin University | 11 |
| Weijia Fang | The First Affiliated Hospital, Zhejiang University School of Medicine | 10 |
| Kangsheng Gu | The First Affiliated Hospital of Anhui Medical University | 10 |
| Zhihua Li | Sun Yat-sen Memorial Hospital, Sun Yat-sen University | 10 |
| Jufeng Wang / Xiaobing Chen | The Affiliated Cancer Hospital, Zhengzhou University | 9 |
| Jun Yao | The First Affiliated Hospital of Henan University of Science and Technology | 9 |
| Jun Zhou | Peking University Cancer Hospital & Institute | 9 |
| Gang Wang | First Affiliated Hospital of University of Science and Technology of China | 8 |
| Yuxin Bai | Harbin Medical University Cancer Hospital | 7 |
| Shuikui Qin | Nanjing Tianyinshan Hospital, China Pharmaceutical University | 6 |
| Juxiang Xiao | The First Affiliated Hospital of Xi'an Jiaotong University | 6 |
| Wensheng Qiu | The Affiliated Hospital of Qingdao University | 6 |
| Bangmao Wang | Tianjin Medical University General Hospital | 6 |
| Yiping Mou | Zhejiang Provincial People's Hospital | 6 |
| Chunyue Wang | The First Affiliated Hospital of Xiamen University | 5 |
| Li Kong | Shandong Cancer Hospital | 5 |
| Jiajun Yin | Zhongshan Hospital Affiliated to Dalian University | 5 |
| Tao Zhang | Union Hospital, Tongji Medical College, Huazhong University of Science and Technology | 4 |
| Xionghu Shen | Affiliated Hospital of Yanbian University | 4 |
| Deliang Fu | Huashan Hospital Affiliated to Fudan University | 4 |
| Chuntao Gao | Tianjin Cancer Hospital | 4 |
| Mingzhi Zhang | The First Affiliated Hospital of Zhengzhou University | 3 |
| Xianglin Yuan | Tongji Hospital, Tongji Medical College, Huazhong University of Science and Technology | 3 |
| Jianping Xiong | The First Affiliated Hospital of Nanchang University | 3 |
| Minqiang Lu | Guangzhou First People's Hospital | 3 |
| Liqiang Zhong | The Second People's Hospital of Yibin | 3 |
| Baoshan Cao | Peking University Third Hospital | 3 |
| Yong Zha | Yunnan Cancer Hospital | 3 |
| Denghao Deng | North Jiangsu People's Hospital | 3 |
| Wei Cheng | Hunan Provincial People's Hospital | 3 |
| Yi Hu | Chinese People's Liberation Army (PLA) General Hospital | 2 |
| Zhiqiang Meng | Fudan University Shanghai Cancer Center | 2 |
| Wenxin Li | Inner Mongolia Autonomous Region People's Hospital | 2 |
| Guijie Liu | Liaocheng People's Hospital | 2 |
| Xiaoyu Yin | The First Affiliated Hospital, Sun Yat-sen University | 2 |
| Yusheng Wang | Shanxi Cancer Hospital | 2 |
| Liming Gao | The First Hospital of Qinhuangdao | 2 |
| Ting Fu | Zhuzhou Central Hospital | 2 |
| Shundong Cang | Henan Provincial People's Hospital | 2 |
| Yongqian Shu | The First Affiliated Hospital of Nanjing Medical University | 1 |
| Ying Cheng | Jilin Cancer Hospital | 1 |
| Yajin Chen | Sun Yat-sen Memorial Hospital, Sun Yat-sen University | 1 |
| Dongde Wu | Hubei Cancer Hospital | 1 |
| Xuening Ji | Zhongshan Hospital Affiliated to Dalian University | 1 |
| Rufu Chen | Guangdong General Hospital | 1 |
| Li Huang | The First Affiliated Hospital of Gannan Medical College | 1 |
| Aimin Zang | Affiliated Hospital of Hebei University | 1 |
| Xiaoyan Li | Beijing Tiantan Hospital Affiliated to Capital Medical University | 1 |
| Tao Wu | Anyang Tumour Hospital | 1 |
