# Supplementary material for: Irinotecan hydrochloride liposome HR070803 in combination with 5-fluorouracil and leucovorin in locally advanced or metastatic pancreatic ductal adenocarcinoma following prior gemcitabine-based therapy (PAN-HEROIC-1): a phase 3 trial
Source: Signal Transduct Target Ther. 2024 Sep 19;9:248. doi: 10.1038/s41392-024-01948-4 (PMC11412970; doi:10.1038/s41392-024-01948-4)
Supplement: Supplementary file 2 — Supplementary information_Protocol and Statistical analysis plan [file 41392_2024_1948_MOESM2_ESM.pdf]

(Original) Class 3.4 Chemical Drug **Clinical Approval No.: 2017L04358**

A RANDOMIZED, DOUBLE-BLIND, SINGLE-DUMMY, PARALLEL-CONTROLLED, MULTICENTER CLINICAL STUDY OF IRINOTECAN HYDROCHLORIDE LIPOSOME IN COMBINATION WITH 5-FU/LV AS SECOND-LINE TREATMENT FOR LOCALLY ADVANCED OR METASTATIC PANCREATIC CANCER AFTER TREATMENT FAILURE WITH GEMCITABINE-BASED THERAPY

Study No.: HR-IRI-APC

Version No.: 2.0

Version Date: 28 Jan., 2021

**Principal Investigators:**	**Shukui Qin, Professor**
	**Liwei Wang, Professor**
**Leading Site of Clinical Study:**	**Qinhuai Medical Area, Eastern Theater General Hospital of PLA China**
	**Renji Hospital, Shanghai Jiaotong University School of Medicine**
**Statistician:**	**Hao Yu, Professor**
**Statistical Institution:**	**Department of Biostatistics, School of Public Health, Nanjing Medical University**

Sponsor: Jiangsu Hengrui Pharmaceuticals Co., Ltd.

Confidentiality Statement

This document contains important confidential information proprietary to Jiangsu Hengrui Pharmaceuticals Co., Ltd., and should not be disclosed unless required by current laws or regulations. In any case, all personnel receiving this document should be notified of this confidential requirement and should not further disclose this information. This disclosure restriction also applies to all future confidential documents provided by the company.

Protocol Signature Page

Investigator Signature

I will carefully execute the duties as an investigator in accordance with the Chinese GCP, and personally participate in or directly lead this clinical study. I have read and confirmed this study protocol (Study No.: HR-IRI-APC; Version No.: 2.0; Version Date: 28 Jan., 2021). I agree to fulfill my duties in accordance with Chinese laws, the Declaration of Helsinki, the Chinese GCP, and this study protocol. Any modification to the protocol must be reviewed and approved by the sponsor, and can only be implemented upon approval by the Ethics Committee, unless measures must be taken to protect the safety, rights, and interests of the subjects.

Leading Site: Qinhuai Medical Area, Eastern Theater General Hospital of PLA China

Shukui Qin		
Principal Investigator (print)	Principal Investigator (signature)	Signature Date (DD/MM/YYYY)

Protocol Signature Page

Investigator Signature

I will carefully execute the duties as an investigator in accordance with the Chinese GCP, and personally participate in or directly lead this clinical study. I have read and confirmed this study protocol (Study No.: HR-IRI-APC; Version No.: 2.0; Version Date: 28 Jan., 2021). I agree to fulfill my duties in accordance with Chinese laws, the Declaration of Helsinki, the Chinese GCP, and this study protocol. Any modification to the protocol must be reviewed and approved by the sponsor, and can only be implemented upon approval by the Ethics Committee, unless measures must be taken to protect the safety, rights, and interests of the subjects.

Leading Site: Renji Hospital, Shanghai Jiaotong University School of Medicine

Liwei Wang		
Principal Investigator (print)	Principal Investigator (signature)	Signature Date (DD/MM/YYYY)

Protocol Signature Page

Investigator Signature

I will carefully execute the duties as an investigator in accordance with the Chinese GCP, and personally participate in or directly lead this clinical study. I have read and confirmed this study protocol (Study No.: HR-IRI-APC; Version No.: 2.0; Version Date: 28 Jan., 2021). I agree to fulfill my duties in accordance with Chinese laws, the Declaration of Helsinki, the Chinese GCP, and this study protocol. Any modification to the protocol must be reviewed and approved by the sponsor, and can only be implemented upon approval by the Ethics Committee, unless measures must be taken to protect the safety, rights, and interests of the subjects.

Study Site:

Principal Investigator (print)	Principal Investigator (signature)	Signature Date (DD/MM/YYYY)

Protocol Signature Page

Statistical Institution Signature

I have read and confirmed this study protocol (Study No.: HR-IRI-APC; Version No.: 2.0; Version Date: 28 Jan., 2021). I agree to fulfill my duties in accordance with Chinese laws, the Declaration of Helsinki, the Chinese GCP, and this study protocol. Any modification to the protocol must be reviewed and approved by the sponsor, and can only be implemented upon approval by the Ethics Committee, unless measures must be taken to protect the safety, rights, and interests of the subjects.

Statistical Institution: Department of Biostatistics, School of Public Health, Nanjing Medical University

Hao Yu		
Statistician (print)	Statistician (signature)	Signature Date (DD/MM/YYYY)

Protocol Signature Page

Sponsor Signature

I have read and confirmed this study protocol (Study No.: HR-IRI-APC; Version No.: 2.0; Version Date: 28 Jan., 2021). I agree to fulfill my duties in accordance with Chinese laws, the Declaration of Helsinki, the Chinese GCP, and this study protocol.

Sponsor: Jiangsu Hengrui Pharmaceuticals Co., Ltd.

Manager in Charge (print)	Manager in Charge (signature)	Signature Date (DD/MM/YYYY)

Study Protocol Revision History

Version No.	Version Date
1.0	8 Jul., 2017
1.1	25 Oct., 2019
2.0	28 Jan., 2021

TABLE OF CONTENTS

TABLE OF CONTENTS I

PROTOCOL SYNOPSIS VI

SCHEDULE OF ACTIVITIES XIII

LIST OF ABBREVIATIONS XVII

1. BACKGROUND 1

1.1 Epidemiology and Current Treatments of Pancreatic Cancer 1

1.2 Introduction to Irinotecan 1

1.3 Properties of Liposome 3

1.4 Overview of Clinical Studies of Onivyde (MM398/PEP-02) 4

1.4.1 Pharmacokinetic(s) 4

1.4.2 Phase I study 6

1.4.3 Phase II study 6

1.4.4 Phase III study 7

1.5 Information on Formulation Developed by Our Company 10

1.5.1 Nonclinical data 10

1.5.2 Phase Ia clinical study 10

1.5.3 Phase Ib clinical study 11

2. STUDY OBJECTIVES 12

2.1 Primary Objective 12

2.2 Secondary Objective 12

3. STUDY DESIGN 13

3.1 Overview of Study Design 13

3.2 Sample Size Estimation 13

3.3 Randomization Factors 14

3.4 Screening Number 14

3.5 Blinding and Procedures of Blinding Method 14

4. SELECTION AND WITHDRAWAL OF SUBJECTS 15

4.1 Diagnostic Criteria 15

4.2 Inclusion Criteria 15

4.3 Exclusion Criteria 17
4.4 Criteria for Subject Withdrawal/Treatment Discontinuation 18
4.5 Management of Subjects Experiencing Withdrawal/Treatment Discontinuation 18

5. STUDY TREATMENT 19

5.1 Drug Preparation 19
5.1.1 Irinotecan liposome 19
5.1.2 5-Fluorouracil and calcium folinate 19
5.2 Dosing Regimen 20

6. CRITERIA FOR DOSE INTERRUPTION AND MODIFICATION 20

6.1 General Principles for Modification 20
6.2 Dose Modification of Irinotecan Liposome 21
6.2.1 Dose modification due to hematologic toxicity 21
6.2.2 Dose modification due to non-hematologic toxicity 22
6.2.3 Modification due to special adverse reactions 23
6.3 Dose Modification of 5-FU and Calcium Folinate 25
6.3.1 Dose modification due to hematologic toxicity 25
6.3.2 Dose modification due to non-hematologic toxicity 26

7. STUDY DRUGS 26

7.1 Information on Study Drugs 26
7.2 Drug Packaging 28
7.3 Dispensation of Study Drugs 29
7.4 Management and Storage of Study Drugs 30
7.5 Handling and Return of Study Drugs 30
7.5.1 Handling of unused/expired drugs 30
7.5.2 Return of study drugs 30

8. RULES ON CONCOMITANT MEDICATIONS 31

8.1 Medications Prohibited or Used with Caution During the Study 31
8.1.1 Other anti-tumor drugs prohibited 31
8.1.2 Medications used with caution 31
8.2 Allowed Concomitant Medications and Treatments 31

9. STUDY PROCEDURES 32

9.1 Observation Items 32

9.2 Screening Period/Baseline 33

9.3 Visits in Treatment Period 35

9.3.1 Each cycle 35

9.4 End-of-Treatment or Withdrawal Visit 36

9.5 Safety Visit (30 ± 7 days after the end of treatment) 37

9.6 Visit for Subjects Who Discontinue Treatment/Withdraw for Non-PD Reasons 38

9.7 Survival Follow-up 38

9.8 Supplementary Instructions for Visit Examinations During the Study 39

9.9 End of Study 39

10. STUDY EVALUATION 39

10.1 Efficacy Evaluation 39

10.1.1 Primary efficacy endpoint and observation method 39

10.1.2 Secondary efficacy endpoints and observation methods 39

10.2 Safety Evaluation 41

10.2.1 Safety endpoints 41

10.2.2 Emergency unblinding 46

10.2.3 Treatment of common adverse reactions 46

11. INFORMED CONSENT AND ETHICAL STANDARDS 49

12. DATA MANAGEMENT 50

12.1 Case Report Form and Electronic Data Capture 50

12.2 Study Record Keeping 50

12.3 Data Management Procedures 51

12.4 Data Locking and Unblinding 51

13. STATISTICAL ANALYSIS METHODS AND DATA ANALYSIS 51

13.1 Definition of Analysis Sets 51

13.2 Statistical Analysis 52

13.2.1 Efficacy analysis 52

13.2.2 Safety analysis 55

13.2.3 Interim analysis 55

14. STUDY MANAGEMENT 56

14.1 Study Management Structure 56

14.2 Standard Operations 56

14.3 Training 56

14.4 Clinical Monitoring 56

14.5 Audit 57

14.6 Quality Control and Quality Assurance 57

14.7 Study Termination 58

14.7.1 Criteria for terminating the study at sub-sites 58

14.7.2 Criteria for terminating the entire study 58

15. DISCUSSION, APPROVAL, AND AMENDMENT OF STUDY PROTOCOL 59

16. PUBLICATION OF STUDY RESULTS 59

17. CLINICAL STUDY PROGRESS 60

18. REFERENCES 60

19. APPENDICES 62

Appendix 1 TNM Staging for Pancreatic Cancer 62

Appendix 2 ECOG Performance Status Scoring Criteria 63

Appendix 3 NRS Pain Scoring Criteria 64

Appendix 4 NYHA Function Classification 65

Appendix 5 Prohibited Traditional Chinese Medicine (TCM) 66

Appendix 6 Percent Bone Marrow Content in Human Skeleton 67

Appendix 7 Relevant Calculation Formulas 68

Appendix 8 Quality of Life Score 69

Appendix 9 Response Evaluation Criteria in Solid Tumors RECIST Version 1.1 72

List of Tables

Table 1.1. Pharmacokinetic parameters 4

Table 1.2. PEP02 in combination with 5-FU+LV in the treatment of metastatic pancreatic cancer after treatment failure with gemcitabine 7

Table 1.3. Incidences of adverse reactions of PEP02 in combination with 5-FU+LV in the treatment of pancreatic cancer (difference in incidence between the two groups: ≥ 5% for Grade 1-4, ≥ 2% for Grade 3-4) 8

Table 1.4. Incidences of laboratory test abnormalities induced by PEP02 in combination with 5-FU+LV in the treatment of pancreatic cancer (difference in incidence between the two groups ≥ 5%) 9

Table 6.1. Dose modification of irinotecan liposome due to neutropenia 21

Table 6.2. Dose modification of irinotecan liposome due to other hematologic toxicities 22

Table 6.3. Dose modification of irinotecan liposome due to non-hematologic toxicity 22

Table 6.4. Actions to be taken in case of infusion related reaction 23

Table 6.5. Dose modification of irinotecan liposome due to diarrhea 24

Table 6.6. Dose modification of 5-FU due to hematologic toxicity 25

Table 6.7. Dose modification of 5-FU due to non-hematologic toxicity 26

Table 10.1. PFS censoring for common progression or withdrawal 40

Table 10.2. ASCO guidelines for the treatment of chemotherapy-induced diarrhea 47

Table 13.1. Superiority boundaries in the interim analysis and final analysis 55

List of Figures

Figure 1. Structural formula of irinotecan hydrochloride 1

Figure 2. Overall survival 8

PROTOCOL SYNOPSIS

**Study Title**	A Randomized, Double-Blind, Single-Dummy, Parallel-Controlled, Multicenter Clinical Study of Irinotecan Hydrochloride Liposome in Combination with 5-FU/LV as Second-Line Treatment for Locally Advanced or Metastatic Pancreatic Cancer After Treatment Failure with Gemcitabine-Based Therapy
**Study Phase**	Confirmatory clinical study
**Version No.**	2.0
**Version Date**	28 Jan., 2021
**Sponsor**	Jiangsu Hengrui Pharmaceuticals Co., Ltd.
**Leadings Site**	Qinhuai Medical Area, Eastern Theater General Hospital of PLA China Shukui Qin<br>Renji Hospital, Shanghai Jiaotong University School of Medicine Liwei Wang
**Study Population**	Patients with locally advanced or metastatic pancreatic cancer who have failed gemcitabine-based therapy.
**Study Objectives**	● **Primary objective:**<br>To compare the overall survival (OS) of experimental group of irinotecan hydrochloride liposome in combination with 5-FU/LV vs. control group of 5-FU/LV<br>● **Secondary objective:**<br>To compare the following endpoints of experimental group vs. control group:<br>1) Progression-free survival (PFS)<br>2) Time to treatment failure (TTF)<br>3) Objective response rate (ORR)<br>4) CA19-9 tumor marker response<br>5) Quality of life score (EORTC-QTQ-C30)<br>6) Safety (adverse events and serious adverse events)
**Study Design**	This is a randomized, double-blind, single-dummy, parallel-controlled, multicenter clinical study to compare the efficacy and safety of irinotecan hydrochloride liposome in combination with 5-FU/LV vs. placebo in combination with 5-FU/LV as second-line treatment for locally advanced or metastatic pancreatic cancer after treatment failure with gemcitabine-based therapy. Approximately 272 eligible subjects will be randomized in a 1:1 ratio to either the experimental group (irinotecan liposome in combination with 5-FU/LV) or the control group (placebo in combination with 5-FU/LV), and will be randomly stratified according to the following stratification factors:<br>1) Albumin level (≥ 40 g/L vs. < 40 g/L);<br>2) History of fluorouracil therapy (with vs. without);<br>3) History of gemcitabine therapy (gemcitabine alone vs. gemcitabine combination).

	Subjects will be treated according to the randomized dosing regimen until progressive disease (PD) (radiographic or clinical deterioration) or intolerable toxicity occurs. After treatment discontinuation, subjects will undergo a 30-day follow-up period. Afterwards, subjects will be followed up once a month by telephone or other means. Subjects' survival status will be recorded until death or study closure (whichever occurs first). The primary endpoint will be analyzed after approximately 253 deaths occur. The entire study is planned to be completed in 36 months.<br><br>In this study, blinded and unblinded steering committees (SCs) are planned to be set up, and an interim analysis will be conducted. After data review, the unblinded SC will provide recommendation on whether to submit application for registration in advance. See SC Charter for detailed information on composition, responsibilities, and procedures of SC.
**Sample Size Estimation**	In this study, placebo + 5-FU/LV will be used as the control and a superiority test will be performed between the two groups, with OS as the primary efficacy endpoint for sample size estimation. An interim analysis is planned for this study.<br><br>With reference to the original NAPOLI-1 study data for pancreatic cancer (the OS was 6.1 months in the experimental group and 4.2 months in the control group) and the current status of pancreatic cancer in China, the median OS of the experimental group and the control group in this study is set as 5.0 months and 3.5 months, respectively, with significance level $\alpha = 0.025$ (one-sided), a power of 80%, the enrollment duration of 24 months, the total study duration of 36 months, and a dropout rate of 20%. A design of experimental group:placebo group = 1:1 will be adopted. An interim analysis is planned when 70% of OS events are collected to determine whether to conduct application for registration in advance. Based on the above parameters, at least 253 deaths should be collected and a total of 272 subjects should be enrolled (136 subjects in the experimental group and 136 subjects in the control group) according to the log-rank test for the OS comparison between the two groups and the O'Brien & Fleming type α spending function (EAST 6.5).
**Screening Criteria**	**Inclusion criteria**<br><br>Subjects meeting all of the following inclusion criteria are eligible to be enrolled in the study:<br><br>1. Male or female aged ≥ 18 years old;<br><br>2. Pathologically confirmed pancreatic cancer (derived from pancreatic ductal epithelium), also unresectable locally advanced or metastatic pancreatic cancer as shown in clinical records;<br><br>3. Treatment failure with gemcitabine-based systemic therapy as first-line treatment for locally advanced or metastatic disease (received at least 1 cycle of gemcitabine-based therapy, and developed PD or intolerance during treatment and PD after the end of treatment) as shown in clinical records, including but not limited to the following gemcitabine-based dosing regimens:<br><br>● Gemcitabine alone;<br><br>● Any gemcitabine-based regimen with or without maintenance treatment with gemcitabine;<br><br>● Gemcitabine alone, followed by combination with platinum, fluorouracil, erlotinib, etc.;

- Neoadjuvant/adjuvant chemotherapy containing gemcitabine, and relapse within 6 months after the completion of treatment.

Note: Definition of intolerance: 1) Hematologic toxicities: Grade 3 neutropenia with fever of > 38.5 °C, Grade 3 platelets decreased with hemorrhage symptoms, and other Grade 4 or greater hematologic toxicities; 2) Non-hematologic toxicities: Grade 3 or greater non-hematologic toxicities; 3) The above-mentioned toxicities render the subject unsuitable to continue the original therapy as judged by the investigator.

4. Have measurable or unmeasurable target lesions according to the Response Evaluation Criteria in Solid Tumors (RECIST) V1.1;
5. ECOG performance status of 0-1;
6. Life expectancy ≥ 12 weeks;
7. Adequate organ functions, i.e., meeting the following criteria:
   (1) Hematology test: (have not used hematopoietic growth factors or undergone blood transfusion within the last 7 days)
      a) Neutrophils ≥ 1.5 × 10^9/L;
      b) White blood cells ≥ 3.5 × 10^9/L;
      c) Platelets ≥ 100 × 10^9/L;
      d) Hemoglobin ≥ 90 g/L;
   (2) Biochemical test:
      e) Total bilirubin ≤ 1.5 × ULN (≤ 2.5 × ULN for subjects with biliary tract obstruction after biliary drainage);
      f) Aspartate aminotransferase (AST) and alanine aminotransferase (ALT) ≤ 2.5 × ULN (≤ 5 × ULN for subjects with liver metastases);
      g) Albumin level ≥ 30 g/L;
      h) Creatinine clearance ≥ 60 mL/min;
   (3) Cardiac function test:
      i) ECG normal or abnormal (without clinical significance as judged by the investigator);
      j) Left ventricular ejection fraction (LVEF) ≥ lower limit of normal (LLN);
8. Previous surgery, radiotherapy, chemotherapy, or other anti-tumor therapy ended 4 weeks ago or earlier, and general physical conditions or related adverse reactions have recovered (toxicity ≤ Grade 1) or reached a stable state;
9. Participate voluntarily and sign an informed consent form (ICF);
10. Have good compliance and agree to cooperate with survival follow-up.

Exclusion criteria

Subjects who meet any of the following criteria are ineligible to participate in this study:

1. Active central nervous system (CNS) metastases (including CNS metastases that are clinically symptomatic, have cerebral edema, and resulted in the use of steroids in the past 28 days or require it, and CNS metastases that have progressed);
2. Subjects with ascites who require clinical intervention (including subjects with moderate to large amount of ascites, such as subjects who should be stable for more than 4 weeks after drainage of ascites);
3. NRS pain score ≥ 4 after standardized treatment with painkillers;
4. Clinically significant gastrointestinal disorders (including hemorrhage, infective inflammation, perforation, obstruction, or Grade > 1 diarrhea);
5. Subjects who developed a second malignant tumor in the past 5 years (except those with cured carcinoma *in situ*, or basal or squamous cell skin cancer; subjects with other tumors that did not recur in the past 5 years can be enrolled);
6. Poorly controlled cardiovascular and cerebrovascular diseases or clinical symptoms, including but not limited to: (1) NYHA Class ≥ III cardiac failure; (2) unstable angina; (3) myocardial infarction or stroke in the past 6 months; (4) supraventricular or ventricular arrhythmia requiring treatment or intervention; (5) hypertension that is difficult to control (systolic blood pressure > 150 mmHg and/or diastolic blood pressure > 90 mmHg after optimal treatment).
7. Subjects with known active hepatitis B (HBsAg positive and HBV DNA ≥ 10^4 copies or ≥ 2000 u/mL);
8. Active infection or unexplained fever of > 38.5 °C in the screening period or on the day of administration that may affect the subject's participation in this study or interfere with efficacy evaluation according to the investigator's judgment (subjects with fever that is judged by the investigator to be caused by tumor can be enrolled);
9. Known allergy to any component of irinotecan liposome or other liposomes, 5-fluorouracil, or calcium folinate;
10. Have participated in clinical trials of other drugs within 4 weeks before the start of the study treatment or did not reach 5 half-lives of the previous investigational drug before study administration, whichever is longer;
11. Pregnant or lactating women;
12. Blood (urine) pregnancy test positive in the screening period for women of childbearing potential (male and female subjects should use reliable contraceptive measures to prevent pregnancy during the study and within 3 months after the last dose);
13. Other medical or social problems that may affect the subject's ability to sign the ICF, the subject's participation in the study, or the interpretation of study results as judged by the investigator.

Criteria for Withdrawal/Treatment Discontinuation

Treatment should be discontinued for subjects with the following conditions:

1) The subject's disease progressed according to RECIST V1.1;

2) The subject developed intolerable toxicity, or adverse events requiring:

	• the third dose reduction<br>• dose delay for more than 21 days from the start of the next cycle, unless the investigator believes that the subject can benefit from the treatment<br>3) The subject has obviously poor compliance and cannot follow the study protocol;<br>4) The subject withdraws the ICF and requests to withdraw from the study;<br>5) In consideration of the subject's rights, safety, and health, the investigator or the sponsor terminates the study or discontinues the treatment for the subject due to any reason in accordance with the GCP guidelines as well as laws and regulations.
**Method of Administration and Method of Administration**	● **Irinotecan liposome + 5-FU/LV group:**<br>Irinotecan liposome: 60 mg/m^2, intravenous infusion for at least 90 min, once every 2 weeks. For subjects homozygous for UGT1A1*28/*6 mutation, the starting dose should be reduced by one level (50 mg/m^2), and if no adverse reaction occurs in the first cycle, the dose of subsequent cycles can be elevated to the starting dose level.<br>LV: 200 mg/m^2, intravenous infusion for 30 ± 10 min, once every 2 weeks.<br>5-FU: 2000 mg/m^2, intravenous infusion for 46 ± 4 h, once every 2 weeks.<br>Administration sequence: in the order of irinotecan liposome, LV, and 5-FU.<br>Premedication: dexamethasone and antiemetics (or use other drugs that can prevent adverse reactions induced by irinotecan and 5-FU/LV according to the hospital's routine practice).<br>● **Placebo + 5-FU/LV group:**<br>Placebo: irinotecan liposome placebo, administered with the same method as that of irinotecan liposome.<br>LV: 200 mg/m^2, intravenous infusion for 30 ± 10 min, once every 2 weeks.<br>5-FU: 2000 mg/m^2, intravenous infusion for 46 ± 4 h, once every 2 weeks.<br>Administration sequence: in the order of placebo, LV, and 5-FU.<br>Premedication: dexamethasone and antiemetics (or use other drugs that can prevent adverse reactions induced by 5-FU/LV according to the hospital's routine practice).
**Evaluation Endpoints**	**Efficacy evaluation**:<br>**- Primary endpoint:**<br>To evaluate and compare the overall survival (OS) of the two groups.<br>**- Secondary endpoints:**<br>1. Progression-free survival (PFS);<br>2. Time to treatment failure (TTF);<br>3. Objective response rate (ORR);<br>4. CA19-9 tumor marker response;<br>5. Quality of life score (QoL);

	**Safety evaluation**:<br>Clinical symptoms and signs, laboratory tests, adverse events (AEs), and serious adverse events (SAEs), judged according to NCI CTCAE V4.03.
**Statistical Methods**	● **Efficacy analysis**<br>Overall survival, the primary endpoint of this study, is defined as the time from randomization to death due to any cause.<br>For the primary efficacy endpoint OS, the final analysis will be performed when 253 deaths (≥ 80% of subjects die) are collected in this study. In the analysis of the primary efficacy endpoint, the survival functions of the experimental group and the control group will be estimated by the Kaplan-Meier method, and the survival curve will be plotted. The survival functions of the two groups will be compared by the unstratified log-rank test. In addition, if the proportional hazard assumption is established between the two groups, a Cox model can be used to estimate the hazard ratio (HR) between the two groups and its overall 95% confidence interval (CI) will be calculated. For the secondary analysis of primary efficacy endpoint OS, a Cox proportional hazards model will be used to estimate the HR and its 95% CI will be calculated, taking into account the important covariates such as randomization stratification factors.<br>Also, an analysis method similar to above will be used for the statistics of PFS and TTF.<br>In addition, the sensitivity analysis of potential prognostic factors will be performed using a Cox proportional hazards regression model for the OS, PFS, and TTF.<br>Other secondary efficacy endpoints, including ORR, change in CA19-9 level, and EORTC QLQ-C30, will be analyzed descriptively, and the differences between the two groups will also be compared and analyzed.<br>● **Safety analysis**<br>Descriptive statistical analysis will be mainly used. The AEs and SAEs, treatment-related AEs and SAEs, as well as AEs and SAEs specific to the drug will be listed and summarized. When necessary, the incidence and severity of AEs and SAEs will be compared between groups using Fisher's exact test.<br>The analysis of safety data involves the dose and drug exposure in treated subjects.
**Interim Analysis**	In this study, one interim analysis will be performed for the primary efficacy endpoint. The main objectives of the interim analysis include but are not limited to:<br>1. Application for registration in advance due to superiority;<br>2. Continuation of the study as planned.<br>The interim analysis will be conducted when 70% of endpoint events (177) are collected. The superiority boundaries determined using the O'Brien & Fleming type α spending function are as follows:<br>(see table below)

Time Point	Number of Endpoint (OS) Events	Superiority Boundary Z-Value (Corresponding HR)	One-Sided Nominal Significance Level of Superiority
Interim Analysis	177 (70%)	-2.438 (HR = 0.693)	0.007
Final Analysis	253	-2.000 (HR = 0.777)	0.023

	Note: OS = Overall Survival;<br><br>The interim analyses will be completed by unblinded statisticians and their programming team. The interim analysis results will be reviewed by the unblinded SC, and the SC will determine whether to put forward the proposal of application for registration in advance to the sponsor according to the results. If the sponsor applies for registration in advance, the early unblinding will be conducted at the same time (see 12.4 for details).
**RECIST Version**	Version 1.1
**CTCAE Version**	Version 4.03

SCHEDULE OF ACTIVITIES

Study Procedure	Screening Period[1] (D-14 to D0)	Every Cycle		Every 6 Weeks ± 1 Week	End of Treatment/ Withdrawal	30-Day Follow-up ± 1 Week	Survival Follow-up[17] (Every Month) ± 1 Week
		D1	D15±2				
**Basic Information**							
Informed Consent	X						
Demographics	X						
Tumor History/Other Medical History	X						
Physical Examination	X	X	X		X	X	
Vital Signs	X	X	X		X	X	
**Genomics Examination**[2]							
UGT1A1*28/*6 Gene	X						
**Laboratory Tests**							
Urinalysis	X				X		
Hematology	X		X		X[14]	X	
Blood Biochemistry[3]	X		X		X[14]	X	
Coagulation Function	X		X		X[14]	X	
ECG[4]	X				X		
Echocardiography	X						
Hepatitis B Virus Test[5]	X						
Pregnancy Test[6]	X				X		
**Tumor Markers**[7]							
CA19-9 Test	X			X	X[15]	X	

Study Procedure	Screening Period[1] (D-14 to D0)	Every Cycle		Every 6 Weeks ± 1 Week	End of Treatment/ Withdrawal	30-Day Follow-up ± 1 Week	Survival Follow-up[17]
		D1	D15±2				(Every Month) ± 1 Week
CA 12-5 Test	X			X	X[15]	X	
CEA Test	X			X	X[15]	X	
**Imaging Examination**							
Tumor Evaluation[7]	X			X[7]	X[16]		
**Other Clinical Evaluations and Examinations**							
Weight	X[1]	X	X		X	X	
ECOG PS	X	X	X		X	X	
NRS Pain Score	X	X	X		X	X	
Quality of Life Score[8]	X		X[8]		X[15]	X	
Dispensation/Return of Subject Diary Card[9]		X	X		X		
**Safety Evaluation**							
Adverse Events[10]	X	X	X		X	X	
Concomitant Medication[11]	X	X	X		X	X	
**Study Drugs**							
Randomization	X						
Premedication[12]		X					
Injection of Irinotecan Liposome, 5-FU, and LV[13]		X					
**Survival Follow-up**							
Anti-Tumor Treatment						X	X
Time of Death						X	X

Precautions:

(1) The screening period is 14 days, during which the subject's body weight change should be monitored in due time based on clinical needs;

(2) Centralized unified testing will be adopted for genomics examination;

(3) Blood biochemistry: including liver function, kidney function, electrolyte, blood lipid, and blood glucose tests; for details, see "Observation Items" in "STUDY PROCEDURES" of the protocol;

(4) ECG: Additional examinations can be performed according to clinical requirements during the study;

(5) Hepatitis B virus test: hepatitis B surface antigen (HBsAg), hepatitis B surface antibody (HBsAb), hepatitis B e antigen (HBeAg), hepatitis B e antibody (HBeAb), and hepatitis B core antibody (HBcAb); if HBsAg is positive, HBV-DNA load should be quantified;

(6) Pregnancy test: Women of childbearing potential must undergo a blood/urine pregnancy test within 7 days prior to enrollment, and at any time when pregnancy is suspected during the study, a pregnancy test is required;

(7) Tumor marker test and imaging evaluation: once every 6 weeks (± 1 week) after randomization, even if there is a dose delay or dose discontinuation;

(8) Quality of life score: evaluated once every 3 cycles; if the scoring is required on the day of administration, perform it before administration;

(9) Subject diary card: The subject's daily discomfort and medication should be recorded, including the dose of painkillers, as well as other supportive care and AE treatment;

(10) AEs/SAEs should be continuously recorded during the study until 30 days after the last dose of investigational drug. If the AEs/SAEs still persist, they need to be recorded until recovery to normal or baseline level;

(11) Concomitant medication: Various treatments and drugs used from 30 days before signing the informed consent form to 30 days after the last dose should be recorded;

(12) Premedication: dexamethasone and antiemetics (or use other drugs that can prevent adverse reactions induced by irinotecan and 5-FU/LV according to the hospital's routine practice);

(13) The experimental group is given irinotecan liposome + 5-FU/LV and the control group is given placebo + 5-FU/LV. During the irinotecan liposome infusion, adverse reactions should be closely monitored;

(14) If the subject does not receive this examination within the last 1 week before the end of treatment/withdrawal, this examination should be performed;

(15) If the subject does not receive this examination within the last 4 weeks before the end of treatment/withdrawal, this examination should be performed;

(16) If the subject withdraws from the study due to non-PD reasons, a tumor evaluation should be performed as soon as possible after the end of the study (at least within the 30-day follow-up period), unless a tumor evaluation has been done within 4 weeks before the end, so as to confirm that no PD occurs and evaluate the overall status of the disease. Afterwards, the tumor evaluation should be performed once every 6 weeks until PD or start of other anti-tumor treatments;

(17) Survival follow-up can be performed by telephone to inquire about the subsequent anti-tumor treatment regimen and survival information of the subject.

LIST OF ABBREVIATIONS

The following abbreviations and terms are used in this study protocol:

Abbreviation	Full Name
AE	Adverse event
APTT	Activated partial thromboplastin time
BP	Blood pressure
CFDA	China Food and Drug Administration (now National Medical Products Administration)
CRF	Case report form
CRO	Contract Research Organization
CPT-11	Irinotecan
DF	Drug concentration fluctuation coefficient
DLT	Dose limiting toxicity
EC	Ethics Committee
ECG	Electrocardiogram
EMEA	European Medicines Agency
EPR	Enhanced permeability and retention effect
GCP	Good Clinical Practice
HB	Hemoglobin
HBsAg	Hepatitis B surface antigen
HCT	Hematocrit
HCV	Hepatitis C virus
HIV	Human immunodeficiency virus
HR	Hazard ratio
ICF	Informed consent form
INR	International normalized ratio
IVRS	Interactive voice response system
LV	Calcium folinate
MRT	Mean residence time
MTD	Maximum tolerated dose
NRS	Numerical rating scale
ORR	Objective response rate
OS	Overall survival
PD	Progressive disease
PK	Pharmacokinetic(s)
PR	Partial response

Abbreviation	Full Name
QA	Quality assurance
QC	Quality control
SAE	Serious adverse event
SC	Steering committee
SD	Stable disease
SOC	System organ class
SOP	Standard operating procedure
SN-38	7-ethyl-10-hydroxycamptothecine
T1/2	Half-life
TGF	Transforming growth factor
TIA	Transient ischemic attack
TTF	Time to treatment failure
ULN	Upper limit of normal
5-FU	5-fluorouracil

1. BACKGROUND

1.1 Epidemiology and Current Treatments of Pancreatic Cancer

Pancreatic cancer has a high degree of malignancy and extremely poor prognosis. Approximately 60% of pancreatic cancer patients have distant metastases at the time of diagnosis and 25% of patients are at locally advanced stage, for whom radical resection is infeasible and drug therapy is mainly used. Drug therapy is predominated by chemotherapy. There is a lack of targeted drugs in clinical practice. Systemic therapy is recommended for locally advanced unresectable and metastatic pancreatic cancer according to NCCN guidelines. Some first-line combination chemotherapy regimens (such as FOLFIRINOX or gemcitabine + albumin-bound paclitaxel) can be selected for patients with good functional status. However, for patients with poor functional status, gemcitabine monotherapy is still the first choice at present. In China, first-line treatment mostly adopts gemcitabine monotherapy regimen, while second-line chemotherapy adopts fluorouracil-based regimen (for patients who failed gemcitabine first-line treatment) or gemcitabine-based regimen (for patients who failed fluorouracil-based first-line treatment).

In Oct. 2015, as an orphan drug granted priority review, Merrimack's liposomal irinotecan (Onivyde) in combination with 5-FU/LV was approved by the FDA to be used as a second-line regimen for the treatment of metastatic pancreatic cancer after failure of gemcitabine therapy. With this regimen, the mean survival was 6.1 months, 1.9 months longer than that of the 5-FU/LV control group (4.2 months). This treatment regimen has been included in the NCCN treatment guidelines and is expected to become the standard second-line chemotherapy regimen for advanced pancreatic cancer.

Similarly, our company has developed irinotecan liposome formulation on the basis of the available dosage forms of irinotecan. It is hoped that by changing the dosage form, the drug's distribution and metabolism in the body and in the tumor can be improved, thus enhancing efficacy and reducing toxicities.

1.2 Introduction to Irinotecan

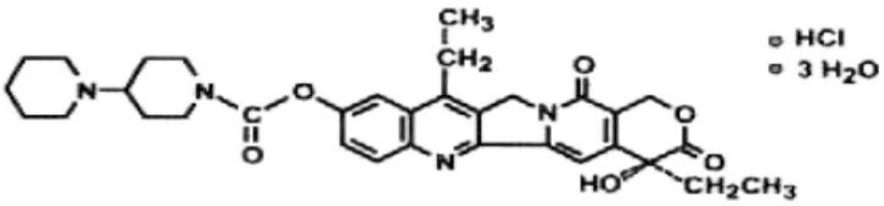

Figure 1. Structural formula of irinotecan hydrochloride

Irinotecan is a semi-synthetic derivative of camptothecine, and its hydrochloride (irinotecan hydrochloride, structural formula shown in Figure 1) has become a commonly used cytotoxic antineoplastic drug in clinical practice. In 1966, camptothecine, a substance with anti-tumor activity, was extracted from *Camptotheca acuminata*, a tree native to China. However, due to its poor water solubility and severe toxicity, the research was once interrupted, limiting its further application. With the discovery of camptothecine's mechanism of action and special therapeutic targets, Japan's Yakult Honsha and Daiichi Sankyo jointly developed irinotecan hydrochloride (CPT-11). CPT-11 was approved by the US FDA in Nov. 1996 for the third-line treatment of colorectal cancer; in 1998, it was approved for the second-line treatment of colorectal cancer patients who failed the therapy with fluorouracil (5-FU); in 2000, it was approved to be used in combination with 5-FU/LV for the first-line treatment of advanced colorectal cancer. In more than 40 years, CPT-11 is the second chemotherapy drug approved by the US FDA for the first-line treatment of metastatic colorectal cancer following 5-FU. The drug was marketed in China in Mar. 2001.

The current commercially available dosage forms of irinotecan are irinotecan hydrochloride injection and its lyophilized powder for injection. In clinical practice, after intravenous administration, the free drug gets directly into a relatively alkaline physiological environment, and the lactone ring in its structure is prone to hydrolyze into carboxylate form, thereby losing activity and indirectly reducing efficacy. Moreover, the dosage forms have marked toxic and side effects, mainly manifested as severe neutropenia and severe delayed diarrhea. Among patients treated with CPT-11, severe diarrhea occurred in 20% of patients with diarrhea requiring treatment; 78.7% experienced neutropenia, of which 22.6% were severe cases (neutrophil count $< 500/mm^3$). Nausea, vomiting, and other gastrointestinal reactions were common. Despite the use of antiemetics, 10% of patients developed severe nausea and vomiting; anemia, acute cholinergic syndrome, and other toxic and side effects were also observed. As a drug carrier for a new dosage form, liposome encapsulates irinotecan in the aqueous phase of liposome, protecting the active structure of the lactone ring while promoting the targeting distribution of the drug and achieving sustained release. The results of foreign clinical studies showed that liposomalization can significantly enhance the anti-tumor efficacy of irinotecan and reduce its toxicity.

1.3 Properties of Liposome

In recent years, with the gradual deepening of studies on high-efficacy and low-toxicity targeting drug-delivery systems by scholars in China and abroad, anti-tumor drug liposomes have become a focus of research and development. Liposomes can be classified into multilamellar vesicles (MLV, particle size: 200-5000 nm), small unilamellar vesicles (SUV, particle size: about 30 nm), and large unilamellar vesicles (LUV, particle size: 100-2000 nm) based on shape and size. The particle size of liposomes may have a large impact on the *in vivo* distribution of the product, and directly affect the efficacy and adverse reactions of the drug. When the carrier particle size is large, drugs are mainly phagocytized by the reticuloendothelial system (RES); when the particle size is small (those with particle size < 100 nM are also called "stealth" liposomes), liposomal drugs can achieve targeting distribution in tumor tissues through enhanced permeability and retention (EPR) effect, while reducing the uptake by RES.

The liposomes developed by our company have an average particle size of < 100 nM ("stealth" liposomes), which can reduce the uptake by RES and contribute to the targeting distribution in tumors. In addition, liposomes have the following functions:

Protection: Some drugs have poor stability, such as camptothecine derivatives. Under relatively alkaline physiological conditions, these drugs are unstable and the saturated lactone ring in their structure will transform into carboxylate form, but the lactone ring is an essential structure for their anti-tumor activity. Therefore, after being wrapped by liposomes, they are encapsulated in the water phase of liposomes by virtue of the special bilayer structure and properties of liposomes to effectively protect the lactone ring.

Passive targeting: Targeting is the most prominent feature of liposomes as drug carriers, and passive targeting (natural targeting) is the basic feature of liposomes when they are administered intravenously. After liposomes enter the human body: (1) they may be phagocytized by RES, resulting in the accumulation of liposomal drugs in organs rich in reticuloendothelial cells such as liver, spleen, and lymph, but also reducing the concentration in kidneys, heart, and brain tissues; (2) they may accumulate in solid tumor sites through the EPR effect: In normal tissues, due to the dense microvascular endothelial space and complete structure, macromolecules and lipid granules are not easy to permeate, while in solid tumor tissues, due to abundant blood vessels, wide vascular wall space, incomplete structure, and lack of lymphatic return, solid tumors have selective high permeability and retention for macromolecules and lipid granules. This phenomenon is called the enhanced permeability and retention (EPR) effect of solid tumor tissues. In addition, other pathways are also at work: some tumor cells have strong endocytosis and directly phagocytize liposomal drugs; drugs diffuse into tumors from the circulatory system or para-tumor tissues; drugs are phagocytized by monocytes in the circulatory system and then transferred into tumors. In

comparison with conventional dosage forms, the targeting of the liposomal drug-delivery system for solid tumors may significantly improve the efficacy of drugs.

Long-acting effect: Liposomes, as drug carriers, slowly release drugs to achieve the effect of sustained release and act continuously in the cell growth cycle, thereby enhancing the therapeutic index. Since liposomes act as drug carriers to allow slow drug release, they can be considered drug depots.

1.4 Overview of Clinical Studies of Onivyde (MM398/PEP-02)

1.4.1 Pharmacokinetic(s)

Absorption: The plasma concentrations of total irinotecan and total SN-38 after Onivyde monotherapy or combined administration over the dose range of 50-155 mg/m^2 in 353 tumor subjects were evaluated with the study approach of population pharmacokinetics (PK). Over the dose range of 50-155 mg/m^2, the C_{max} and AUC of total irinotecan increased with the increasing dose; the C_{max} of metabolite SN-38 increased proportionally with increasing dose, but the AUC of SN-38 was not dose proportional.

The PK parameters of total irinotecan and total metabolite SN-38 after combined or single-agent administration at 70 mg/m^2 are shown in Table 1.1.

Table 1.1. Pharmacokinetic parameters

Dose Group (mg/m^2)	Total Irinotecan					Total SN-38		
	C_{max} (μg/mL)	AUC_{last} (μg/mL·h)	$t_{1/2}$ (h)	CL (L/h)	Vd (L)	C_{max} (ng/mL)	AUC_{last} (ng/mL·h)	$t_{1/2}$ (h)
70	37.2±8.8	1364±1048	25.8±15	0.2±0.17	4.1±1.5	5.4±3.4	620±329	67.8±44.5

Distribution: After administration, 95% of irinotecan was encapsulated in liposomes, and at 169.5 h after administration, the encapsulation rate of irinotecan began to decrease. At the dose of 70 mg/m^2, the apparent volume of distribution (Vd) was 4.1 ± 1.5 L. The irinotecan-protein binding rate was < 0.44%.

Metabolism: The metabolism of irinotecan liposome has not been studied. Irinotecan (hereinafter referred to as CPT-11) is eliminated in the body mainly through liver metabolism, biliary secretion, and urine excretion. *In vivo* metabolic enzymes primarily include: carboxylesterases (CEs), glucuronosyltransferases (UGTs), cytochrome oxidase (CYP), and β-glucuronidase. Under the action of CEs, CPT-11 transforms into active metabolite SN-38 (7-ethyl-10-hydroxycamptothecine), but CPT-11 and SN-38 have poor stability. In a relatively alkaline physiological environment, the lactone ring in their structure is prone to hydrolyze into carboxylate form, thereby losing activity. CPT-11 can also transform into inactive oxidative

products under the action of CYP. Secondary metabolite SN-38 transforms into SN-38G (glucuronide) under the action of UGTs. UGT1A1*28 genotype subgroups were analyzed by population PK. A low dose was given to heterozygous patients. The steady-state blood concentrations of SN-38 were comparable between homozygous subjects (N = 14, 1.06 ng/mL) and non-homozygous subjects (N = 244, 0.95 ng/mL).

Excretion: The *in vivo* excretion of irinotecan liposome remains unclear. The amount excreted via urine was 11%-20% of the injection dose of irinotecan hydrochloride, < 1% for SN-38, and 3% for SN-38 glucuronide. After infusion of irinotecan hydrochloride in two subjects, the cumulative excretion of irinotecan hydrochloride and its metabolites (SN-38 and SN-38 glucuronide) in bile and urinary tract was approximately 25% (100 mg/m^2) to 50% (300 mg/m^2) after 48 h.

Special population - race: As analyzed by population PK, the steady-state blood concentration of total irinotecan in Asians was 56% lower than that in Caucasians, and that of SN-38 was 8% higher than that in Caucasians (Asians N = 150, Caucasians N = 182).

Special population - liver injury: The metabolic characteristics of irinotecan liposome have not been studied in subjects with hepatic insufficiency. As analyzed by population PK, the steady-state blood concentration of total SN-38 in subjects with total bilirubin of 1-2 mg/dL at baseline (N = 19) was 37% higher than that in subjects with total bilirubin of < 1 mg/dL at baseline, but the increase in ALT/AST had no effect on the steady-state blood concentration of SN-38. There are no relevant study data in subjects with total bilirubin of > 2 mg/dL.

Drug-drug interaction: It was found in the population PK study that the PK parameters of total irinotecan and SN-38 were not affected by co-administered 5-fluorouracil and calcium folinate. When irinotecan was administered after dexamethasone premedication, dexamethasone did not affect the metabolism of irinotecan. *In vitro* studies showed that irinotecan and its metabolites SN-38 and aminopentane carboxylic acid had no inhibitory effect on P-450.

Pharmacogenomics: The risk of developing neutropenia is higher in subjects homozygous for UGT1A1*28 mutation. In the phase III clinical study, homozygous subjects (N = 7) received treatment at a starting dose of 50 mg/m^2, and 28.6% of them (2/7) developed Grade 3/4 neutropenia; non-homozygous subjects (N = 110) received treatment at a starting dose of 70 mg/m^2, and 27.3% (30/110) developed Grade 3/4 neutropenia. The incidences were comparable.

1.4.2 Phase I study

Solid tumors, phase I: Phase I study of PEP-02 in subjects with advanced solid tumors. A total of 11 subjects were enrolled and received treatment at the escalating doses of 60, 120, 180, and 240 mg/m^2, with 1, 6, 4, and 0 subjects in each group, respectively. Dosing regimen: 90-min intravenous infusion (D1), 3 weeks/cycle. DLTs were observed in 3 subjects in the 180 mg/m^2 dose group, of whom 1 subject experienced Grade 3 infection caused by urinary catheter, 1 subject experienced Grade 3 diarrhea and granulocytopenia accompanied with fever, and 1 subject experienced Grade 4 diarrhea and neutropenia. Therefore, the MTD was determined to be 120 mg/m^2. PK data of 120 mg/m^2 dose group: unchanged irinotecan: CL (0.0591 L/m^2/h), Vd (1.8 L/m^2), and $t_{1/2}$ (29.5 h); SN-38 [compared with published data (irinotecan: 125 mg/m^2)]: C_{max}: 9.2 ± 3.5 vs. 26.3 ± 11.9 ng/mL, $t_{1/2}$: 75.4 ± 43.8 vs. 10.4 ± 3.1 h, and AUC: 710 ± 395 vs. 229 ± 108 ng·h/mL. Among the 10 evaluable subjects, 1 subject achieved PR and 3 subjects achieved SD. Conclusion: The recommended phase II dose is 120 mg/m^2.

1.4.3 Phase II study

PEPCOL, colorectal cancer: In 2011, a study on second-line treatment for mCRC (PEPCOL study) was carried out in 6 sites in France and other countries/regions. A total of 55 subjects were enrolled, of whom 28 were randomized to receive FUPEP (PEP02 + 5FU/LV) and 27 were randomized to receive FOLFIRI (FOLFIRI-1 or FOLFIRI-3). The ORR was 16.7% (n = 4/24) in the FUPEP group and 11.5% (n = 3/26) in the FOLFIRI group. Grade 3-4 AEs in both groups were diarrhea (21% vs. 33%), neutropenia (11% vs. 30%), mucositis (11% vs. 11%), and alopecia (G2: 25% vs. 26%). The FUPEP regimen has shown preliminary safety and efficacy, and it is worth looking forward to as second-line treatment for mCRC. In addition, based on the results, the FUPEP treatment group was added in the phase III NAPOLI-1 study on metastatic pancreatic cancer.

Pancreatic cancer: In 2009, a multicenter, single-arm, Simon's two-stage study on second-line treatment for metastatic pancreatic cancer (subjects who failed gemcitabine-based first-line chemotherapy) was carried out, with 3-month survival rate as the primary efficacy endpoint. Subjects received monotherapy at 120 mg/m^2 by 90-min intravenous infusion, with 21 days as a cycle. A total of 40 subjects were enrolled. Efficacy results: The 3-month, 6-month, and 1-year survival rates were 75%, 43%, and 25%, respectively. The median OS was 5.2 months and the median PFS was 2.4 months. Three subjects (7.5%) achieved objective response and 17 subjects (42.5%) achieved stable disease for ≥ 2 cycles. The average treatment cycle was 5.4 cycles (range: 1-26 cycles). Safety: Common adverse events were neutropenia, abdominal pain, fatigue, and diarrhea; Grade 3/4 hematologic toxicity was mainly neutropenia (incidence: 30%), and Grade 3/4 non-hematologic toxicities were diarrhea (15%), nausea (10%), and fatigue (20%). No acute

cholinergic syndrome occurred (no atropine was used). PEP02 monotherapy has shown good efficacy and controllable safety in the treatment of metastatic pancreatic cancer after treatment failure with gemcitabine.

1.4.4 Phase III study

Pancreatic cancer, phase III: NAPOLI-1 was an open-label, international, multicenter, phase III study evaluating PEP02 in combination with 5-FU/LV in the treatment of metastatic pancreatic cancer after treatment failure with gemcitabine. A total of 417 subjects were enrolled, of whom 151 subjects were allocated to the PEP02 monotherapy arm (arm 1), 149 subjects were allocated to the 5-FU/LV arm (arm 2), and 117 subjects were allocated to the PEP02 plus 5-FU/LV arm (arm 3). Arm 3 was added after protocol modification, and 119 subjects were newly enrolled to arm 2 after protocol modification. These subjects were used for evaluation and comparison with the combination treatment arm. The OS of the three arms was 4.9, 4.2, and 6.1 months, respectively. There was no significant difference between arm 1 and arm 2, with HR = 0.99 and P = 0.5545; the OS of arm 3 was significantly higher than that of arm 2, with HR = 0.68 and P = 0.0009. In terms of other efficacy endpoints including PFS, ORR, and CA19-9 reduction, the results of arm 3 were significantly superior to those of arm 2. See Table 1.2 and Figure 2 for specific data. Adverse reactions were safe and controllable. The main common Grade 3-4 adverse reactions were neutropenia, diarrhea, nausea, vomiting, fatigue, anorexia, and stomatitis. See Table 1.3 and Table 1.4 for specific data.

Table 1.2. PEP02 in combination with 5-FU+LV in the treatment of metastatic pancreatic cancer after treatment failure with gemcitabine

Item	PEP02+5-FU/LV (N=117)	5-FU/LV (N=119)	*p* Value
mPFS, Months (95% CI)	3.1 (2.7-4.2)	1.5 (1.4-1.8)	0.0001 (Log-rank test)
12-Week PFS Rate, % (95% CI)	57 (47-66)	26(18-35)	-
Objective Response Rate, % (95% CI)	16 (9.6-22.9)	1 (0.0-2.5)	＜0.001 (Fisher's exact test)
mOS, (95% CI)	6.1 (4.8-8.9)	4.2 (3.3-5.3)	0.0009
CA19-9 Reduction, %	36 (27/76)	12 (8/69)	0.0009 (Fisher'sexact test)

Note: The ONIVYDE monotherapy and 5-FU/LV groups showed no significant improvement in overall survival (4.9 months vs. 4.2 months)

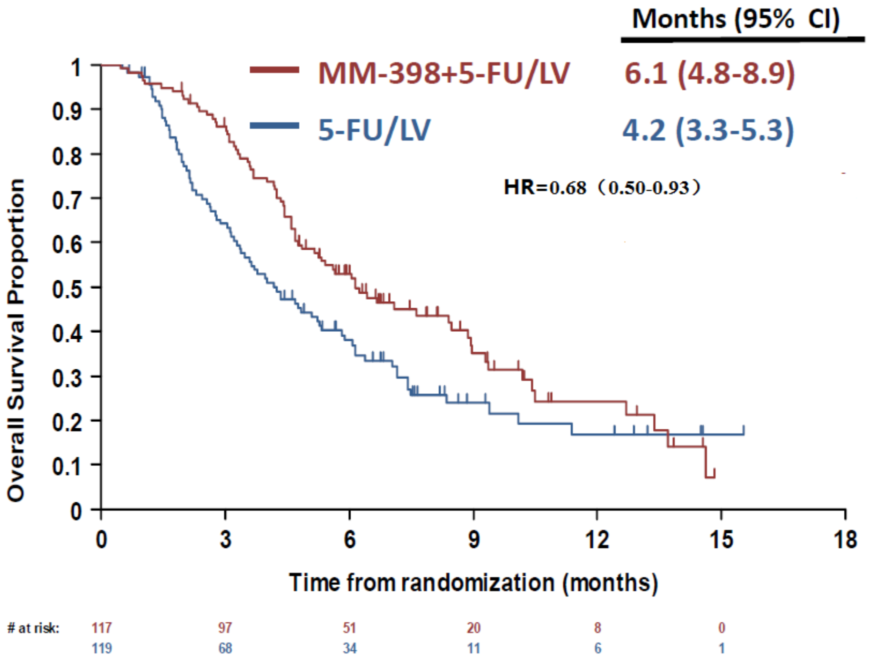

Figure 2. Overall survival

Table 1.3. Incidences of adverse reactions of PEP02 in combination with 5-FU+LV in the treatment of pancreatic cancer (difference in incidence between the two groups: ≥ 5% for Grade 1-4, ≥ 2% for Grade 3-4)

Adverse Reaction	**ONIVYDE plus 5-FU+LV** N = 117		**5-FU+LV** N=134	
	Grade 1-4 (%)	Grade 3-4 (%)	Grade 1-4 (%)	Grade 3-4 (%)
**Gastrointestinal Disorders**				
Diarrhea	59	13	26	4
Early onset diarrhea	30	3	15	0
Delayed diarrhea	43	9	17	4
Vomiting	52	11	26	3
Nausea	51	8	34	4
Stomatitis	32	4	12	1
**Infection**				
Sepsis	4	3	2	1
Neutropenia with Fever	3	3	1	0
Viral Gastroenteritis	3	3	0	0
Inflammation Induced by Venous Duct	3	3	0	0
**General Disorders and Administration Site Conditions**				
Fatigue/Weakness	56	21	43	10

Adverse Reaction	ONIVYDE plus 5-FU+LV N = 117		5-FU+LV N=134	
	Grade 1-4 (%)	Grade 3-4 (%)	Grade 1-4 (%)	Grade 3-4 (%)
Feverish	23	2	11	1
**Metabolism and Nutrition Disorders**				
Anorexia	44	4	32	2
Weight Decrease	17	2	7	0
Dehydration	8	4	7	2
**Skin and Subcutaneous Tissue Disorders**				
Baldness	14	1	5	0

Graded as per NCI CTCAE V4

Table 1.4. Incidences of laboratory test abnormalities induced by PEP02 in combination with 5-FU+LV in the treatment of pancreatic cancer (difference in incidence between the two groups ≥ 5%)

Adverse Reaction	ONIVYDE plus 5-FU+LV N = 117		5-FU+LV N=134	
	Grade 1-4 (%)	Grade 3-4 (%)	Grade 1-4 (%)	Grade 3-4 (%)
**Blood Disorders**				
Anemia	97	6	86	5
Lymphocytes Decreased	81	27	75	17
Neutropenia	52	20	6	2
Platelets Decreased	41	2	33	0
**Hepatic Disorders**				
Glutamic-pyruvate Transaminase Increased (ALT)	51	6	37	1
Hypoalbuminemia	43	2	30	0
**Metabolism and Nutrition Disorders**				
Hypomagnesemia	35	0	21	0
Hypokalemia	32	2	19	2
Hypocalcemia	32	1	20	0
Hypophosphatemia	29	4	18	1
Hyponatremia	27	5	12	3
**Renal Disorders**				
Creatinine Increased	18	0	13	0

Graded as per NCI CTCAE V4

1.5 Information on Formulation Developed by Our Company

1.5.1 Nonclinical data

Standard nude mice models were used to evaluate and compare the *in vivo* anti-tumor efficacy of irinotecan liposome and commercially available irinotecan injection against human pancreatic cancer PANC 0504, human pancreatic cancer BxPC-3, human colon cancer Ls-174t, human colon cancer HCT-116, human ovarian cancer SK-OV-3, and human small cell lung cancer A549. The efficacy of irinotecan liposome in the above tumor models was superior to or equivalent to that of irinotecan injection, especially in the nude mice xenograft models of human pancreatic cancer PANC 0504, human pancreatic cancer BxPC-3, human colon cancer Ls-174t, and human ovarian cancer SK-OV-3, and the toxicities in the nude mice models were not clearly increased. After intravenous injection of irinotecan hydrochloride liposome in tumor-bearing mice at 1 mg/kg and 3 mg/kg, the plasma exposure of non-liposomal parent drug was 7.9% and 56.2% of that in the 10 mg/kg general formulation group, respectively, and the exposure in tumor tissues was 1.0 and 4.5 time(s) that in the general formulation group, respectively. The plasma exposure of metabolite SN-38 was 0.18 and 1.0 time(s) that in the 10 mg/kg general formulation group, respectively, and the exposure in tumor tissues was 0.52 and 2.0 times that in the general formulation group, respectively.

1.5.2 Phase Ia clinical study

The tolerability and PK study of irinotecan liposome in subjects with advanced solid tumors began in Sep. 2014 at the Fudan University Shanghai Cancer Center. Five dose groups were designed, i.e., 60 mg/m^2, 80 mg/m^2, 120 mg/m^2, 160 mg/m^2, and 210 mg/m^2, with 80 mg/m^2 as the starting dose. If 80 mg/m^2 could not be tolerated, the study could be started from the 60 mg/m^2 dose group; if tolerated, studies in other groups could be carried out in the order of ascending doses. One dose was given by intravenous infusion within 90 min on the first day of each 3-week cycle. Efficacy evaluations were conducted once every 2 cycles. This study had enrolled 32 subjects, of whom 3, 12, 14, and 3 subjects were allocated to 80 mg/m^2, 100 mg/m^2, 120 mg/m^2, and 160 mg/m^2 groups, respectively. Among the 3 subjects in the 160 mg/m^2 group, 2 subjects experienced DLTs, which were Grade 3 hyponatremia and Grade 3 diarrhea. The dose escalation had been terminated. The MTD was 120 mg/m^2. It was found in the later 120 mg/m^2 expansion study that this dose had high toxicity, so the MTD was lowered by one level, i.e., 100 mg/m^2. Preliminary efficacy results: Among the 27 subjects who completed the efficacy evaluation, 8 subjects achieved PR, 13 subjects achieved SD, and 6 subjects achieved PD. The main AEs in subjects were myelosuppression, diarrhea, and vomiting. As compared with general irinotecan formulation, the adverse reaction specific to irinotecan liposome was infusion reaction occurring in 9 subjects (28.1%), all of which was Grade 1-2 and could recover by reducing infusion rate or interrupting administration. Among

the 16 SAEs, 2 SAEs of brain metastases with hemiplegia and intestinal obstruction were not related to the treatment, and the others were all related to the treatment, mainly gastrointestinal toxicities (especially 8 events of diarrhea). Preliminary PK results showed that: The total concentration of irinotecan and the plasma exposure of two compounds of metabolite SN-38_LAC (active closed-ring form) increased proportionally with increasing dose. Due to the large inter-individual differences and the limited sample size of individual dose groups, the linear PK conclusion could not be drawn for the time being. In the 120 mg/m^2 group, the PK parameters of liposome developed by our company were comparable to those of Onivyde, a similar drug developed abroad.

1.5.3 Phase Ib clinical study

A single-center, open-label, single-arm, 3 + 3 dose-escalation, phase Ib study was carried out at the Fudan University Shanghai Cancer Center, aiming at evaluating the tolerability, safety, and PK of irinotecan hydrochloride liposome injection in combination with 5-FU/LV in subjects with advanced solid tumors, determining the dose-limiting toxicity (DLT) and maximum tolerated dose (MTD) of irinotecan liposome, and preliminarily observing the efficacy. Four dose groups were designed, i.e., 40 mg/m^2, 60 mg/m^2, 80 mg/m^2, 100 mg/m^2, and 120 mg/m^2, with 60 mg/m^2 as the starting dose. If 60 mg/m^2 could not be tolerated, the study could be started from the 40 mg/m^2 dose group; if tolerated, studies in other groups could be carried out in the order of ascending doses. One dose was given on the first day of each 2-week cycle. Irinotecan liposome was administered by intravenous infusion within 90 min, and 5-FU/LV was administered by intravenous infusion at 2000/200 mg/m^2. Efficacy evaluations were conducted once every 3 cycles.

Enrollment for this study had been completed, and a total of 15 subjects with advanced solid tumors were enrolled, including two subjects with pancreatic cancer. Three subjects were enrolled in the 80 mg/m^2 group, of whom 2 subjects experienced DLTs: One subject experienced Grade 2 neutropenia which delayed the administration for more than 7 days, and the other subject experienced Grade 3 neutropenia with fever. The dose-escalation study was terminated and 60 mg/m^2 was chosen as the MTD. Enrollment for the 60 mg/m^2 group had been completed, with a total of 12 subjects enrolled, of whom 1 subject experienced DLT (Grade 3 diarrhea). The main AEs in subjects were myelosuppression, diarrhea, and vomiting. Most of the AEs were Grade 1-2, and the incidence of Grade 3-4 AEs was relatively low. It preliminarily showed that the dose of 60 mg/m^2 was safe and well tolerated. At present, no infusion reaction occurred, mainly due to the use of premedication with antiemetics and dexamethasone in this study.

The efficacy results showed that in terms of the best overall efficacy, the ORR was 26.7% (4/15), confirmed ORR was 13.3% (2/15), and DCR was 86.7% in the 15 subjects. Subjects with efficacy evaluated as PR: 3 subjects in the 60 mg/m^2 group (1 subject with ampullary tumor has undergone efficacy confirmation, 1 subject with nasopharyngeal carcinoma, 1 subject of breast cancer) and 1 subject in the 80 mg/m^2 group (1 subject with nasopharyngeal carcinoma has undergone efficacy confirmation), indicating promising anti-tumor activity.

The PK data showed that after treatment with irinotecan hydrochloride liposome injection developed by our company or similar drug ONIVYDE at the same dose (120 mg/m^2), the PK parameters including total concentration of irinotecan and maximum concentration C_{max} of SN-38, exposure AUC, half-life $t_{1/2}$, clearance CL, and apparent volume of distribution V_{ss} were similar. The concomitant use of calcium folinate and 5-FU did not reveal any significant effect on the PK of irinotecan.

2. STUDY OBJECTIVES

2.1 Primary Objective

To compare the OS between the two groups.

2.2 Secondary Objective

To compare the following endpoints of experimental group vs. control group:

1) Progression-free survival (PFS);
2) Time to treatment failure (TTF);
3) Objective response rate (ORR);
4) CA19-9 tumor marker response;
5) Quality of life score (EORTC-QTQ-C30);
6) Safety evaluation: adverse events (AEs), including clinical symptoms and signs and laboratory tests, serious adverse events (SAEs).

3. STUDY DESIGN

3.1 Overview of Study Design

This is a randomized, double-blind, single-dummy, parallel-controlled, multicenter clinical study to compare the efficacy and safety of irinotecan hydrochloride liposome in combination with 5-FU/LV vs. placebo in combination with 5-FU/LV as second-line treatment for locally advanced or metastatic pancreatic cancer after treatment failure with gemcitabine-based therapy. Approximately 272 eligible subjects will be randomized in a 1:1 ratio to either the experimental group (irinotecan liposome in combination with 5-FU/LV) or the control group (placebo in combination with 5-FU/LV), and will be randomly stratified according to the following stratification factors:

① Albumin level (≥ 40 g/L vs. < 40 g/L);

② History of fluorouracil therapy (with vs. without);

③ History of gemcitabine therapy (gemcitabine alone vs. gemcitabine combination).

Subjects will be treated according to the randomized dosing regimen until progressive disease (PD) (radiographic or clinical deterioration) or intolerable toxicity occurs. After treatment discontinuation, subjects will undergo a 30-day follow-up period. Afterwards, subjects will be followed up once a month by telephone or other means. Subjects' survival status will be recorded until death or study closure (whichever occurs first). The primary endpoint will be analyzed after approximately 253 deaths occur. The entire study is planned to be completed in 36 months.

In this study, blinded and unblinded steering committees (SCs) are planned to be set up, and an interim analysis will be conducted. After data review, the unblinded SC will provide recommendation on whether to submit application for registration in advance. See SC Charter for detailed information on composition, responsibilities, and procedures of SC.

3.2 Sample Size Estimation

In this study, placebo + 5-FU/LV will be used as the control and a superiority test will be performed between the two groups, with OS as the primary efficacy endpoint for sample size estimation. An interim analysis is planned for this study.

With reference to the original NAPOLI-1 study data for pancreatic cancer (the OS was 6.1 months in the experimental group and 4.2 months in the control group) and the current status of pancreatic cancer in China, the median OS of the experimental group and the control group in this study is set as 5.0 months and 3.5 months, respectively, with significance level $\alpha = 0.025$ (one-sided), a power of 80%, the enrollment duration of 24 months, the total study duration of 36 months, and a dropout

rate of 20%. A design of experimental group:placebo group = 1:1 will be adopted. An interim analysis is planned when 70% of OS events are collected to determine whether to conduct application for registration in advance. Based on the above parameters, at least 253 deaths should be collected and a total of 272 subjects should be enrolled (136 subjects in the experimental group and 136 subjects in the control group) according to the log-rank test for the OS comparison between the two groups and the O'Brien & Fleming type α spending function (EAST 6.5).

3.3 Randomization Factors

Subjects will be allocated by central randomization and enrolled competitively at all sites. The central randomization procedure will be performed using the central randomization system of Nanjing Medical University. After the investigators of each study site participating in this study screen out each eligible subject, they should fill in screening data, obtain randomization numbers, and log into the drug management system and dispense the corresponding study drugs according to randomization numbers.

Subjects will be subjected to stratified randomization based on the following factors at baseline:

1) Albumin level (< 40 g/L vs. ≥ 40 g/L);
2) History of fluorouracil therapy (with vs. without);
3) History of gemcitabine therapy (gemcitabine alone vs. gemcitabine combination).

3.4 Screening Number

After signing the informed consent form (ICF), each subject participating in the study will be assigned an unique 5-digit screening number, which consists of 2-digit study site number and 3-digit subject number (from left to right, site number filled in boxes 1 and 2, and number starting from 001 according to the order of screening at this site filled in boxes 3-5). Once a screening number is assigned to a subject, this screening number cannot be reused. The screening information of the subjects who fail the screening should be recorded in the electronic case report form (eCRF).

3.5 Blinding and Procedures of Blinding Method

The placebo will be provided by the sponsor and should be identical to the investigational drug in appearance. Neither the investigator nor the subjects know the allocation of study treatment. The randomization number is generated by SAS and imported into the central randomization system provided by the Department of Biostatistics, School of Public Health, Nanjing Medical University. The blind code information will be saved as electronic information. When the investigators apply for unblinding, by the consent of the sponsor and the leading site, they can log into the electronic

information system to obtain the blind code information (the information on the assignment of the subjects to the experimental group or the control group).

Unblinded SC will be unblinded at the interim analysis. The interim analysis will be conducted by an unblinded statistician to ensure that the sponsor, investigators, and subjects remain blinded until the primary analysis of the study (253 death events).

4. SELECTION AND WITHDRAWAL OF SUBJECTS

4.1 Diagnostic Criteria

Diagnosed according to the latest version of "CSCO Guidelines for the Diagnosis and Treatment of Pancreatic Cancer"; staged as per American Joint Committee on Cancer (AJCC) Cancer Staging Manual (AJCC 2017, 8th Edition), as shown in the appendix.

4.2 Inclusion Criteria

Subjects meeting all of the following inclusion criteria are eligible to be enrolled in the study:

1. Male or female aged ≥ 18 years old;
2. Pathologically confirmed pancreatic cancer (derived from pancreatic ductal epithelium), also unresectable locally advanced or metastatic pancreatic cancer as shown in clinical records;
3. Treatment failure with gemcitabine-based systemic therapy as first-line treatment for locally advanced or metastatic disease (received at least 1 cycle of gemcitabine-based therapy, and developed PD or intolerance during treatment and PD after the end of treatment) as shown in clinical records, including but not limited to the following gemcitabine-based dosing regimens:
   - Gemcitabine alone;
   - Any gemcitabine-based regimen with or without maintenance treatment with gemcitabine;
   - Gemcitabine alone, followed by combination with platinum, fluorouracil, erlotinib, etc.;
   - Neoadjuvant/adjuvant chemotherapy containing gemcitabine, and relapse within 6 months after the completion of treatment.

   Note: Definition of intolerance: 1) Hematologic toxicities: Grade 3 neutropenia with fever of > 38.5 °C, Grade 3 platelets decreased with hemorrhage symptoms, and other Grade 4 or greater hematologic toxicities; 2) Non-hematologic toxicities: Grade 3 or greater non-hematologic toxicities; 3) The above-mentioned toxicities render the subject unsuitable to continue the original therapy as judged by the investigator.

4. Have measurable or unmeasurable target lesions according to the Response Evaluation Criteria in Solid Tumors (RECIST) V1.1;

5. ECOG performance status of 0-1;

6. Life expectancy ≥ 12 weeks;

7. Adequate organ functions, i.e., meeting the following criteria:

   (1) Hematology test: (have not used hematopoietic growth factors or undergone blood transfusion within the last 7 days)

      a) Neutrophils ≥ 1.5 × 10^9/L;

      b) White blood cells ≥ 3.5 × 10^9/L;

      c) Platelets ≥ 100 × 10^9/L;

      d) Hemoglobin ≥ 90 g/L;

   (2) Biochemical test:

      e) Total bilirubin ≤ 1.5 × ULN (≤ 2.5 × ULN for subjects with biliary tract obstruction after biliary drainage);

      f) Aspartate aminotransferase (AST) and alanine aminotransferase (ALT) ≤ 2.5 × ULN (≤ 5 × ULN for subjects with liver metastases);

      g) Albumin level ≥ 30g/L;

      h) Creatinine clearance ≥ 60 mL/min;

   (3) Cardiac function test:

      i) ECG normal or abnormal (without clinical significance as judged by the investigator)

      j) Left ventricular ejection fraction (LVEF) ≥ lower limit of normal (LLN);

8. Previous surgery, radiotherapy, chemotherapy, or other anti-tumor therapy ended 4 weeks ago or earlier, and general physical conditions or related adverse reactions have recovered (toxicity ≤ Grade 1) or reached a stable state;

9. Participate voluntarily and sign an informed consent form (ICF);

10. Have good compliance and agree to cooperate with survival follow-up.

4.3 Exclusion Criteria

Subjects who meet any of the following criteria are ineligible to participate in this study:

1. Active central nervous system (CNS) metastases (including CNS metastases that are clinically symptomatic, have cerebral edema, and resulted in the use of steroids in the past 28 days or require it, and CNS metastases that have progressed);
2. Subjects with ascites who require clinical intervention (including subjects with moderate to large amount of ascites, such as subjects who should be stable for more than 4 weeks after drainage of ascites);
3. NRS pain score ≥ 4 after standardized treatment with painkillers;
4. Clinically significant gastrointestinal disorders (including hemorrhage, infective inflammation, perforation, obstruction, or Grade > 1 diarrhea);
5. Subjects who developed a second malignant tumor in the past 5 years (except those with cured carcinoma *in situ*, or basal or squamous cell skin cancer; subjects with other tumors that did not recur in the past 5 years can be enrolled);
6. Poorly controlled cardiovascular and cerebrovascular diseases or clinical symptoms, including but not limited to: (1) NYHA Class ≥ III cardiac failure; (2) unstable angina; (3) myocardial infarction or stroke in the past 6 months; (4) supraventricular or ventricular arrhythmia requiring treatment or intervention; (5) hypertension that is difficult to control (systolic blood pressure > 150 mmHg and/or diastolic blood pressure > 90 mmHg after optimal treatment);
7. Subjects with known active hepatitis B (HBsAg positive and HBV DNA ≥ 10^4 copies or ≥ 2000 u/mL);
8. Active infection or unexplained fever of > 38.5 °C in the screening period or on the day of administration that may affect the subject's participation in this study or interfere with efficacy evaluation according to the investigator's judgment (subjects with fever that is judged by the investigator to be caused by tumor can be enrolled);
9. Known allergy to any component of irinotecan liposome or other liposomes, 5-fluorouracil, or calcium folinate;
10. Have participated in clinical trials of other drugs within 4 weeks before the start of the study treatment or did not reach 5 half-lives of the previous investigational drug before study administration, whichever is longer;
11. Pregnant or lactating women;

12. Blood (urine) pregnancy test positive in the screening period for women of childbearing potential (male and female subjects should use reliable contraceptive measures to prevent pregnancy during the study and within 3 months after the last dose);
13. Other medical or social problems that may affect the subject's ability to sign the ICF, the subject's participation in the study, or the interpretation of study results as judged by the investigator.

4.4 Criteria for Subject Withdrawal/Treatment Discontinuation

Treatment should be discontinued for subjects with the following conditions:

1. The subject's disease progressed according to RECIST V1.1;
2. The subject developed intolerable toxicity, or adverse events requiring:
    - the third dose reduction
    - dose delay for more than 21 days from the start of the next cycle, unless the investigator believes that the subject can benefit from the treatment
3. The subject has obviously poor compliance and cannot follow the study protocol;
4. The subject withdraws the ICF and requests to withdraw from the study;
5. In consideration of the subject's rights, safety, and health, the investigator or the sponsor terminates the study or discontinues the treatment for the subject due to any reason in accordance with the GCP guidelines as well as laws and regulations.

4.5 Management of Subjects Experiencing Withdrawal/Treatment Discontinuation

Each participating study site should complete the corresponding examinations at the time of the subject's withdrawal as far as possible according to the protocol, as well as the subsequent follow-up (until death or lost to follow-up). Efficacy (imaging evaluation until PD) and safety evaluation (detailed documentation of AEs and their outcomes) should be included in follow-up. After a subject withdraws from the study, the investigator can recommend or provide new replacement therapy to the subject according to the actual condition, but detailed records must be made.

5. STUDY TREATMENT

5.1 Drug Preparation

5.1.1 Irinotecan liposome

Irinotecan liposome should be prepared by medical professionals with aseptic techniques. The specific principles are as follows:

- Take the required amount of irinotecan liposome drug substance, dilute it with 0.9% sodium chloride solution or 5% dextrose solution to the administration volume of 250 mL, and gently invert the container to mix well the solution;
- Care must be taken to ensure the sterility of the solution in the preparation process. A visual check of the injection for particulate matters and color changes should be done prior to drug administration (this product is a stable off-white suspension);
- Do not use infusion set with filtering function;
- After the infusion is completed, flush the IV line with an appropriate amount of 0.9% sodium chloride solution or 5% dextrose solution;
- The prepared solution for infusion cannot be stored for more than 4 h at room temperature or for more than 24 h at 2-8 °C; the solution for infusion stored at 2-8 °C should be cooled to room temperature before infusion; from the standpoint of microbiology, the product should be freshly prepared before use. If it cannot be used immediately, the user is responsible for ensuring proper storage time and conditions during use;
- The solution for infusion should be protected from light during storage and infusion;
- The drug substance and infusion solution cannot be cryopreserved.

5.1.2 5-Fluorouracil and calcium folinate

The preparation, use, and storage of 5-fluorouracil and calcium folinate must be performed by medical professionals according to the relevant instructions in package inserts and the medication information of 5-fluorouracil and calcium folinate provided by the study site.

5.2 Dosing Regimen

- **Experimental group:**

Irinotecan liposome: 60 mg/m^2, intravenous infusion for at least 90 min, once every 2 weeks. For subjects homozygous for UGT1A1*28/*6 mutation, the starting dose should be reduced by one level (50 mg/m^2), and if no adverse reaction occurs in the first cycle, the dose of subsequent cycles can be elevated to the starting dose level.

LV: 200 mg/m^2, intravenous infusion for 30 ± 10 min, once every 2 weeks.

5-FU: 2000 mg/m^2, intravenous infusion for 46 ± 4 h, once every 2 weeks.

Administration sequence: in the order of irinotecan liposome, LV, and 5-FU.

Premedication: dexamethasone and antiemetics (or use other drugs that can prevent adverse reactions induced by irinotecan and 5-FU/LV according to the hospital's routine practice).

- **Control group:**

Placebo: irinotecan liposome placebo, administered with the same method as that of irinotecan liposome;

LV: 200 mg/m^2, intravenous infusion for 30 ± 10 min, once every 2 weeks.

5-FU: 2000 mg/m^2, intravenous infusion for 46 ± 4 h, once every 2 weeks.

Administration sequence: in the order of placebo, LV, and 5-FU.

Premedication: dexamethasone and antiemetics (or use other drugs that can prevent adverse reactions induced by 5-FU/LV according to the hospital's routine practice).

For the convenience of administration, the protocol allows a deviation of ± 5% between the actual total dose and the theoretical total dose each time.

6. CRITERIA FOR DOSE INTERRUPTION AND MODIFICATION

6.1 General Principles for Modification

To recover from toxicities, the next dose can be delayed for up to 21 days. If the delay exceeds 21 days, the subject should withdraw from the study, unless the investigator and the sponsor consider that the subject can benefit from continued treatment after discussion in terms of the risk-benefit ratio. If the dose is reduced due to toxicities, it is not allowed to resume the original dose in subsequent cycles for the subject. If a third dose reduction is required due to toxicities, the subject should withdraw from the study.

6.2 Dose Modification of Irinotecan Liposome

For the irinotecan liposome + 5-FU/LV experimental group, when the administration of irinotecan liposome needs to be delayed due to toxicities, the administration of 5-FU/LV should also be delayed, and both cannot be administered alone.

After the dose of subjects homozygous for UGT1A1*28 or UGT1A1*6 mutation is elevated to the maximum dose (60 mg/m^2), the dose reduction principle is the same as that for subjects non-homozygous for UGT1A1*28/UGT1A1*6.

6.2.1 Dose modification due to hematologic toxicity

Before the start of administration in each cycle, the subject's bone marrow function should reach:

- Neutrophil count ≥ 1.5 × 10^9/L;
- White blood cell count ≥ 3.5 × 10^9/L;
- Platelet count ≥ 100 × 10^9/L.

If the subject develops hematologic toxicity, the administration of the next cycle can be delayed (see Section 6.1 for the delay time limit) until the subject's bone marrow function recovers to the above level, and the subsequent dose should be modified according to Table 6.1 and Table 6.2. If the subject has febrile neutropenia, the neutrophil count should recover to 1.5 × 10^9/L, and the infection or fever should disappear.

Table 6.1. Dose modification of irinotecan liposome due to neutropenia

**CTCAE Grade of Neutropenia[a]**	**Dose of Irinotecan Liposome in Next Cycle[a]**	
	**UGT1A1*28/UGT1A1*6 Non-mutation homozygote**	**UGT1A1*28/UGT1A1*6 Mutation homozygote[b]**
Grade 1-2<br>(ANC ≥ 1.0 × 10^9/L, ≤ 1.9 × 10^9/L)	Dose not changed	Dose not changed
Grade 3-4<br>(ANC < 1.0 × 10^9/L) or Febrile Neutropenia		
First Onset	Dose reduced to 50 mg/m^2	Dose reduced to 40 mg/m^2
Second Onset	Dose reduced to 40 mg/m^2	Dose reduced to 30 mg/m^2

Notes: a: All dose modifications are based on the most severe toxicity

b: For subjects who are homozygous for UGT1A1*28 or *6 and whose dose has been elevated, the dose modification principle is based on that for non-homozygous subjects

Table 6.2. Dose modification of irinotecan liposome due to other hematologic toxicities

CTCAE Grade of Hematologic Toxicity[a] (except neutropenia)	Dose of Irinotecan Liposome in Next Cycle[a]	
	UGT1A1*28/UGT1A1*6 Non-mutation homozygote[b]	UGT1A1*28/UGT1A1*6 Mutation homozygote[b]
Grade 1-2	Dose not changed	Dose not changed
Grade 3-4		
First Onset	Dose reduced to 50 mg/m^2	Dose reduced to 40 mg/m^2
Second Onset	Dose reduced to 40 mg/m^2	Dose reduced to 30 mg/m^2

Notes: a: All dose modifications are based on the most severe toxicity

b: For subjects who are homozygous for UGT1A1*28 or *6 and whose dose has been elevated, the dose modification principle is based on that for non-homozygous subjects

6.2.2 Dose modification due to non-hematologic toxicity

Grade 3-4 non-hematologic toxicity must recover to Grade 1, baseline level, or stable state before the next cycle of treatment. See Table 6.3 for the principles of irinotecan liposome dose modification due to non-hematologic toxicity. See Section 6.2.3 "Modification due to special adverse reactions" for dose modification in case of diarrhea. Do not modify the dose in case of asthenia or anorexia.

Table 6.3. Dose modification of irinotecan liposome due to non-hematologic toxicity

CTCAE Grade of Non-hematologic Toxicity (except diarrhea, asthenia, and anorexia)[a]	Dose of Irinotecan Liposome in Next Cycle[a]	
	UGT1A1*28/UGT1A1*6 Non-mutation homozygote	UGT1A1*28/UGT1A1*6 Mutation homozygote[b]
Grade 1-2	Dose not changed	Dose not changed
Grade 3-4 (except nausea and vomiting)		
First Onset	Dose reduced to 50 mg/m^2	Dose reduced to 40 mg/m^2
Second Onset	Dose reduced to 40 mg/m^2	Dose reduced to 30 mg/m^2
Grade 3-4 Nausea and/or Vomiting (antiemetic treatment has been given)		
First Onset	Dose reduced to 50 mg/m^2, antiemetic treatment regimen optimized	Dose reduced to 40 mg/m^2, antiemetic treatment regimen optimized
Second Onset	Dose reduced to 40 mg/m^2, antiemetic treatment regimen optimized	Dose reduced to 30 mg/m^2, antiemetic treatment regimen optimized

Notes: a: All dose modifications are based on the most severe toxicity

b: For subjects who are homozygous for UGT1A1*28 or *6 and whose dose has been elevated, the dose modification principle is based on that for non-homozygous subjects

6.2.3 Modification due to special adverse reactions

Actions should be taken for the investigational drug irinotecan liposome in the following special cases.

6.2.3.1 Infusion related reaction

The grade of infusion related reactions is established according to infusion reactions and allergic reactions in CTCAE 4.03, but with slight difference. Relevant measures and suggestions are provided for reference. See Table 6.4 for details.

Table 6.4. Actions to be taken in case of infusion related reaction

Grade of Infusion Related Reaction		Action	Subsequent Administration
Grade 1	Mild flushing or rash, drug-induced fever < 38 °C; no intervention required	● Reduce infusion rate by 50%<br>● Monitor the subject every 15 min to prevent condition aggravation	● Reduce infusion rate (at least 120 min)
Grade 2	Treatment or infusion interruption required, symptomatic treatment required (antihistamines, NSAIDAS, anesthetics, or infusion therapy), rapid onset of action, premedication ≤ 24 h	● Discontinue the treatment<br>● Diphenhydramine hydrochloride 50 mg intravenously, paracetamol 650 mg orally, oxygen therapy<br>● Resume the treatment at 50% infusion rate after symptom remission<br>● Monitor the subject every 15 min to prevent condition aggravation	● Reduce infusion rate (at least 120 min)<br>● Premedication with diphenhydramine hydrochloride 25-50 mg intravenously, dexamethasone 10 mg intravenously, and paracetamol 650 mg orally before study administration.
Grade 3	Bronchospasm symptoms, with or without urticaria; injection medication required; allergy-related edema; hypotension	● Discontinue the treatment, remove the infusion set<br>● Diphenhydramine hydrochloride 50 mg intravenously, dexamethasone 10 mg intravenously, bronchodilators used if bronchospasm occurs, other drugs or oxygen therapy used as needed	● Prohibit dosing of subsequent cycles

Grade of Infusion Related Reaction		Action	Subsequent Administration
Grade 4	Life-threatening consequences; urgent intervention indicated	● Discontinue the treatment, remove the infusion set<br>● Epinephrine administered; bronchodilators or oxygen therapy used if bronchospasm occurs<br>● Diphenhydramine hydrochloride 50 mg intravenously, dexamethasone 10 mg intravenously<br>● Hospitalization considered	● Prohibit dosing of subsequent cycles

6.2.3.2 Diarrhea

See Table 6.5 for irinotecan liposome dose modification due to diarrhea. Diarrhea must recover to Grade ≤ 1 before the next cycle of treatment. When diarrhea causes electrolyte disturbance and prolongs the QTc interval, electrolytes need to be supplemented in time. After both underlying abnormalities and ECG abnormalities recover, the treatment can be continued, the monitoring should be strengthened, and the dose should be modified with caution according to the principles of dose modification in case of diarrhea.

Table 6.5. Dose modification of irinotecan liposome due to diarrhea

CTCAE Grade of Diarrhea[a]	Dose of Irinotecan Liposome in Next Cycle[a]	
	UGT1A1*28/UGT1A1*6 Non-mutation homozygote	UGT1A1*28/UGT1A1*6 Mutation homozygote[b]
Grade 1-2<br>(2-3 more bowel movements than baseline or 4-6 more bowel movements than baseline)	Dose not changed	Dose not changed
Grade 3 (7-9 more bowel movements than baseline) or<br>Grade 4 (> 10 more bowel movements than baseline)		
First Onset	Dose reduced to 50 mg/m^2	Dose reduced to 40 mg/m^2
Second Onset	Dose reduced to 40 mg/m^2	Dose reduced to 30 mg/m^2

Notes: a: All dose modifications are based on the most severe toxicity

b: For subjects who are homozygous for UGT1A1*28 or *6 and whose dose has been elevated, the dose modification principle is based on that for non-homozygous subjects

6.2.3.3 Allergic reaction

Allergic reaction is defined as vascular collapse or shock (systolic blood pressure < 90 mmHg and no response to fluid replacement) with or without respiratory distress occurring within 30 min of study drug infusion induced by allergy. Cutaneous symptoms include pruritus, urticaria, and angioedema. For subjects who develop allergic reaction to the study drugs, the study drugs should be discontinued and active symptomatic treatment should be given.

6.3 Dose Modification of 5-FU and Calcium Folinate

Dose modification of 5-FU and calcium folinate (LV) is adapted to the experimental group and the control group. LV is administered prior to 5-FU. If 5-FU needs to be delayed, LV should also be delayed accordingly. Generally, no modification needs to be made to LV dose (LV dose reduction may be considered in case of LV-related Grade 3-4 adverse reactions, e.g., Grade 3-4 stomatitis).

For the irinotecan liposome + 5-FU/LV experimental group, when any one of irinotecan liposome and 5-FU is delayed, the other should also be delayed, and both cannot be administered alone.

6.3.1 Dose modification due to hematologic toxicity

Before administration in each cycle, the bone marrow function should reach:

- Neutrophil count ≥ 1.5 × 10^9/L;
- White blood cell count ≥ 3.5 × 10^9/L;
- Platelet count ≥ 100 × 10^9/L.

If the subject develops hematologic toxicity, the administration of the next cycle can be delayed (see Section 6.1 for the delay time limit) until the subject's bone marrow function recovers to the above level, and the subsequent dose should be modified according to Table 6.6. If the subject has febrile neutropenia, the neutrophil count should recover to 1.5 × 10^9/L, and the infection or fever should disappear.

Table 6.6. Dose modification of 5-FU due to hematologic toxicity

**Hematologic Toxicity**[a]			**Dose of 5-FU in Next Cycle**[b]
**Neutropenia (× 10^9/L)**		**Platelets Decreased (× 10^9/L)**	
≥1.0	And	≥50	Dose not changed
0.5-<1.0	Or	25-<50	Dose reduced by 25%
< 0.5 or febrile neutropenia	Or	< 25 or platelets decreased with hemorrhage	Dose reduced by 25%

Notes: a: All dose modifications are based on the most severe toxicity; b: If a third dose reduction is required, the subject should withdraw from the study

6.3.2 Dose modification due to non-hematologic toxicity

All Grade 3-4 non-hematologic toxicities must recover to Grade 1, baseline level, or stable state before the next cycle of treatment. No dose modification needs to be made in case of asthenia or anorexia. See Table 6.7 for dose modification due to other non-hematologic toxicities.

Table 6.7. Dose modification of 5-FU due to non-hematologic toxicity

**CTCAE Grade of Non-hematologic Toxicity[a]**		**Dose of 5-FU in Next Cycle[b]**
Grade 1-2 (except asthenia, anorexia, and hand and foot syndrome)		Dose not changed
Grade 3-4 (except asthenia, anorexia, and hand and foot syndrome)		Dose reduced by 25%
Hand and Foot Syndrome	Grade 1	Dose not changed
	Grade 2	Dose reduced by 25%
	Grade 3-4	Treatment discontinuation
Any Grade of Cerebellar Neurotoxicity or Grade ≥ 2 Cardiotoxicity		Treatment discontinuation
Any Grade of Asthenia and Grade 3 Anorexia		Dose not changed

Notes: a: All dose modifications are based on the most severe toxicity

b: If a third dose reduction is required, the subject should withdraw from the study

7. STUDY DRUGS

7.1 Information on Study Drugs

(1) Irinotecan liposome injection

[Generic name] Irinotecan Hydrochloride Liposome Injection

[Chinese Pinyin] Yansuan Yilitikang Zhizhiti Zhusheye

[English name] Irinotecan Hydrochloride Liposome Injection

[Chemical name] (+)-(4S)-4,11-diethyl-4-hydroxy-9-[(4-piperidinylpiperidine)carbonyl]-1H-pyrano[3,4:6,7] indolazine[1,2b]quinoline-3,14-(4H,12H)-dione hydrochloride trihydrate

[Strength] 8 mL: 40 mg

[Appearance] Off-white suspension

[Manufacturer] Jiangsu Hengrui Pharmaceuticals Co., Ltd.

[Shelf life] 24 months

[Storage conditions] 2-8 °C, sealed, protected from light

[Drug packaging] Packaged in vials.

(2) Placebo

[Generic name] Irinotecan Hydrochloride Liposome Placebo Injection

[Chinese Pinyin] Yansuan Yilitikang Zhizhiti Moniji Zhusheye

[English name] Irinotecan Hydrochloride Liposome Placebo Injection

[Chemical name] Non-drug-delivery liposomal system

[Strength] 8 mL: 40 mg

[Appearance] Off-white suspension

[Manufacturer] Jiangsu Hengrui Pharmaceuticals Co., Ltd.

[Shelf life] 24 months

[Storage conditions] 2-8 °C, sealed, protected from light

[Drug packaging] Packaged in vials.

(3) 5-Fluorouracil injection (5-FU)

[Generic name] 5-Fluorouracil Injection

[Chinese Pinyin] Funiaomiding Zhusheye

[English name] 5-Fluorouracil Injection

[Chemical name] 5-fluoro-2,4 (1H,3H)-pyrimidine dione

[Strength] 10 mL: 250 mg

[Appearance] Colorless clear liquid

[Manufacturer] Shanghai Xudong Haipu Pharmaceutical Co., Ltd.

[Shelf life] 24 months

[Storage conditions] Room temperature, sealed, protected from light

[Drug packaging] Packaged in ampules.

(4) Calcium folinate injection (LV)

[Generic name] Calcium Folinate Injection

[Chinese Pinyin] Yayesuangai Zhusheye

[English name] Calcium Folinate Injection

[Chemical name] N-[4-[(2-amino-5-formyl-1,4,5,6,7,8-hexahydro-4-oxo-6-pteridyl)methyl]amino]benzoyl-L-glutamic acid calcium salt pentahydrate

[Strength] 10 mL: 100 mg

[Appearance] Light yellow to yellow clear liquid

[Manufacturer] Jiangsu Hengrui Pharmaceuticals Co., Ltd.

[Shelf life] 12 months (tentative)

[Storage conditions] 2-10 °C, sealed, protected from light

[Drug packaging] Packaged in vials.

7.2 Drug Packaging

- **Irinotecan liposome**

The drugs are packaged in vials, 4 vials per box. The labels of vials and boxes are as follows:

Vial label of irinotecan liposome:

**Irinotecan Hydrochloride Liposome Injection**
Strength: 8 mL:40 mg **Drug No.: _ _ _ _**
Storage: protected from light, sealed, 2-8 °C, do not freezes
Batch No.:
Expiry Date:
Developer: Jiangsu Hengrui Pharmaceuticals Co., Ltd.

Box label of irinotecan liposome:

Drug for the Clinical study of Irinotecan Liposome in the Treatment of Advanced Pancreatic Cancer
**Irinotecan Hydrochloride Liposome Injection**
Clinical Study Approval No.: 2017L04358 **Drug No.: _ _ _ _**
Indication: advanced pancreatic cancer
Strength: 8 mL:40 mg
Quantity: 4 vials
Dose and Administration: 60 mg/m^2, intravenous infusion, once every 2 weeks
Storage: protected from light, sealed, 2-8 °C, do not freeze
Batch No.:
Expiry Date:
Developer: Jiangsu Hengrui Pharmaceuticals Co., Ltd.
Caution: **This drug is only used for clinical study**. Any remaining drug should be returned

- **5-Fluorouracil injection**

The drug is a commercially available product with the original packaging (packaged in ampules, 5 ampules per box) of its manufacturer Shanghai Xudong Haipu Pharmaceutical Co., Ltd.

- **Calcium folinate injection**

The drug is a commercially available product with the original packaging (packaged in vials, 1 vial per box) of its manufacturer Jiangsu Hengrui Pharmaceuticals Co., Ltd.

7.3 Dispensation of Study Drugs

After subjects pass the screening, the investigator will log into the randomization system to obtain randomization numbers, and then log into the drug management system for drug dispensation according to the randomization numbers. One box of drugs (containing four vials of irinotecan liposome or irinotecan liposome placebo) is dispensed each cycle. Before the start of administration to each subject in each cycle, the investigator should log into the drug dispensation system to obtain the drug number, and then give the subject the corresponding drug treatment according to the drug number.

7.4 Management and Storage of Study Drugs

The study drugs will be delivered by a third-party cold-chain logistics company entrusted by the sponsor. The investigator should designate a dedicated person to be responsible for the storage, dispensation, return, inventory, and record of the study drugs. When the study drugs arrive at the study site, the investigator or an authorized person needs to check the storage conditions, count the quantity, verify the drug number, and sign the "Drug Transfer Record". The study drugs should be stored in dedicated cabinet at the study site by designated personnel in strict accordance with the required storage conditions. The study drugs are intended for this clinical study only, and should not be used for any other purposes.

The clinical research associate (CRA) is responsible for monitoring the supply, usage, and storage of the study drugs, as well as the handling of remaining drugs.

7.5 Handling and Return of Study Drugs

7.5.1 Handling of unused/expired drugs

All remaining drugs in the vials should be discarded after use. Try to prevent drugs from releasing in the environment. Drugs should not be disposed of as waste water, and disposal as household garbage should also be avoided. Dispose of drugs with established collection systems, if locally available.

The unopened drugs among those dispensed each cycle should be returned to the sponsor.

Expired drugs should be returned to the sponsor.

7.5.2 Return of study drugs

After the end of the study, the sponsor should collect the remaining drugs (except those retained as samples by the study site) and the used empty vials and packaging for destruction. Relevant return and destruction records should be kept.

8. RULES ON CONCOMITANT MEDICATIONS

8.1 Medications Prohibited or Used with Caution During the Study

8.1.1 Other anti-tumor drugs prohibited

The use of other anti-tumor drugs, including traditional Chinese medicine (TCM), is prohibited during the study (see the appendix for a detailed list).

The following treatments are prohibited in this study:

- Other anti-tumor treatments, including cytotoxic drugs, targeted drugs, endocrine therapy, antibody drugs, and any TCM with tumors as the indication, except irinotecan liposome and 5-fluorouracil specified in this study;
- Radiotherapy with potential efficacy (palliative radiotherapy is allowed to relieve clinical symptoms);
- Immunotherapy and immunosuppressants (except short-term, systemic steroids to control allergic reaction, or treatment for immune-related and injection-related AEs);
- Treatment with other investigational drugs.

8.1.2 Medications used with caution

In the process of concomitant medication, care should be taken to avoid the use of drugs that interact with the investigational drug. These drugs will affect the efficacy and safety of the investigational drug, as detailed below:

CYP3A4 inducers (phenytoin sodium, phenobarbital, carbamazepine), ketoconazole, itraconazole, troleandomycin, erythromycin, diltiazem, verapamil, and Hypericum perforatum (St. John's Wort). The above drugs can interact with irinotecan. Avoid using these drugs and other drugs that may interact with irinotecan. Refer to the package insert of irinotecan. 5-FU may interact with warfarin, so if warfarin has to be used for anticoagulation, coagulation parameters should be closely monitored to avoid any complications caused by drug-drug interactions. For other drug-drug interactions, refer to the package inserts of 5-FU and LV.

8.2 Allowed Concomitant Medications and Treatments

Subjects should actively receive the best supportive care, such as analgesics, antiemetics, antihistamines, anxiolytics, antibiotics, antipyretics, or blood products. Acupuncture therapy is permitted.

Bisphosphonates for bone metastasis is permitted while the subject is receiving study medication. If systemic or local analgesia is not effective in controlling painful lesions of bone metastases, a small area of palliative radiotherapy (the area of the radiotherapy must be < 5% of the bone marrow region, and the percent bone marrow in human skeleton is shown in the figure of the **appendix**) is allowed.

Comorbidities and various AEs should be actively treated. For the drugs used for the treatment of common adverse reactions, see the section (Treatment of Adverse Reactions).

All treatments and medications (including the generic name, dose, route of administration, start time, end time, and indication) used from 30 days prior to signing informed consent form to 30 days after the last dose should be documented in the eCRF in strict accordance with the GCP regulations. The vehicle of the drugs may not be recorded.

9. STUDY PROCEDURES

Subjects must read and sign the informed consent form approved by the ethics committee prior to any procedures of the study. All study procedures must be completed within the time window specified in the study schedule. All relevant examinations (including imaging examinations) of endpoints are carried out in each treatment cycle in accordance with specified time points. For details, see the following observation items and study procedures, or refer to the **"Schedule of Activities"** in this study protocol.

9.1 Observation Items

**Item**	**Requirement and Measurements**
Demographics	Age, gender, ethnicity, weight, and height.
Tumor History	Include pathological diagnosis and clinical diagnosis:<br>(1) Tumor diagnosis: the date of initial diagnosis of the tumor, histological classification, sites of primary lesion and metastases, pathological stage, clinical stage;<br>(2) History of tumor treatment, including surgery, radiotherapy, chemotherapy, targeted therapy, and biotherapy;
Concomitant Diseases	Concomitant diseases and relevant treatment history at baseline (chronic conditions such as diabetes and hypertension);
Concomitant Medication	Drugs used concomitantly within one month.
Medical History	Record subjects' past major diseases, including allergy history and surgery history.
Vital Signs	Blood pressure, pulse, respiratory rate, and body temperature.
Physical Examination	Assess according to organs and systems, including general conditions, skin, oral cavity, eyes, ears, nose, throat, lymph nodes, head and neck, respiratory system, cardiovascular system, abdomen, nervous system, and mental state. Particular attention should be paid to the sites of tumor lesions.

Item	Requirement and Measurements
ECOG PS	According to the ECOG performance status scoring criteria (see the appendix)
NRS Pain Score	According to the NRS pain scoring criteria (see the appendix)
Hematology	White blood cell count (WBC), red blood cell count (RBC), hemoglobin (Hb), platelet count (PLT), neutrophil count (NEU), and lymphocyte count (LYM).
Urinalysis	Urine pH, urine protein, urine glucose, urine ketone body, microscopic urine red blood cells, and microscopic urine white blood cells.
Liver Function	Serum alanine aminotransferase (ALT), aspartate aminotransferase (AST), total protein (TP), albumin (ALB), prealbumin, globulin, total bilirubin (TBil), direct bilirubin (DBil), alkaline phosphatase (ALP), gamma-glutamyl transferase (GGT), lactate dehydrogenase (LDH), and C-reactive protein (CRP).
Kidney Function	Serum creatinine (Cr), blood urea nitrogen (BUN), and creatinine clearance (calculated based on the subject's gender, age, body weight, and blood creatinine; see the appendix).
Electrolytes	Serum potassium ion (K^+), sodium ion (Na^+), chloride ion (Cl^-), calcium ion (Ca^{2+}), magnesium ion (Mg^{2+}), and phosphorus (P)
Blood Lipid and Blood Glucose	Triglyceride (TG), total cholesterol (TC), low-density lipoprotein (LDL), high-density lipoprotein (HDL), and blood glucose (GLU)
Coagulation Function	Prothrombin time (PT), activated partial thromboplastin time (APTT), international normalized ratio (INR), fibrinogen (FIB), thrombin time (TT), and D-dimer
Hepatitis B Virus Test	Hepatitis B surface antigen (HBsAg), hepatitis B surface antibody (HBsAb), hepatitis B e antigen (HBeAg), hepatitis B e antibody (HBeAb), and hepatitis B core antibody (HBcAb); if HBsAg is positive, HBV-DNA load should be quantified
Pregnancy Test	Blood/urine HCG pregnancy test should be performed for women of childbearing potential
ECG	Standard 12-lead ECG
Echocardiography	Left ventricular ejection fraction (LVEF)
Genomics Examination	For UGT1A1*28/UGT1A1*6, centralized testing is adopted. Blood is collected and sent to the same institution for testing and analysis.
Tumor Marker Examination	Carbohydrate antigen 19-9 (CA19-9), carbohydrate antigen 12-5 (CA12-5), and carcinoembryonic antigen (CEA)
Tumor Evaluation	The same imaging evaluation method (CT or MRI) should be adopted throughout the study for the same subject. Tumor evaluation will be performed according to RECIST V1.1. All imaging reports generated during the study should be collected and backed up regularly.

9.2 Screening Period/Baseline

Visit 1: Screening visit (D-14 to D0)

Unless otherwise stated, the following screening procedures must be completed within 14 days prior to randomization:

- **[Signing of informed consent form]**
- **[Demographics]**: age, gender, ethnicity, weight, and height

- **[Tumor history]**:

  Include pathological diagnosis and clinical diagnosis:

  (1) Tumor diagnosis: the date of initial diagnosis of the tumor, histological classification, sites of primary lesion and metastases, pathological stage, clinical stage;

  (2) History of tumor treatment, including surgery, radiotherapy, chemotherapy, targeted therapy, and biotherapy;

- **[Concomitant diseases]**: concomitant diseases and relevant treatment history at baseline (chronic conditions such as diabetes and hypertension);
- **[Concomitant medications]**: drugs used concomitantly within one month;
- **[Medical history]**: record subjects' past major diseases, including allergy history and surgery history;
- **[Imaging examination]**: CT or MRI of the chest and abdomen. A whole body bone scan is required for clinically suspected bone metastasis. Enhanced brain CT or MRI is required for clinically suspected central nervous system metastasis to rule out the possibility. (The CT/MRI scan results obtained before signing the ICF can be used for tumor evaluation in the screening period, as long as the scan is done within 14 days before the start of the study treatment);
- **[Hepatitis B virus test]**
- **[Genomics examination]**

Unless otherwise stated, the following screening procedures must be completed within 7 days prior to randomization:

- **[Weight measurement]**
- **[Vital signs]**, **[Physical examination]**
- **[Hematology]**, **[Blood biochemistry]**
- **[Coagulation function]**, **[Urinalysis]**
- **[Pregnancy test]**
- **[12-Lead ECG]**: If the ECG results are abnormal, additional necessary examinations may be performed as judged by the investigator;

- **[Echocardiography]**
- **[Tumor marker examination]**
- **[ECOG PS]**, **[NRS pain score]**
- **[Quality of life score]**

※ **After subjects complete the above examinations within the specified time limit, check whether they meet the inclusion/exclusion criteria**

Subjects who meet all the [Inclusion Criteria] and none of the [Exclusion Criteria] are **eligible** and need to complete the following procedures:

(1) **Randomization**: Randomization will be performed by the investigator through the online randomization system. The successfully randomized subjects will get the grouping information (experimental group or control group);

(2) **Study medication:** Subjects must begin the assigned study treatment within 48 h after the randomization is completed;

(3) **Record adverse events and concomitant medications**.

9.3 Visits in Treatment Period

9.3.1 Each cycle

Visit 1: each cycle (D1)

Admit subjects into the ward for 2-3 days. The specific time depends on subjects' conditions. Subjects need to complete the following procedures at this visit:

- **[Weight measurement]**
- **[Vital signs]**, **[Physical examination]**
- **[ECOG PS]**, **[NRS pain score]**
- **[Premedication]:** Administer drugs that can prevent adverse reactions according to the medication habits of the study site;
- **[Infusion of study drugs]:** The experimental group is infused with irinotecan liposome + 5-FU/LV and the control group is infused with placebo + 5-FU/LV;
- **Record adverse events and concomitant medications;**

- **After subjects complete the infusion and are discharged from the hospital, dispense the subject diary card and make appointments for the next visit.**

Visit 2: each cycle (D15 ± 2 days)

Subjects return to the study site for visit on D15 (± 2 days) after the start of infusion. Subjects are required to complete the following procedures at this visit:

- **[Weight measurement]**
- **[Vital signs]**, **[Physical examination]**
- **[Hematology]**, **[Blood biochemistry]**
- [**Coagulation function**]
- **[ECOG PS]**, **[NRS pain score]**
- **Record adverse events and concomitant medications;**
- **Return the subject diary card of this cycle.**

Quality of life score

- **Evaluated once every 3 cycles (± 1 week); if the scoring is required on the day of administration, perform it before administration;**

Tumor evaluation

- **[Imaging examination]**: once every 6 weeks (± 1 week), even if there is a dose delay or dose discontinuation.
- **[Tumor marker test]**: once every 6 weeks (± 1 week), even if there is a dose delay or dose discontinuation; the frequency of this test is the same as that of imaging examination.

9.4 End-of-Treatment or Withdrawal Visit

When subjects no longer receive the study treatment due to PD, intolerance, withdrawal of ICF, or other reasons, they should return to the study site for the end-of-treatment or withdrawal visit. Subjects are required to complete the following procedures at this visit:

- **[Weight measurement]**
- **[ECOG PS], [NRS pain score]**
- **[Vital signs]**, **[Physical examination]**

- **[Urinalysis]**, **[ECG]**, **[Pregnancy test]**
- Return **[Subject diary card]**
- Record **[Adverse events]** and **[Concomitant medications]**.

  If the subject does not receive this examination within the last 1 week before the end of treatment/withdrawal, the following examinations should be performed:

- [**Hematology**]
- **[Blood biochemistry] [Coagulation function]**

  If the subject does not receive this examination within the last 4 week before the end of treatment/withdrawal, the following examinations should be performed:

- **[Quality of life score]**
- **[Tumor marker test]**
- **[Imaging examination]**: The technique and method of imaging examination used must be consistent with those used at baseline. The investigator should judge the progression of target lesions and non-target lesions according to imaging examination results and RECIST V1.1.

Note: If the subject does not have PD at this time, follow-up for PD will be conducted subsequently. See Section 9.6 "Visit for Subjects Who Discontinue Treatment/Withdraw for Non-PD Reasons" for details. If the subject has developed PD at this time, follow-up for survival will be conducted subsequently.

9.5 Safety Visit (30 ± 7 days after the end of treatment)

Subjects should return to the study site for safety visit at 30 days (± 7 days) after the last dose of study treatment. Subjects are required to complete the following procedures at this visit:

- **[Weight measurement]**
- **[Vital signs]**, **[Physical examination]**
- **[Hematology]**, **[Blood biochemistry]**, **[Coagulation function]**
- **[Tumor marker examination]**
- **[ECOG PS], [NRS pain score], [Quality of life score]**
- **[Anti-tumor treatment], [Survival status]**

- Record **[Adverse events]** and **[Concomitant medications]**.

9.6 Visit for Subjects Who Discontinue Treatment/Withdraw for Non-PD Reasons

This visit is for subjects who withdraw from study for "non-PD" reasons (intolerable AEs, voluntary withdrawal of ICF, or other reasons without evidence of tumor progression). For tumor progression follow-up, it is recommended to conduct tumor evaluation at the following frequency specified in the study protocol, and fill in [Survival follow-up].

The technique and method of imaging examination used must be consistent with those used at baseline. The investigator should judge the progression of target lesions and non-target lesions according to imaging examination results and RECIST V1.1.

If the subject withdraws from the study due to non-PD reasons, a tumor evaluation should be performed as soon as possible after treatment discontinuation (at least within the 30-day follow-up period), unless a tumor evaluation has been done within 4 weeks before the end of treatment, so as to confirm that no PD occurs and evaluate the overall status of the disease. Afterwards, the tumor evaluation should be performed once every 6 weeks (± 1 week) until PD or start of other anti-tumor treatments.

If the subject is lost to follow-up or withdraws from the study, attempts should be made to contact the subject to get the reason for withdrawal. For the subject who is lost to follow-up, at least one documentary evidence should be provided before determining the loss to follow-up.

All subjects who withdraw from the study should continue to be followed up and observed according to the protocol. The only case where follow-up for endpoint assessment may not be performed is that the subject withdraws the ICF. This ICF withdrawal should be at the subject's sole discretion, which not only means that the subject wants to end the treatment and follow-up, but also means that the investigator is no longer authorized to contact the subject, including any attempt to obtain the subject's survival status.

9.7 Survival Follow-up

Subjects enter the survival follow-up period after completing the 30-day follow-up after the last study dose.

The investigator will contact subjects or their family members by telephone once a month (± 7 days) for survival follow-up to collect the subjects' subsequent anti-tumor treatments and survival information, until death, withdrawal of ICF, lost to follow-up, refusal to receive telephone visit, or end of study.

9.8 Supplementary Instructions for Visit Examinations During the Study

- Additional hematology, blood biochemistry, ECG, etc. may be performed by the investigator as appropriate;
- Subjects should record physical discomfort and medications (including the dose of painkillers, as well as other supportive care and AE treatment) in the subject diary card every day for reference and verification by the investigator, and the investigator will record them in the original medical record and make medical judgment;
- Additional medical examinations may be performed to help diagnosis or treatment when deemed necessary by the investigator. The items and results of these examinations should be recorded in the "Unscheduled Examinations" page of eCRF;
- AE monitoring is recommended to continue until 30 days after the last dose. If AEs still persist, they need to be recorded until recovery to normal or baseline level.

9.9 End of Study

The study ends when the target number of endpoint events is collected and the final analysis is completed.

10. STUDY EVALUATION

10.1 Efficacy Evaluation

10.1.1 Primary efficacy endpoint and observation method

- Overall survival (OS)

OS is defined as the time from randomization to death due to any cause.

For subjects who are still alive at the end of the study, the time of the end of study is used as the censoring date of their survival. For subjects who are lost to follow-up, the time of the last visit is used as the censoring date of their survival.

10.1.2 Secondary efficacy endpoints and observation methods

- Progression-free survival (PFS)

PFS is defined as the time from the date of randomization to the date of documented tumor progression or death due to any cause. The PFS is assessed as per RECIST V1.1. The analysis of this endpoint includes all tumor evaluations during the treatment period and the follow-up period. If the subject has several endpoints that can be evaluated as PD, such as relapse, new lesions, or death, then the first documented endpoint will be used to analyze PFS; if the subject uses other

treatment regimens or anti-tumor treatment against the target lesion, it is also taken as censoring data. Table 10.1 below lists the common progression (or withdrawal) conditions and whether to use as censoring data.

Table 10.1. PFS censoring for common progression or withdrawal

**Condition**	**Date of Progression**	**Result**
No baseline tumor evaluation	The day of randomization	Censoring
Radiographic evidence of unequivocal progression	Date of imaging examination	Progression
Clinical evidence of unequivocal progression	Date of visit or date of evidence discovery	Progression
No progression	Date of last measurable imaging examination	Censoring
Treatment discontinuation due to AEs or other reasons, no evidence of subsequent progression	Date of treatment discontinuation	Censoring
Start of other new anti-tumor treatments	Start date of anti-tumor treatment	Censoring
Death before the first efficacy evaluation	Date of death	Progression
No radiographic evidence of progression at last evaluation, died before next evaluation	Date of death	Progression
Death after missing more than one follow-up visit	Date of death	Progression

- Time to treatment failure (TTF)

TTF is defined as the time from randomization to PD, death, or study discontinuation due to toxicities, including withdrawal of ICF by subjects.

- Objective response rate (ORR)

ORR refers to the proportion of subjects with a best response of CR or PR (through 4-week efficacy confirmation) during the study. Objective response is evaluated based on the Response Evaluation Criteria in Solid Tumors (RECIST) V1.1 (**Appendix**). Subjects must have measurable lesions at baseline. These lesions can be assessed as complete response (CR), partial response (PR), stable disease (SD), and progressive disease (PD) according to RECIST V1.1.

- Change in CA19-9 tumor marker response;

The tumor marker response rate is assessed based on changes in CA19-9 serum concentration. Tumor marker response is defined as at least one decrease in concentration from baseline by at least 50% during treatment. Only subjects with baseline values > 30 U/mL will be included in the evaluation of tumor marker response rates.

- Quality of life score (QoL);

Refer to EORTC QLQ-C30 (version 3). See the appendix for details.

Evaluation method: Observe and score the changes in the clinical symptoms and objective test results of tumor subjects before and after treatment. The quality of life scale is used for evaluation, and the scores of the various items of the scale are recorded in the CRF as per the requirements in the appendix.

10.2 Safety Evaluation

10.2.1 Safety endpoints

- Any spontaneously reported and all directly observed AEs will be evaluated for clinical safety;
- Any abnormal changes in vital signs and physical examinations;
- Abnormalities in laboratory tests and ECG examinations during the study.

10.2.1.1 Adverse events

An adverse event (AE) refers to any untoward medical occurrence in a study subject administered a pharmaceutical product. Any untoward medical occurrence after the subject receives the investigational drug until 30 days after the last dose is judged as an AE, regardless of the causality with the investigational drug. For abnormalities of the physical examination or laboratory tests that are already present during the baseline period, they are also judged as AEs if the severity of these abnormalities is increased after the subjects receive the drug treatment.

AEs are found by asking subjects non-leading questions at each visit during the study. In addition, AEs are also found by subjects' reporting during or between visits, or by physical examinations, laboratory tests, or other evaluations.

The investigator should record in detail any AEs occurring in subjects and evaluate each AE (causality with the investigational drug, whether they are SAEs, etc.). The following information on AEs should be recorded:

(1) Description of all relevant symptoms

(2) Onset time

(3) Severity (graded as per CTCAE V4.03)

(4) Action (e.g., treatment continuation, dose reduction, treatment resumption after interruption, and treatment discontinuation)

(5) Whether they are SAEs

(6) Causality with the investigational drug (related, possibly related, unlikely related, not related, and unassessable)

(7) Duration

(8) Outcome of AEs (resolved, improved, not improved, and aggravated)

Once the subject stops treatment, the toxicities will be evaluated within **30 days after the last dose** if necessary, until all treatment-related toxicities resolve to the baseline level (or Grade ≤ 1 as per CTCAE V4.0), become stable, or are considered irreversible.

10.2.1.2 Criteria for severity assessment of adverse events

The severity of AEs is determined using the National Cancer Institute's Common Terminology Criteria for Adverse Events (NCI CTCAE) V4.03. Refer to the following criteria for AEs not listed in NCI CTCAE V4.03:

Grade 1: (Mild) asymptomatic or mild symptoms; clinical or laboratory test abnormality only; intervention not indicated.

Grade 2: (Moderate) minimal, local, or noninvasive intervention indicated; limiting age-appropriate instrumental activities of daily living (ADL). (Instrumental ADL refers to preparing meals, shopping, using the telephone, managing money, etc.).

Grade 3: (Severe) severe or medically significant but not immediately life-threatening; hospitalization or prolongation of existing hospitalization indicated; disabling; limiting self care ADL. (Self-care ADL refers to bathing, dressing and undressing, feeding self, using the toilet, taking medications, and not bedridden).

Grade 4: (Life-threatening) life-threatening consequences; urgent intervention indicated.

Grade 5: Death related to AE.

10.2.1.3 Judgment of causality between adverse events and study drugs

The judgment of causality between AEs and investigational drug is to determine whether the investigational drug has a reasonable possibility of causing or contributing to the AE through comprehensive evaluation. The judgment factors include whether there is a reasonable time sequence between the onset of AEs and the administration of investigational drug, the characteristics of the investigational drug, the toxicological and pharmacological effects of the investigational drug, the use of concomitant medications, the subject's underlying diseases, medical history, family history, dechallenge, and rechallenge. Generally, facts (evidence) or basis used to judge the causality should be provided.

The investigator should judge the causality between the AE and the investigational drug as "related", "possibly related", "unlikely related", "not related" and "unassessable".

10.2.1.4 Serious adverse events

1) Definition of serious adverse event

A serious adverse event (SAE) refers to a medical occurrence during the clinical trial that results in hospitalization, prolonged hospitalization, disability, incapacity, threats to life or death, or congenital malformation. The following untoward medical events are included:

- Events leading to death;
- Life-threatening events (defined as events where the subject is at immediate risk of death at the time of the onset);
- Events leading to hospitalization or prolonged hospitalization;
- Events leading to permanent or serious disability/incapacity;
- Events leading to congenital anomalies or birth defects.

2) Pregnancy

For pregnancy occurring during the clinical study, the investigator should report the pregnancy to the sponsor within 24 h of knowing the event by filling out the "Hengrui Clinical Study Pregnancy Report/Follow-up Form".

The investigators should track a pregnancy event until its final outcomes (including any premature termination of pregnancy or childbirth), and childbirth should be followed up for 1 month after childbirth. The pregnancy outcomes should be reported to the sponsor. If pregnancy outcomes meet the SAE criteria (such as ectopic pregnancy, spontaneous abortion, intrauterine death, neonatal death, or congenital anomalies, etc.), they must be reported according to SAE procedures.

If a subject experiences any SAE during pregnancy, the SAE should be reported according to the SAE reporting procedure.

The email address for the sponsor to receive the report of pregnancy is:

hengrui_drug_safety@hrglobe.cn

3) Progressive disease

PD (including progressive signs and symptoms) is not necessarily reported as an SAE. However, death due to PD during the study or safety reporting period should be reported as an SAE. Hospitalizations due to signs and symptoms of PD should not be reported as an SAE. If a subject dies during administration or within 30 days after the last dose, the event leading to the death must be reported as an SAE.

4) Other anti-tumor treatments

If a subject is to start another anti-tumor treatment, AEs except death will be reported until 30 days after the last dose of the study drugs. Death that occurs within the SAE reporting period after the end of study treatment should be reported regardless of whether the subjects receive other treatments.

5) Hospitalization

During the study, AEs that lead to hospitalization or prolonged hospitalization should be considered as SAEs. Any initial hospital admission by a medical facility meets this criterion (even if less than 24 hours).

Hospitalization does not include the following:

- Hospitalization at a rehabilitation institution
- Hospitalization at a sanatorium
- General emergency admission
- Day surgery (e.g., outpatient/same-day/ambulatory surgery)

Hospitalization or prolonged hospitalization not related to the adverse events is not considered an SAE. For example:

- Hospitalization due to pre-existing disease and unrelated to an AE, without aggravation of the pre-existing diseases (e.g., hospitalization to examine laboratory abnormalities that have persisted before the study until now);
- Hospitalization for management reasons (e.g., annual physical examination);
- Hospitalization during the study as specified in the study protocol (e.g., as required by the protocol);
- Elective hospitalization unrelated to an AE (e.g., elective cosmetic surgery);

- Scheduled treatment or surgery before the start of study unrelated to an AE that should be documented throughout the entire study protocol and/or in the subjects' individual baseline information;
- Hospitalization unrelated to an AE but merely for use of blood products.

Diagnostic or therapeutic invasive (e.g., surgery) or non-invasive procedures should not be reported as AEs. However, the disease condition leading to such procedures should be reported if it meets the definition of an AE. For example, acute appendicitis during the AE reporting period should be reported as an AE and the appendectomy should be documented as the treatment method for the event.

10.2.1.5 Reporting of serious adverse events

SAEs that occur starting from the signing of the informed consent form until 30 days (inclusive) after the last dose should be reported. During this period, in the event of an SAE, whether it is an initial or follow-up report, the investigator must complete, sign, and date the "New Drug Clinical Study Serious Adverse Event (SAE) Report Form" immediately within 24 h of awareness, notify the sponsor within 24 h of knowing of the SAE, and submit the report to relevant authorities in time in accordance with regulatory requirements. If the investigator is aware of any SAE that occurs 30 days after the last dose, the investigator should report the event suspected to be related to the investigational drug to the sponsor immediately.

The email address for the sponsor to receive the report of SAE is:

hengrui_drug_safety@hrglobe.cn

The symptoms, severity, causality, time of onset, duration of treatment, actions taken, time and method of follow-up, and outcomes should be documented in detail in the SAE report. If the investigator believes that an SAE is not related to the investigational drug but potentially related to the study conditions (e.g., termination of past treatment or comorbidities during the study), then this causality should be detailed in the description section of the "New Drug Clinical Study Serious Adverse Event (SAE) Report Form". If the severity of an SAE or its causality with the investigational drug changes, an SAE follow-up report should be submitted to the sponsor immediately. All SAEs should be followed until resolved or stabilized. If an error is found in a previously reported SAE, such SAE may be revised, revoked, or downgraded in follow-up reports and reported in accordance with the SAE reporting procedure.

10.2.1.6 Suspected unexpected serious adverse reaction (SUSAR)

The suspected unexpected serious adverse reaction (SUSAR) refers to any serious adverse reaction that is suspected and unexpected, and whose nature and severity of clinical manifestations exceed the existing information such as the Investigator's Brochure of the investigational drug, the package insert of the marketed drug, or the summary of product characteristics. The sponsor should immediately report SUSARs to regulatory authorities, investigators participating in the clinical study, and relevant institutions according to regulatory requirements. After receiving the SUSAR report, the investigator should timely receive and read it and report the event to the ethics committee.

10.2.2 Emergency unblinding

In cases where unblinding is necessary during the study, such as SAE occurrence, the responsible investigator at the study site should submit the application, and the medical director of the sponsor and the principal investigator should make a joint decision on whether to unblind. The blind code information is saved as electronic information and kept by dedicated technicians from the Department of Epidemiology and Health Statistics of Nanjing Medical University. If unblinding is necessary, the sponsor's project manager or CRA of each site should be notified and the dedicated technicians should be contacted to obtain the blind code information (the information on the assignment of the subjects to the experimental group or the control group) by logging into the electronic information system within 24 h. Once unblinded, the subject should discontinue the study treatment but continue to be observed until tumor progression and death.

10.2.3 Treatment of common adverse reactions

Different from irinotecan, irinotecan liposome is encapsulated inside the liposome, so it may lead to toxicities that have not been observed in patients taking general irinotecan formulation. All subjects should be closely monitored for vital signs and toxicity symptoms, especially at the first administration.

10.2.3.1 Infusion reaction

After administration of liposomal drugs, a small number of subjects may experience acute infusion reactions, with related symptoms including flushing, tachypnea, facial swelling, headache, chills, back pain, chest tightness, and hypotension. In most subjects, the above symptoms will generally resolve within 24 h after drug discontinuation. In some subjects, the above reactions will resolve by slowing down the infusion rate. Generally, most subjects who have experienced liposomal drug-induced infusion reactions can tolerate relevant complications at subsequent administration. According to the severity of infusion reactions, the subject is given diphenhydramine hydrochloride 50 mg intravenously, dexamethasone 10 mg intravenously, and paracetamol 650 mg

orally. Bronchodilators can be used if bronchospasm occurs, and other drugs or oxygen therapy may be used as needed. For specific treatment, refer to the section on dose modification.

10.2.3.2 Diarrhea

Irinotecan can induce both acute and delayed diarrhea, which can be treated in different ways. Acute diarrhea is a cholinomimetic symptom (occurring during administration or within 24 h after administration). It is usually transient and rarely severe. Concomitant symptoms include rhinitis, salivation, miosis, lacrimation, sweating, flushing, and abdominal colic caused by intestinal obstruction. For subjects with acute cholinergic symptoms during previous treatment, atropine can be given prophylactically prior to the next dose according to the investigator's opinion.

Delayed diarrhea (occurring after 24 h post-dose) is often life-threatening because its long duration of action may lead to dehydration, electrolyte disturbance, and even sepsis. Loperamide should be given immediately once delayed diarrhea occurs, and somatostatin should be considered if diarrhea persists. The synthetic somatostatin (octreotide) is administered by intravenous infusion or subcutaneous injection with gradual dose increase. The dose of octreotide ranges from 100 µg twice a day to 500 µg three times a day, and the maximum tolerated dose is 2000 µg three times a day, with 5 days as a cycle. Persistent severe diarrhea will cause the body to lose large amounts of water and electrolytes, which may lead to life-threatening dehydration, kidney failure, electrolyte disturbance, and even cardiovascular disorder. Infection complications increase and chemotherapy causes neutropenia, which thus increase the risk of sepsis. Subjects who develop diarrhea should be closely monitored. Water and electrolytes should be supplemented in case of dehydration, and antibiotics should be given in case of intestinal obstruction, fever, or severe neutropenia. For specific treatment measures, refer to the ASCO guidelines for the treatment of chemotherapy-induced diarrhea, as shown in Table 10.2 below.

Table 10.2. ASCO guidelines for the treatment of chemotherapy-induced diarrhea

Clinical Manifestation	Intervention
Diarrhea, any grade	Oral loperamide (2 mg every 2 h for irinotecan-induced diarrhea; 2 mg every 4 h for 5-FU-induced diarrhea):<br>Continue dosing until 12 h after diarrhea stops
Diarrhea persists after continuous administration of loperamide for > 24 h	Oral fluoroquinolone antibiotics × 7 days
Diarrhea persists after continuous administration of loperamide for > 48 h	Loperamide discontinuation; hospitalization; intravenous fluids
Neutrophil count < 500/µL, regardless of diarrhea or fever	Oral fluoroquinolone antibiotics (continue dosing until neutropenia resolves)
Persistent diarrhea with fever, even with normal neutrophil count	Oral fluoroquinolone antibiotics (continue dosing until fever or diarrhea resolves)

10.2.3.3 Neutropenia

Deaths from sepsis due to severe neutropenia once occurred in subjects receiving irinotecan. Complications resulting from neutropenia need to be actively treated with antibiotics.

Subjects with neutropenia or neutropenia with fever should be given granulocyte colony-stimulating factor. Only when the subject has developed Grade 3-4 neutropenia or neutropenia with fever at least once, or there is evidence that Grade 3-4 neutropenia or neutropenia with fever occurred in past anti-cancer treatment, the subject can be given granulocyte colony-stimulating factor prophylactically.

10.2.3.4 Nausea and vomiting

All subjects will be given dexamethasone and 5-HT_3 antagonists (ondansetron and granisetron recommended) prophylactically, except those with contraindications. Subjects who develop vomiting with delayed diarrhea should be hospitalized as soon as possible.

10.2.3.5 Intestinal obstruction

Subjects with intestinal obstruction should be treated with antibiotics in time. Before the symptoms of intestinal obstruction disappear, the subjects cannot receive irinotecan liposome.

10.2.3.6 Hepatic function abnormal

The clearance of irinotecan hydrochloride decreases in subjects with hyperbilirubinemia, so the risk of hematologic toxicity increases. When the bilirubin level is 1.0-2 times the upper limit of normal (ULN), the incidence of Grade 3/4 neutropenia is higher than that in subjects with normal bilirubin level (50% vs. 18%), and close monitoring should be performed. Liver protecting therapy should be given when the subject develops hepatic impairment.

Subjects with abnormalities in the glucuronidation of bilirubin, such as those with Gilbert's syndrome, are at a high risk of myelosuppression following treatment with this product.

10.2.3.7 Drug overflow

Prevent from drug overflow, and monitor the injection site in case inflammation occurs. If drug overflows, flush with sterile normal saline and compress ice at the site of overflow.

11. INFORMED CONSENT AND ETHICAL STANDARDS

This clinical study must comply with the "Declaration of Helsinki (1996)", "Good Clinical Practice (GCP)" issued by the China Food and Drug Administration (CFDA, now NMPA), and relevant regulations. The study can only be initiated after obtaining the approval from the ethics committee of the leading site. During the study, any revision to this protocol must be reported to the ethics committee and put on record.

The clinical investigator must follow all applicable laws and regulations to protect the subjects. The ICF used in the informed consent process must be reviewed by the sponsor, approved by the institutional review board (IRB)/institutional ethic committee (IEC), and available for inspection.

The clinical investigator must state to study subjects that all subjects participate in the clinical study voluntarily and have the right to withdraw from the study without being discriminated and retaliated against with their medical treatment and right unaffected, and continue to receive other therapies. All subjects are acknowledged that the participation of the study and their personal information during the study will be kept confidential. Also, the subjects are informed of the nature, objectives, expected potential benefits, and possible risks and inconvenience of the clinical study, other alternative treatment options, and rights and obligations of the subjects in accordance with the "Declaration of Helsinki". Subjects are given sufficient time to consider whether to participate in the study and sign the informed consent form.

Before the implementation of any process required by the study protocol, the subjects must:

- Be informed of information relevant to the study and all content and terms in the informed consent form.
- Be given sufficient time to ask questions and consider whether to participate the study.
- Be enrolled in the study voluntarily.
- Sign and date the informed consent form approved by the IRB/IEC.

In the case of major changes during the study, the protocol should be amended. Unless it is necessary to eliminate obvious direct harms to subjects, the investigator must not make any change to the study without the approval of the IRB/IEC and sponsor. Changes to the study protocol intended to eliminate obvious direct risks to subjects can be implemented immediately, but the change must be recorded in protocol amendments, reported to the IRB/IEC, and submitted to relevant regulatory authorities within a required period. The process of protocol amendment must follow the same process of review and approval of the original protocol.

12. DATA MANAGEMENT

12.1 Case Report Form and Electronic Data Capture

Data management is to ensure the reliability, integrity and accuracy of the data, with an objective to obtain authentic data of high quality for statistical analysis.

The term case report form (CRF) used in this protocol refers to the clinical data of subjects collected in accordance with the requirements of the study protocol. According to the data capture method of this study protocol, the CRF can be a paper record of data, an electronic record of data, or both.

The CRF of each subject participating in the clinical study should be completely filled out. Hengrui is the sole owner of all completed original CRFs. Aside from Hengrui or the representatives from regulatory authorities, no one is allowed to provide any data to a third party without Hengrui's written permission. The investigator is ultimately responsible for the collection and reporting of all clinical, safety, and laboratory data recorded in the CRF and other data collection forms (source records) to ensure the attributability, readability, timeliness, originality, accuracy, durability, integrity, and consistency of the record.

The CRF must be signed by the investigator or authorized personnel to confirm the truthfulness of the data recorded. Any data correction made in the CRF and source records must be dated, signed, and provided with necessary explanations, and the source records must not be covered.

Source records are usually graphs and medical history records from a hospital or a physician, and the data collected in the CRF must be consistent with the data in these graphs and medical history records. In some cases, CRF can also be used as the source record. In such cases, the study site should have relevant documentation to clarify which data will be recorded in the CRF, and use the CRF as the source record.

12.2 Study Record Keeping

In order to meet the requirements of the regulatory authority or Hengrui on review and/or audit, the investigator/study site must agree to maintain relevant records, including the identification number of all participating subjects (sufficient information to be linked to the records, such as CRF and hospital records), all original copies of signed informed consent forms, all CRF copies, safety report forms, source records, details of treatment, and relevant communication documents (e.g., mails, meeting minutes, telephone reports). The investigator/study site should keep the records according to related specifications.

If the study record cannot be kept for any reasons, the investigator/study site should inform Hengrui in advance. The study records must be transferred to a recipient designated by Hengrui, such as another investigator, another institution, or an independent third party arranged by Hengrui. Study documents should be kept by the study site for 5 years after completion of the clinical study and by the sponsor until 5 years after market approval of the product. Even after the retention period has expired, the investigator must still obtain written permission from Hengrui before disposing any records of this study. Hengrui will inform the investigator/study site if the documents are no longer required to be saved.

12.3 Data Management Procedures

A special database is established for this study with the EDC system used by the Data Management Department of Jiangsu Hengrui Pharmaceuticals Co., Ltd. After data entry by the CRC or investigator of each site, the CRA, medical manager, and data administrator will conduct data verification and raise queries online for questionable data in the EDC. The investigator should timely confirm and modify the data after checking the data against source records, until the queries are solved and closed.

12.4 Data Locking and Unblinding

After the completion of the study, data will be processed by the data statistical institution authorized by the sponsor. Inconsistency in the documents will be discovered via data verification. Any inconsistency in the documents should be clarified by contacting investigator and CRA. Database will be locked after the data is verified. No access is allowed without authorization. This study will adopt the double unblinding method. After review of blinding, the data are locked, and the staff of the study site who keep blind codes will perform unblinding for the first time, i.e., inform the biostatistician of the group (A or B) corresponding to subject number for statistical analysis of all data. When the statistical analysis is finished and the final report is completed, perform second unblinding at the clinical summary meeting to announce the exact groups of groups A and B.

13. STATISTICAL ANALYSIS METHODS AND DATA ANALYSIS

13.1 Definition of Analysis Sets

- Full analysis set (FAS): According to the intention-to-treat (ITT) principle, all subjects who have signed the ICF and have been randomized, regardless of whether they have taken drugs, constitute the FAS of this study. The division of this population is based on the number of randomized subjects from the randomization system. This dataset is mainly used for the analysis of efficacy endpoints.

- Safety set (SS): SS, a subset of FAS, includes subjects who have received at least one dose of treatment. This set is used for safety analysis. All analyses conducted in this set are based on the actual treatment intensity.
- Per-protocol set (PPS): PPS is a subset of FAS. All subjects who meet the protocol-specified criteria, have good compliance, and have received the study treatment for at least 6 weeks constitute the PPS of this study.
- Evaluable patient for tumor response (EP): Refer to all patients who have been randomized and received treatment, meet the inclusion/exclusion criteria of the study protocol, have measurable target lesions, and have undergone tumor evaluation at least once during treatment, except for patients with early PD, such as symptom aggravation and tumor-induced death.
- Evaluable patient for tumor markers: Refer to patients with CA19-9 level > 30 U/mL at baseline.

13.2 Statistical Analysis

All statistical analyses will be performed using SAS 9.4. In addition to the primary endpoint, all statistical tests will adopt 2-sided tests. $P \leq 0.05$ is considered statistically significant, and 95% confidence is used for confidence interval. According to the O'Brien & Fleming type α spending function, the final analysis of primary endpoint will be conducted using 95.4% confidence. The corresponding P value less than 0.046 (two-sided) will be considered statistically significant.

A statistical analysis will be conducted on administration in this study to calculate drug exposure throughout the study. The baseline data will be analyzed based on the FAS and all efficacy endpoints will be analyzed based on the FAS and PPS according to the randomized group. The safety analysis will be carried out based on the SS according to the actual group. The continuous variables obtained during various visits will be statistically described by mean ± standard deviation or median (minimum, maximum). The categorical variables obtained from treatment groups during various visits will be statistically described by frequency (proportions).

13.2.1 Efficacy analysis

A. Analysis of overall survival

The primary endpoint of the study is OS. OS is defined as the time from randomization to death due to any cause. For subjects who are still alive at the end of the study, the time of the end of study is used as the censoring date of their survival. For subjects who are lost to follow-up, the time of the last visit is used as the censoring date of their survival.

For the primary efficacy endpoint OS, the final analysis will be performed when 253 deaths (≥ 80% of subjects die) are collected in this study. In the analysis of the primary efficacy endpoint, the survival functions of the experimental group and the control group will be estimated by the Kaplan-Meier method, and the survival curve will be plotted. The survival functions of the two groups will be compared by the unstratified log-rank test. In addition, if the proportional hazard assumption is established between the two groups, a Cox model can be used to estimate the hazard ratio (HR) between the two groups and its overall 95% confidence interval (CI) will be calculated.

For the secondary analysis of the primary efficacy endpoint OS, the hazard ratio (HR) and its 95% CI will be estimated, taking into account important covariates such as albumin level (< 40 g/L vs. ≥ 40 g/L), history of fluorouracil therapy (with vs. without), and history of gemcitabine therapy (gemcitabine alone vs. gemcitabine combination).

In addition, a sensitivity analysis will be conducted on the survival of the FAS population with the following methods to assess the reliability of the results of primary analysis:

- Log-rank test on efficacy in the PPS population;
- Log-rank test stratified for efficacy (stratified by randomization stratification factors);
- HR between the two groups is estimated using a Cox proportional hazards model, and HR and its 95% CI are estimated by considering important covariates such as randomization stratification factors;
- Log-rank test on the survival of subjects with subsequent anti-tumor treatment as censoring data;
- Univariate analysis of potential independent prognostic factors through Cox regression;
- Stratified test on efficacy in different populations;

Prognostic factors include: baseline ECOG, baseline albumin, disease stage, site of primary tumor, number of times of prior chemotherapy, radiotherapy, and surgery, time of the last prior treatment, best response to prior treatment, baseline CA19-9, gender, and age.

B. Progression-free survival (PFS)

PFS is defined as the time from the date of randomization to the date of documented tumor progression or death due to any cause. The PFS is assessed as per RECIST V1.1. The analysis of this endpoint includes all tumor evaluations during the treatment period and the follow-up period.

The PFS of the two groups will be compared using the log-rank test. The PFS curves will be plotted using the Kaplan-Meier method. HR and its 95% CI will be estimated using a Cox proportional hazards model. In addition, stratification analysis will be performed based on randomization stratification factors. This study will explore the impact of analyses that consider stratification and use different prognostic factors as covariates on PFS. In addition, different censoring and alteration methods for missing data can be used for the sensitivity analysis of PFS. The method of sensitivity analysis will be completely specified in the statistical analysis plan.

C. Time to treatment failure (TTF)

TTF is defined as the time from randomization to PD, death, or study discontinuation due to toxicities, including withdrawal of ICF by subjects. The Kaplan-Meier method described in the analysis of PFS will be used to analyze TTF.

D. Objective response rate (ORR)

ORR will be assessed as per RECIST V1.1.

ORR refers to the proportion of subjects with a best response of CR or PR (through 4-week efficacy confirmation) during the study. The number and proportion of subjects with confirmed objective response as well as 95% CI should be calculated, and the ORR between the two groups should be compared using Fisher's exact test. This part of the analysis is applicable to the EP population.

If the sponsor requires an independent radiological evaluation in support of a new drug application or for other reasons, the response of all subjects may be evaluated by an independent committee of clinicians, the sponsor, or other qualified teams. If the evaluation results of the independent professional team and the study party are inconsistent, priority should be given to the evaluation results of the independent committee.

E. Analysis of tumor markers

Serum CA19-9 will be determined within 7 days before administration, and every 6 weeks thereafter. The tumor marker response rate is assessed based on changes in CA19-9 serum concentration. Tumor marker response is defined as at least one decrease in concentration from baseline by at least 50% during treatment. Only subjects with baseline values > 30 U/mL will be included in the evaluation of tumor marker response rates. This part of the analysis is applicable to the set of evaluable patients for tumor markers.

F. Quality of life score (QoL)

The evaluation will be performed with EORTC-QLQ-C30 questionnaire to compare the difference in score between the two groups.

13.2.2 Safety analysis

With treatment groups and subjects as the analysis objects, a statistical analysis will be conducted on treatment-related adverse events (TRAEs) based on the grading criteria by system organ class (SOC) in NCI CTCAE V4.03 and MedDRA. Overall AEs, SAEs, TRAEs, and Grade 3-4 AEs will be listed separately. Laboratory results of each treatment group at each visit will be listed separately. Abnormal laboratory test results will be evaluated according to NCI CTCAE. The incidence and severity of AEs and SAEs in various groups may be compared using Fisher's exact test when necessary.

The safety data of each treatment group in every week of every cycle will be analyzed and summarized. Overall safety will be assessed according to cross-cycle CTCAE grade, SOC, and exposure. The analysis of safety data involves the dose and drug exposure in treated subjects.

13.2.3 Interim analysis

One interim analysis is planned in this study. When the number of endpoint events reaches 70% (177), the unblinded SC will conduct the efficacy and safety evaluation on the unblinded data and provide recommendation on whether to submit application for registration in advance. The superiority boundaries determined using the O'Brien & Fleming type α spending function for the interim analysis are as follows:

Table 13.1. Superiority boundaries in the interim analysis and final analysis

Time Point	Number of Endpoint (OS) Events	Superiority Boundary Z-Value (Corresponding HR)	One-Sided Nominal Significance Level of Superiority
Interim Analysis	177 (70%)	-2.438 (HR=0.692)	0.007
Final Analysis	253	-2.000 (HR=0.777)	0.023

Note: OS = Overall Survival;

The interim analyses will be completed by unblinded statisticians and their programming team. The interim analysis results will be reviewed by the unblinded SC, and the SC will determine whether to put forward the proposal of application for registration in advance to the sponsor according to the results. If the sponsor applies for registration in advance, the early unblinding will be conducted at the same time (see 12.4 for details).

14. STUDY MANAGEMENT

14.1 Study Management Structure

This is a multicenter clinical study in China. Clinical monitoring and audit will be conducted by Jiangsu Hengrui Pharmaceuticals Co., Ltd. Statistical analysis will be conducted by the Department of Biostatistics, School of Public Health, Nanjing Medical University.

14.2 Standard Operations

The sponsor must comply with the GCP and Provisions for Drug Registration. The investigational drugs must be produced in a workshop that meets the Good Manufacturing Practice (GMP) and have undergone strict quality inspections. Laboratory tests are performed by each study site in accordance with standard operating procedures (SOP). The laboratory test methods and quality control of each study site should be consistent. The clinical laboratory of each study site should perform internal quality control in accordance with regulations and obtain the clinical laboratory quality assessment certificate from the National Center for Clinical Laboratories.

14.3 Training

According to the GCP, the qualifications of the CRA must be approved by the sponsor. The study site director should provide protocol training to the investigator prior to the start of the study. The investigator must have read and understood the contents of the clinical study protocol, be familiar with the GCP, standardize the method of documentation and the assessment criteria, and follow the protocol strictly.

14.4 Clinical Monitoring

The CRA must follow the GCP and SOP, make visits to the study site for clinical monitoring on a regular basis or according to the actual conditions, supervise the implementation and progress of the clinical study, check and confirm that all data recorded and entered into CRF are correct and intact and are consistent with raw data, and ensure that the clinical study is implemented following the study protocol. The investigators should cooperate with the CRA actively. Specifically, the CRA is responsible for:

1. Confirming that the study site is qualified prior to starting the study, including personnel and training, a well-equipped and functional laboratory with various study-related test conditions, sufficient number of subjects, and study personnel's familiarity with the protocol requirements;

2. Monitoring how the investigator is implementing the study protocol during the course of the study, confirming that informed consent forms are obtained from all subjects before the study, the enrollment rate and progress of the study, as well as the eligibility of enrolled subjects;
3. Confirming the accuracy and integrity of documentations and reports, and ensuring accurate data entry of all case report forms and consistency with raw data. All errors or omissions have been corrected or noted, signed and dated by the investigators. Dose modifications, treatment changes, concomitant medications, intercurrent diseases, lost to follow-up, and missed examinations should be confirmed and documented for each subject. Verifying that subject's withdrawal and lost to follow-up are explained in the case report form;
4. Confirming that all AEs have been recorded, and that SAEs have been recorded and reported within the specified time frame; verifying that the investigational drugs are supplied, stored, dispensed, and returned in accordance with relevant regulations, and corresponding documentation are made;
5. Recording clearly and faithfully visits, tests, and examinations that the investigator has failed to perform, and whether errors or omissions have been corrected;
6. Completing a written monitoring report after each visit, which should state the date and time of the monitoring visit, the name of the CRA, and the findings of the visit.

14.5 Audit

The Quality Assurance Department of the sponsor may conduct audit on the study in the clinical research institution. The audit covers the supply of drugs, required study documents, documentation of the informed consent process, and consistency between case report forms and original documents. The content and scope of the audit may also be expanded accordingly. The investigators must agree to participate in the audit at a reasonable time and in a reasonable manner.

14.6 Quality Control and Quality Assurance

To ensure the quality of the study, the sponsor and the investigator will jointly discuss and formulate a clinical study plan before the formal study initiation. All study personnel participating in the study will receive GCP training.

All study sites must follow the SOPs for the management of study drugs, including their receipt, storage, dispensation, and return.

According to the GCP guidelines, necessary measures must be taken at the design and implementation phases of the study to ensure that all collected data are accurate, consistent, intact, and reliable. All observed results and abnormal findings in the clinical study must be verified and recorded in a timely and serious manner to ensure data reliability. All instruments, equipment, reagents, and standards used in various tests in the clinical study must have stringent specifications and be operated under normal conditions.

The investigator will input data required by the protocol into the CRF. The CRA will check whether the CRF is completely and accurately filled out and guide the study site personnel for necessary correction and addition.

The drug regulatory authorities and the sponsor may entrust auditors to carry out systemic inspection of study-related activities and documents to assess whether the study is implemented in compliance with the study protocol, SOPs, and relevant regulations and whether the study data are recorded in a prompt, truthful, accurate, and intact manner. The audit should be performed by personnel not directly involved in this clinical study.

14.7 Study Termination

14.7.1 Criteria for terminating the study at sub-sites

The sponsor has the right to terminate the study at a study site if it is found that the investigator of the site is seriously or persistently non-compliant to the protocol and other study procedures, which may interfere with the proper implementation of the study. In such case, the sponsor will immediately inform the investigator and the regulatory authorities of the termination.

If the investigator terminates or suspends the study at a study site, the subjects must be informed immediately, and this decision and the reasons for termination must be reported in writing to the study site and regulatory authorities, as required by regulations. The investigator must also report to the study site and sponsors as soon as possible, stating reasons.

14.7.2 Criteria for terminating the entire study

This study may be prematurely terminated or suspended if there are valid reasons to do so. If the study is prematurely terminated or suspended, the sponsor must submit a written notice to the investigator, NMPA, and relevant authorities, stating the corresponding reasons. The principal investigator must immediately notify the ethics committee and provide relevant reasons.

The termination criteria of this study include but are not limited to the following:

- Discovery of unexpected, significant, or unacceptable risks to the subjects;
- Major errors in the protocol found during the implementation of the study;

- Ineffective study drug/treatment, or meaninglessness to continue the study;
- Seemingly extreme difficulties in completing the study due to reasons such as severe delays in subject recruitment or frequent protocol deviations.

15. DISCUSSION, APPROVAL, AND AMENDMENT OF STUDY PROTOCOL

This clinical study protocol should be formulated after discussion by the principal investigators of participating sites, approved by Jiangsu Hengrui Pharmaceuticals Co., Ltd., and implemented upon approval by the ethics committees of participating sites. During the clinical study, any revisions to the protocol should be reported to the ethical committee for approval or filing.

16. PUBLICATION OF STUDY RESULTS

The study results are the property of Jiangsu Hengrui Pharmaceuticals Co., Ltd. Hengrui does not restrict the publication of any information collected or generated by the investigators, whether or not the results are beneficial to the investigational drug. The investigator must promise that no data relevant to the study and/or study results should be published on journals and academic or commercial conferences without written permission from the sponsor, and also understand that Hengrui will not disapprove the publication without reasons.

However, the investigator should inform the sponsor in advance to review any proposed publication or other forms of release before submission or publication to prevent unintentional leakage of confidential information or unprotected inventions. The investigator should provide Hengrui with the original draft, abstract or full text of the planned publication (poster, invited lectures, or guest lectures) at least 45 days prior to its submission for publication or other forms of disclosure. Hengrui will carry out a regulatory and intellectual property examination on the content of the publication. To protect the intellectual property, especially before the acquisition of patent, the investigator should agree to delay or cancel the publication. Prior to public release, Hengrui can require the investigator to delete any previously unpublished confidential information. If this study is part of a multicenter study, the investigator must agree that the first publication is an integrated result from all study sites. However, if a manuscript of the integrated analysis is not submitted within 12 months of completion or termination of the study at all study sites, the investigator can request to publish the study results from a single site, but Hengrui must be informed of and discussed with in advance.

The investigator is not allowed to mention the name(s) of sponsor and/or its employees in their promotional materials or publications before obtaining written agreement from the sponsor. In the meantime, sponsors are not allowed to use the investigator's name in promotional materials or publications before obtaining written agreement from the investigator and/or collaborator.

17. CLINICAL STUDY PROGRESS

Enrollment of first subject: Jan. 2018

Estimated time of enrollment completion: Jun. 2021

Estimated time of statistical summary: Dec. 2021

18. REFERENCES

[1] Liyan Wang, Zhiqiang Tang. Research progress of anti-tumor drug irinotecan. Foreign Medical Sciences (Section of Pharmacy). 2004, 31(1): 7-11.

[2] Xiangzhen Song, Bin Cao, Ziqiang Zhang, *et al*. Research progress on the *in vivo* metabolic pathway and metabolites of irinotecan. Northwest Pharmaceutical Journal. 2009, 24(6): 515-516.

[3] Package insert of Campto®.

[4] Chang T.C., *et al*. Phase I study of nanoliposomal irinotecan (PEP02) in advanced solid tumor patients. Cancer Chemother Pharmacol. 2015.

[5] ASCO Meeting. 2012 Poster. Phase I Study of Biweekly Liposome Irinotecan (PEP02, MM-398) in Metastatic Colorectal Cancer After First-line Oxaliplatin-based Chemotherapy.

[6] ASCO Meeting. 2015 Poster. PEPCOL: A randomized noncomparative phase II study of PEP02 (MM-398) or irinotecan in combination with leucovorin and 5-fluorouracil as second-line therapy in patients with unresectable metastatic colorectal cancer - A GERCOR Study.

[7] Ko A. H., *et al*. A multinational phase 2 study of nanoliposomal irinotecan sucrosofate (PEP02, MM-398) for patients with gemcitabine refractory metastatic pancreatic cancer. British Journal of Cancer (2013) 109, 920-925.

[8] Roy A. C., *et al*. A randomized phase II study of PEP02 (MM-398), irinotecan or docetaxel as a second-line therapy in patients with locally advanced or metastatic gastric or gastro-oesophageal junction adenocarcinoma. Annals of Oncology (2013)24: 1567-1573.

[9] Andrea W.G., *et al*. Nanoliposomal irinotecan with fluorouracil and folinic acid in metastatic pancreatic cancer after previous gemcitabine-based therapy (NAPOLI-1): a global, randomised, open-label, phase 3 trial. Lancet. 2015: S0140-6736(15)00986-1.

[10] Batist G, *et al*. Safety, Pharmacokinetics, and Efficacy of CPX-1 Liposome Injection in Patients with Advanced Solid Tumors. Clin Cancer Res. 2009, 15(2): 692-700.

[11] ASCO Meeting. 2008. A multi-center, phase 2 study of CPX-1 liposome injection in patients with advanced colorectal cancer.

[12] ASCO Meeting. 2009. Phase I and pharmacokinetic (PK) study of IHL-305 (pegylated liposomal irinotecan) in patients with advanced solid tumors.

[13] Package insert of Onivyde®, FDA.

19. APPENDICES

Appendix 1 TNM Staging for Pancreatic Cancer

The TNM staging for pancreatic cancer is based on American Joint Committee on Cancer (AJCC) Cancer Staging Manual (2017, 8th Edition).

AJCC TNM Staging for Pancreatic Cancer (8th Edition)

**Primary Tumor (T)**			
Tx	Primary tumor cannot be assessed		
T0	No evidence of primary tumor		
Tis	Carcinoma *in situ* (This includes high-grade pancreatic intraepithelial neoplasia (PanIN-3), intraductal papillary mucinous neoplasm with high-grade dysplasia, intraductal tubulopapillary neoplasm with high-grade dysplasia, and mucinous cystic neoplasm with high-grade dysplasia)		
T1	Tumor ≤ 2 cm in greatest dimension; 1a: tumor ≤ 0.5 cm in greatest dimension; 1b: tumor > 0.5 cm and < 1 cm in greatest dimension; 1c: tumor 1-2 cm in greatest dimension		
T2	Tumor > 2 cm and ≤ 4 cm in greatest dimension		
T3	Tumor > 4 cm in greatest dimension		
T4	Tumor involves the celiac axis, superior mesenteric artery, and/or common hepatic artery, regardless of size		
**Regional Lymph Nodes (N)**		**Distant Metastasis (M)**	
Nx	Regional lymph nodes cannot be assessed	M0	No distant metastasis
N0	No regional lymph node metastasis	M1	Distant metastasis
N1	Metastasis in 1-3 regional lymph nodes		
N2	Metastasis in ≥ 4 regional lymph nodes		
**Clinical Stage**			
Stage 0	Tis N0 M0		
Stage IA	T1N0 M0		
Stage IB	T2 N0 M0		
Stage IIA	T3 N0 M0		
Stage IIB	T1N1 M0， T2 N1 M0， T3 N1 M0，		
Stage III	Any T N2 M0, T4 Any N M0,		
Stage IV	Any T Any N M1		

Appendix 2 ECOG Performance Status Scoring Criteria

Score	Description
0	Asymptomatic, fully active, able to carry on all pre-disease performance without restriction.
1	Symptomatic, restricted in physically strenuous activity but ambulatory and able to carry out work of a light or sedentary nature, e.g., light house work, office work.
2	Symptomatic, ambulatory and capable of all self-care but unable to carry out any physical activities; up and about more than 50% of waking hours (confined to bed < 50% of waking hours).
3	Symptomatic, capable of only limited self-care; confined to bed or chair more than 50% of waking hours, but not totally confined to bed.
4	Completely disabled; cannot carry on any self-care; totally confined to bed or chair.
5	Death.

Appendix 3 NRS Pain Scoring Criteria

The numeric rating scale (NRS) uses 0-10 to represent different intensities of pain, with 0 representing no pain and 10 unbearable pain. Ask the patient "How intense is your pain?", or ask the patient to circle the number that fits best to his/her pain intensity. The rating criteria for pain are: 0: no pain; 1-3: mild pain; 4-6: moderate pain; 7-10: severe pain. The pain represented by each number is described in the table below:

**Pain Scale**	**Description**	
No Pain	No pain	0 points: no pain
Mild Pain	Pain when turning over, coughing, or taking a deep breath	1 point: no pain when lying quietly, pain when turning over and coughing
		2 points: pain when coughing, no pain when taking a deep breath
		3 points: no pain when lying quietly, pain when coughing and taking a deep breath
Moderate Pain	Pain when lying quietly, affecting sleep	4 points: intermittent pain when lying quietly<br>(≥ 4 points: quality of life is affected)
		5 points: persistent pain when lying quietly
		6 points: obvious pain when lying quietly
Severe Pain	Toss and turn, unable to fall asleep, sweating all over, unbearable	7 points: obvious pain, toss and turn, unable to fall asleep
		8 points: persistent and unbearable pain, sweating all over
		9 points: intense and unbearable pain
		10 points: extreme pain, to live is no better than to die

Appendix 4 NYHA Function Classification

Class	Description
I:	No limitation of physical activity. Ordinary physical activity does not cause symptoms of cardiac insufficiency.
II (i.e., Grade 1 heart failure):	Slight limitation of physical activity. Ordinary physical activity results in fatigue, palpitation, or dyspnea.
III (i.e., Grade 2 heart failure):	Marked limitation of physical activity. Less than ordinary activity causes fatigue, palpitation, or dyspnea.
IV (i.e., Grade 3 heart failure):	Extreme limitation of physical activity. Unable to carry on any physical activity without discomfort. Symptoms of heart failure at rest.

Appendix 5 Prohibited Traditional Chinese Medicine (TCM)

**Item**	**Drug Name**
Prohibited Traditional Chinese Medicine (TCM)	Huatan Huisheng tablets, *Brucea javanica* oil soft capsules, Mandarin melon berry syrup, cantharidin, cinobufotalin, bufotoxin, Kang'ai Injection, Kanglaite, *Sarcandra glabra* injection, Aidi injection, Awei Huapi cream, Kang'aiping pills, Fukang capsules, Xiaoaiping, Pingxiao capsules, Pingxiao tablets, Shendan Sanjie capsules, Ankangxin capsules, Bosheng'aining, Zedoary turmeric oil and glucose injection, Kanglixin capsules, and Cidan capsules.

Appendix 6 Percent Bone Marrow Content in Human Skeleton

Percent Bone Marrow in the Adult Skeleton

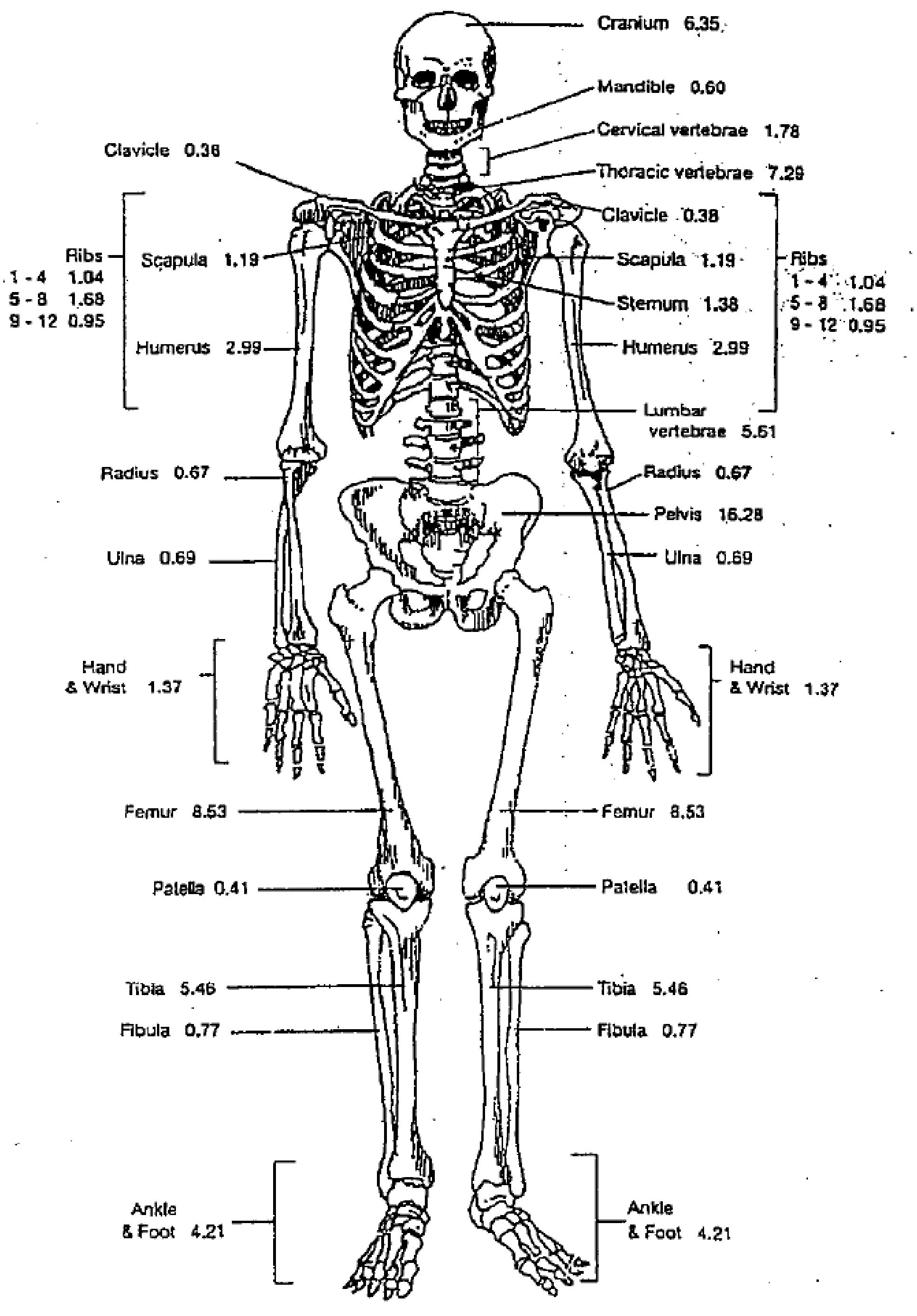

Woodward Holodny E. A summary of the data of Mechanik on the distribution of human bone marrow. Phys Med Biol. 1960;5:57-59

Appendix 7 Relevant Calculation Formulas

- **Calculation of body surface area (Stevenson's formula)**

Body surface area (m^2) = 0.0061 × height (cm) + 0.0128 × body weight (kg) - 0.1529

- **Calculation of creatinine clearance (Cockcroft-Gault formula)**

Please choose the appropriate formula corresponding to the unit of serum creatinine test:

If the unit of serum concentration of creatinine is mg/dL

Creatinine clearance in males (mL/min) = $\frac{(140-\text{age})\times(\text{body weight})}{72\times\text{serum creatinine}}$

Creatinine clearance in females (mL/min) = $\frac{0.85\times(140-\text{age})\times(\text{body weight})}{72\times\text{serum creatinine}}$

If the unit of serum concentration of creatinine is μmol/L

Creatinine clearance in males (mL/min) = $\frac{(140-\text{age})\times(\text{body weight})}{0.818\times\text{serum creatinine}}$

Creatinine clearance in females (mL/min) = $\frac{0.85\times(140-\text{age})\times(\text{body weight})}{0.818\times\text{serum creatinine}}$

Note: The unit of age is years old, and the unit of body weight is kilogram (kg).

Appendix 8 Quality of Life Score

EORTC QLQ-C30 (V3.0)

We are interested in some things about you and your health. Please answer all of the questions yourself by circling the number that best applies to you. There are no "right" or "wrong" answers. Simply choose the answer that best fits you. The information that you provide will be kept strictly confidential.

Patient Signature: ______________________

Date of Birth (DDMMYY): _____________

Today's Date (DDMMYY): _____________

	Not at all	A little bit	Quite a bit	Very much
1. Do you have any trouble doing strenuous activities, like carrying a heavy shopping bag or a suitcase?	1	2	3	4
2. Do you have any trouble taking a long walk?	1	2	3	4
3. Do you have any trouble taking a short walk outside of the house?	1	2	3	4
4. Do you need to stay in bed or a chair during the day?	1	2	3	4
5. Do you need help with eating, dressing, washing yourself or using the toilet?	1	2	3	4
**During the past week**	**Not at all**	**A little bit**	**Quite a bit**	**Very much**
6. Were you limited in doing either your work or other daily activities?	1	2	3	4
7. Were you limited in pursuing your hobbies or other leisure time activities?	1	2	3	4
8. Were you short of breath?	1	2	3	4
9. Have you had pain?	1	2	3	4
10. Did you need to rest?	1	2	3	4
11. Have you had trouble sleeping?	1	2	3	4
12. Have you felt weak?	1	2	3	4
13. Have you lacked appetite?	1	2	3	4
14. Have you felt nauseated?	1	2	3	4
15. Have you vomited?	1	2	3	4
16. Have you been constipated?	1	2	3	4
17. Have you had diarrhea?	1	2	3	4
18. Were you tired?	1	2	3	4

19. Did pain interfere with your daily activities?	1	2	3	4
20. Have you had difficulty in concentrating on things, like reading a newspaper or watching television?	1	2	3	4
21. Did you feel tense?	1	2	3	4
22. Did you worry?	1	2	3	4
23. Did you feel irritable?	1	2	3	4
24. Did you feel depressed?	1	2	3	4
25. Have you had difficulty remembering things?	1	2	3	4
26. Has your physical condition or medical treatment interfered with your family activities?	1	2	3	4
27. Has your physical condition or medical treatment interfered with your social activities?	1	2	3	4
28. Has your physical condition or medical treatment caused you financial difficulties?	1	2	3	4

For the following questions, please circle the number between 1 and 7 that best applies to you

29. How would you rate your overall health during the past week?

1	2	3	4	5	6	7
Very poor						Excellent

30. How would you rate your overall quality of life during the past week?

1	2	3	4	5	6	7
Very poor						Excellent

1. Quality of life score instructions:

EORTC QLQ-C30 (V3.0) is a core scale for all cancer patients, including a total of 30 items. Items 29 and 30 have a 7-point scale, scoring 1-7 points depending on the answer. Other items have a 4-point scale: Not at all, A little bit, Quite a bit, and Very much, scoring 1-4 points.

2. Calculation of EORTC QLQ-C30 scale (dimension) score (raw score):

For the convenience of statistical analysis and application, the scale is often divided into scales. A scale is an aspect of a quality of life component, also known as a dimension, which is analyzed as an independent variable. The EORTC QLQ-C30 (V3.0) scale comprises 30 items divided into 15 scales, including 5 functional scales (physical, role, cognitive, emotional, and social), 3 symptom scales (fatigability, pain, and nausea/vomiting), 1 global health status/quality of life scale, and 6 single-item scales (each one is a scale). Refer to the following for breakdown of these domains.

To get the score of each scale, add up the scores of the items in each scale and divide by the number of items in each scale (raw score, RS), that is, RS = (Q1 + Q2 + ... + Qn)/n.

Classification of various scales in EORTC QLQ-C30

	Items	Item Number
Physical Functioning	5	1-5
Role Functioning	2	6-7
Emotional Functioning	4	21-24
Cognitive Functioning	2	20-25
Social Functioning	2	26-27
Global Health Status	2	29-30
Fatigability	3	10, 12, 18
Nausea and Vomiting	2	14-15
Pain	2	9, 19
Dyspnea	1	8
Insomnia	1	11
Appetite Loss	1	13
Constipation	1	16
Diarrhea	1	17
Financial Difficulties	1	28

3. Calculation of EORTC QLQ-C30 standard score

To compare the scores between each scale, a linear transformation is further carried out to standardize the RS so that the standard score (SS) ranges from 0-100. Moreover, another purpose of the transformation is to reverse the direction of the score. Except for items 29 and 30 which are reversed items (the larger the score, the worse the quality of life), the scoring rules for QLQ-C30 clearly state that the higher the score for functional scale and global health status, the higher level of functioning and QoL, but a high score for symptom scale represents a high level of symptoms/problems (worse QoL). Therefore, the score of functional scale needs to be reversed when being standardized. Specifically, the following formula is used (where R is the full score range for each scale or item).

Functional scale: $SS = [1 - (RS - 1)/R] \times 100$

Symptom scale and global health status: $SS = [(RS - 1)/R] \times 100$

Appendix 9 Response Evaluation Criteria in Solid Tumors RECIST Version 1.1

This appendix is translated internally and is for reference only. Please refer to the original English version during practice.

1 MEASURABILITY OF TUMOR AT BASELINE

1.1 Definitions

At baseline, tumor lesions/lymph nodes will be categorized measurable or non-measurable as follows: **Measurable**

Tumor lesions: Must be accurately measured in at least one dimension (longest diameter in the plane of measurement is to be recorded) with a minimum size of:

- 10 mm by CT scan (CT scan slice thickness no greater than 5 mm)
- 10 mm caliper measurement by clinical exam (lesions which cannot be accurately measured with calipers should be recorded as non-measurable)
- 20 mm by chest X-ray
- Malignant lymph nodes: To be considered pathologically enlarged and measurable, a lymph node must be ≥ 15 mm in short axis when assessed by CT scan (CT scan slice thickness recommended to be no greater than 5 mm). At baseline and in follow-up, only the short axis will be measured and followed.

Non-measurable

All other lesions, including small lesions (longest diameter < 10 mm or pathological lymph nodes with ≥ 10 to < 15 mm short axis) as well as truly non-measurable lesions. Lesions considered truly non-measurable include: leptomeningeal disease, ascites, pleural or pericardial effusion, inflammatory breast disease, lymphangitic involvement of skin or lung, abdominal masses/abdominal organomegaly identified by physical exam that is not measurable by reproducible imaging techniques.

Special considerations regarding lesion measurability

Bone lesions, cystic lesions, and lesions previously treated with local therapy require particular comment:

Bone lesions:

- Bone scan, PET scan or plain films are not considered adequate to measure bone lesions. However, these techniques can be used to confirm the presence or disappearance of bone lesions;
- Lytic bone lesions or mixed lytic-blastic lesions, with identifiable soft tissue components, that can be evaluated by cross sectional imaging techniques such as CT or MRI can be considered as measurable lesions if the soft tissue component meets the definition of measurability described above;
- Blastic bone lesions are non-measurable.

Cystic lesions:

- Lesions that meet the criteria for radiographically defined simple cysts should not be considered as malignant lesions (neither measurable nor non-measurable) since they are, by definition, simple cysts;
- 'Cystic lesions' thought to represent cystic metastases can be considered as measurable lesions, if they meet the definition of measurability described above. However, if noncystic lesions are present in the same patient, these are preferred for selection as target lesions.
- Lesions with prior local treatment;
- Tumor lesions situated in a previously irradiated area, or in an area subjected to other loco-regional therapy, are usually not considered measurable unless there has demonstrated progression in the lesion. Study protocols should detail the conditions under which such lesions would be considered measurable.

1.2 Specifications by Methods of Measurements

Measurement of lesions

All measurements should be recorded in metric notation, using calipers if clinically assessed. All baseline evaluations should be performed as close as possible to the treatment start and never more than 28 days (4 weeks) before the beginning of the treatment.

Method of assessment

The same method of assessment and the same technique should be used to characterize each identified and reported lesion at baseline and during follow-up. Imaging based evaluation should always be done rather than clinical examination unless the lesion(s) being followed cannot be imaged but are assessable by clinical exam.

Clinical lesions: Clinical lesions will only be considered measurable when they are superficial and ≥ 10 mm diameter as assessed using calipers (e.g., skin nodules). For the case of skin lesions, documentation by color photography including a ruler to estimate the size of the lesion is suggested. As noted above, when lesions can be evaluated by both clinical exam and imaging, imaging evaluation should be undertaken since it is more objective and may also be reviewed at the end of the study.

Chest X-ray: Chest CT is preferred over chest X-ray, particularly when progression is an important endpoint, since CT is more sensitive than X-ray, particularly in identifying new lesions. However, lesions on chest X-ray may be considered measurable if they are clearly defined and surrounded by aerated lung.

CT, MRI: CT is the best currently available and reproducible method to measure lesions selected for response assessment. This guideline has defined measurability of lesions on CT scan based on the assumption that CT slice thickness is ≤ 5 mm. When CT scans have slice thickness greater than 5 mm, the minimum size for a measurable lesion should be twice the slice thickness. MRI is also acceptable in certain situations (e.g., for body scans).

Ultrasound: Ultrasound is not useful in assessment of lesion size and should not be used as a method of measurement. Ultrasound examinations cannot be reproduced in their entirety for independent review at a later date and, because they are operator dependent, it cannot be guaranteed that the same technique and measurements will be taken from one assessment to the next. If new lesions are identified by ultrasound in the course of the study, confirmation by CT or MRI is advised. If there is concern about radiation exposure at CT, MRI may be used instead.

Endoscopy, laparoscopy: The utilization of these techniques for objective tumor evaluation is not advised. However, they can be useful to confirm complete pathological response when biopsies are obtained or to determine relapse in trials where recurrence following complete response or surgical resection is an endpoint.

Tumor markers: Tumor markers alone cannot be used to assess objective tumor response. If markers are initially above the upper normal limit, however, they must normalize for a patient to be considered in complete response. Because tumor markers are disease specific, instructions for their measurement should be incorporated into protocols on a disease specific basis. Specific guidelines for both CA-125 response (in recurrent ovarian cancer) and PSA response (in recurrent prostate cancer) have been published. In addition, the Gynecologic Cancer Intergroup has developed CA-125 progression criteria which are to be integrated with objective tumor assessment for use in first-line trials in ovarian cancer.

Cytology, histology: These techniques can be used to differentiate PR and CR in specific cases required by protocol (e.g., residual lesions in tumor types such as germ cell tumors, where known residual benign tumors can remain). When effusions are known to be a potential adverse effect of treatment (e.g., with certain taxane compounds or angiogenesis inhibitors), the cytological confirmation of the neoplastic origin of any effusion that appears or worsens during treatment can be considered if the measurable tumor has met the criteria for response or stable disease in order to differentiate between response (or SD) and PD.

2 TUMOR RESPONSE EVALUATION

2.1 Evaluation of Target Lesion

Complete response (CR): Disappearance of all target lesions. Any pathological lymph nodules (whether target or non-target) must have reduction in short axis to < 10 mm.

Partial response (PR): At least a 30% decrease in the sum of diameters of target lesions, compared with baseline.

Progressive disease (PD): At least a 20% increase in the sum of diameters of target lesions, taking as reference the smallest sum on study (this includes the baseline sum if that is the smallest on study). In addition to the relative increase of 20%, the sum must also demonstrate an absolute increase of at least 5 mm. (Note: the appearance of one or more new lesions is also considered progression).

Stable disease (SD): Neither sufficient shrinkage to qualify for PR nor sufficient increase to qualify for PD, taking as reference the smallest sum diameters while on study.

2.2 Special Notes on the Assessment of Target Lesions

Lymph nodes: Lymph nodes identified as target lesions should always have the actual short axis measurement recorded (measured in the same anatomical plane as the baseline examination), even if the nodes regress to below 10 mm on study. This means that when lymph nodes are included as target lesions, the 'sum' of lesions may not be zero even if complete response criteria are met, since a normal lymph node is defined as having a short axis of < 10 mm. Case report forms or other data collection methods may therefore be designed to have target nodal lesions recorded in a separate section where, in order to qualify for CR, each node must achieve a short axis < 10 mm. For PR, SD and PD, the actual short axis measurement of the nodes is to be included in the sum of target lesions.

Target lesions that become too small to measure: While on study, all lesions (nodal and non-nodal) recorded at baseline should have their actual measurements recorded at each subsequent evaluation, even when very small (e.g., 2 mm). However, sometimes lesions or lymph nodes which are recorded as target lesions at baseline become so faint on CT scan that the radiologist may not feel comfortable assigning an exact measure and may report them as being "too small to measure". When this occurs, it is important that a value be recorded on the case report form. If it is the opinion of the radiologist that the lesion has likely disappeared, the measurement should be recorded as 0 mm. If the lesion is believed to be present and is faintly seen but too small to measure, a default value of 5 mm should be assigned. (Note: It is less likely that this rule will be used for lymph nodes since they usually have a definable size when normal and are frequently surrounded by fat such as in the retroperitoneum; however, if a lymph node is believed to be present and is faintly seen but too small to measure, a default value of 5 mm should be assigned in this circumstance as well). This default value is derived from the 5 mm CT slice thickness (but should not be changed with varying CT slice thickness). The measurement of these lesions is potentially non-reproducible, therefore providing this default value will prevent false responses or progressions based upon measurement error. To reiterate, however, if the radiologist is able to provide an actual measure, that should be recorded, even if it is below 5 mm.

Lesions that split or coalesce: When non-nodal lesions fragmented, the longest diameters of the fragmented portions should be added together to calculate the target lesion sum. Similarly, as lesions coalesce, a plane between them may be maintained that would aid in obtaining maximal diameter measurements of each individual lesion. If the lesions have truly coalesced such that they are no longer separable, the vector of the longest diameter in this instance should be the maximal longest diameter for the coalesced lesion.

2.3 Evaluation of Non-Target Lesions

This section provides the definitions of the criteria used to determine the tumor response for the group of non-target lesions. While some non-target lesions may actually be measurable, they need not be measured and instead should be assessed only qualitatively at the time points specified in the protocol.

Complete response (CR): Disappearance of all non-target lesions and normalization of tumor marker level. All lymph nodules must be non-pathological in size (< 10 mm short axis).

Non-CR/Non-PD: Persistence of one or more non-target lesion(s) and/or maintenance of tumor marker level above the normal limits.

Progressive disease (PD): Unequivocal progression of existing non-target lesions. Note: the appearance of one or more new lesions is also considered as progression.

2.4 Special Notes on Assessment of Progression of Non-Target Disease

The concept of progression of non-target disease requires additional explanation as follows: When the patient also has measurable disease: to achieve unequivocal progression on the basis of the non-target disease, there must be an overall level of substantial worsening in non-target disease such that, even in presence of SD or PR in target disease, the overall tumor burden has increased sufficiently to merit discontinuation of therapy. A modest increase in the size of one or more non-target lesions is usually not sufficient to quality for unequivocal progression status. The designation of overall progression solely on the basis of change in non-target disease in the face of SD or PR of target disease will therefore be extremely rare.

The case of the patient having only non-measurable lesions arises in some phase III trials when there is not a criterion of study entry to have measurable lesions. The same general concepts apply here as noted above, however, in this instance there is no measurable disease assessment to factor into the interpretation of an increase in non-measurable disease burden. Because worsening in non-target disease cannot be easily quantified (by definition: if all lesions are truly non-measurable), a useful test that can be applied when assessing patients for unequivocal progression is to consider if the increase in overall disease load based on the change in non-measurable disease is comparable in magnitude to the increase that would be required to declare PD for measurable disease. E.g. an increase in tumor burden representing an additional 73% increase in 'volume' (which is equivalent to a 20% increase diameter in a measurable lesion). Examples include an increase in a pleural effusion from "trace" to "large", an increase in lymphangitic disease from localized to widespread, or may be described in protocols as "sufficient to require a change in treatment". Examples include an increase in a pleural effusion from trace to

large, an increase in lymphangitic disease from localized to widespread, or may be described in protocols as "sufficient to require a change in therapy". If unequivocal progression is seen, the patient should be considered to have had overall PD at that point. While it would be ideal to have objective criteria to apply to non-measurable disease, the very nature of that disease makes it impossible to do so, therefore the increase must be substantial.

2.5 New Lesions

The appearance of new malignant lesions denotes disease progression; therefore, some comments on detection of new lesions are important. There are no specific criteria for the identification of new radiographic lesions; however, the finding of a new lesion should be unequivocal: i.e. not attributable to differences in scanning technique, change in imaging modality or findings thought to represent something other than tumor (for example, some 'new' bone lesions may be simply healing or flare of pre-existing lesions). This is particularly important when the patient's baseline lesions show partial or complete response. For example, necrosis of a liver lesion may be reported on a CT scan report as a new cystic lesion, which it is not.

A lesion identified on a follow-up study in an anatomical location that was not scanned at baseline is considered a new lesion and will indicate disease progression. An example of this is the patient who has visceral disease at baseline and while on study has a CT or MRI brain ordered which reveals metastases. The patient's brain metastases are considered to be evidence of PD even if he/she did not have brain imaging at baseline.

If a new lesion is equivocal, for example, because of its small size, continued therapy and follow-up evaluation will clarify if it represents truly new disease. If repeat scans confirm there is definitely a new lesion, then progression should be declared using the date of the initial scan.

While FDG-PET response assessments generally need additional study, it is sometimes reasonable to incorporate the use of FDG-PET scanning to complement CT scanning in assessment of progression (particularly possible new disease). New lesions on the basis of FDG-PET imaging can be identified according to the following algorithm:

Negative FDG-PET at baseline, with a positive FDG-PET at follow-up is a sign of PD based on a new lesion.

No FDG-PET at baseline and a positive FDG-PET at follow-up:

If the positive FDG-PET at follow-up corresponds to a new site of disease confirmed by CT, this is PD.

If the positive FDG-PET at follow-up is not confirmed as a new site of disease on CT, additional follow-up CT scans are needed to determine if there is truly progression occurring at that site (if so, the date of PD will be the date of the initial abnormal FDG-PET scan).

If the positive FDG-PET at follow-up corresponds to a pre-existing site of disease on CT that is not progressing on the basis of the anatomic images, this is not PD.

2.6 Missing Assessments and Inevaluable Designation

When no imaging/measurement is done at all at a particular time point, the patient is not evaluable (NE) at that time point. If only a subset of lesion measurements is made at an assessment, usually the case is also considered NE at that time point, unless a convincing argument can be made that the contribution of the individual missing lesion(s) would not change the assigned time point response.

2.7 Special Notes on Response Assessment

When nodal disease is included in the sum of target lesions and the nodes decrease to 'normal' size (< 10 mm), they may still have a measurement reported on scans. This measurement should be recorded even though the nodes are normal in order not to overstate progression should it be based on increase in size of the nodes. As noted earlier, this means that patients with CR may not have a total sum of zero on the case report form (CRF).

In trials where confirmation of response is required, repeated "NE" time point assessments may complicate best response determination. The analysis plan for the trial must specify how missing data/assessments will be addressed in determination of response and progression. For example, in most trials it is reasonable to consider a patient with time point responses of PR-NE-PR as a confirmed response.

Patients with a global deterioration of health status requiring discontinuation of treatment without objective evidence of disease progression at that time should be reported as symptomatic deterioration. Every effort should be made to evaluate objective progression even after discontinuation of treatment. Symptomatic deterioration is not a descriptor of an objective response: it is a reason for stopping study therapy. The objective response status of such patients is to be determined by evaluation of target and non-target disease as shown in Attached Tables 1-3.

Conditions that be defined as 'early progression, early death and NE' are study specific and should be clearly described in each protocol (depending on treatment duration and treatment periodicity).

In some circumstances, it may be difficult to distinguish residual disease from normal tissues. When the evaluation of complete response depends upon such a definition, it is recommended that the residual lesion be investigated (fine needle aspirate/biopsy) before assigning a status of complete response. FDG-PET may be used to upgrade a response to a CR in a manner similar to a biopsy in cases where a residual radiographic abnormality is thought to represent fibrosis or scarring. The use of FDG-PET in this circumstance should be prospectively described in the protocol and supported by disease specific medical literature for the indication. However, it must be acknowledged that both approaches may lead to false positive CR due to limitations of FDG-PET and biopsy resolution/sensitivity.

Attached Table 1. Time point response: patients with target (+/– non-target) disease

Target Lesions	Non-Target Lesions	New Lesions	Overall Response
CR	CR	No	CR
CR	Non-CR/non-PD	No	PR
CR	Not evaluated	No	PR
PR	Non-PD or not all evaluated	No	PR
SD	Non-PD or not all evaluated	No	SD
Not all evaluated	Non-PD	No	NE
PD	Any	Yes or No	PD
Any	PD	Yes or No	PD
Any	Any	Yes	PD

Note: CR = complete response, PR = partial response, SD = stable disease, PD = progressive disease, NE = non-evaluable.

Attached Table 2. Time point response: patients with non-target disease only

Non-Target Lesions	New Lesions	Overall Response
CR	No	CR
Non-CR/Non-PD	No	Non-CR/Non-PD
Not all evaluated	No	Not evaluated
Equivocal PD	Yes or No	PD
Any	Yes	PD

Note: "Non-CR/non-PD" is preferred over SD for non-target disease. Since SD is increasingly used as endpoint for assessment of efficacy in some trials, so to assign this category when no lesions can be measured is not advised.

For equivocal findings of progression (e.g., very small and uncertain new lesions; cystic changes or necrosis in existing lesions), treatment may continue until the next scheduled assessment. If at the next scheduled assessment, progression is confirmed, the date of progression should be the earlier date when progression was suspected.

Attached Table 3. Best overall response when confirmation of CR and PR required

Overall Response at First Time Point	Overall Response at Subsequent Time Point	Best Overall Response
CR	CR	CR
CR	PR	SD, PD, or PR[a]
CR	SD	SD (provided minimum criteria for SD duration met, otherwise, PD)
CR	PD	SD (provided minimum criteria for SD duration met, otherwise, PD)
CR	NE	SD (provided minimum criteria for SD duration met, otherwise, NE)
PR	CR	PR
PR	PR	PR
PR	SD	SD
PR	PD	SD (provided minimum criteria for SD duration met, otherwise, PD)
PR	NE	SD (provided minimum criteria for SD duration met, otherwise, NE)
NE	NE	NE

Note: CR = complete response, PR = partial response, SD = stable disease, PD = progressive disease, NE = non-evaluable. [a]: If a CR is truly met at first time point, then any disease seen at a subsequent time point, even the disease meeting PR criteria relative to baseline, makes the disease PD at that point (since disease must have reappeared after CR). Best response would depend on whether minimum duration for SD was met. However, sometimes CR may be claimed when subsequent scans suggest small lesions were likely still present and in fact the patient had PR, not CR at the first time point. Under these circumstances, the original CR should be changed to PR and the best response is PR.

2.8 Confirmatory Measurement/Duration of Response

Efficacy confirmation

In non-randomized trials where tumor response is the primary endpoint, confirmation of PR and CR is required to ensure responses identified are not the result of measurement error. However, in studies where stable disease or progression is the primary endpoint, confirmation of response is not required since it will not add value to the interpretation of trial results. In the case of SD, measurements must have met the SD criteria at least once after study entry at a minimum interval (in general not less than 6-8 weeks) that is defined in the study protocol.

Duration of overall response

The duration of overall response is measured from the time measurement criteria are first met for CR/PR (whichever is first recorded) until the first date that recurrent or progressive disease is objectively documented (taking as reference for progressive disease the smallest measurements recorded on study). The duration of overall complete response is measured from the time measurement criteria are first met for CR until the first date that recurrent disease is objectively documented.

Duration of stable disease

Stable disease is measured from the start of the treatment (in randomized trials, from date of randomization) until the criteria for progression are met, taking as reference the smallest sum on study (if the baseline sum is the smallest, this is the reference for calculation of PD). The clinical relevance of the duration of stable disease varies in different studies and diseases. If the proportion of patients achieving stable disease for a minimum period of time is an endpoint in a particular trial, the protocol should specify the minimal time interval required between two measurements for determination of stable disease.

Note: The duration of response and stable disease as well as the progression-free survival are influenced by the frequency of follow-up after baseline evaluation. It is not in the scope of this guideline to define a standard follow-up frequency. The frequency should take into account many parameters including disease types and stages, treatment periodicity and standard practice. However, these limitations of the precision of the measured endpoint should be taken into account if comparisons between trials are to be made.

2.9 PFS/TTP

Many trials of advanced tumors use PFS or TTP as the primary endpoint. If the protocol requires the presence of measurable lesions in all patients, the evaluation of progress will be relatively simple. An increasing number of trials allow the participation of patients with and without measurable lesions. In this case, the clinical findings of progressive disease in patients without measurable lesions must be described in detail. Because there is often a deviation in the determination of the date of progress, the observation time points for different treatment groups should be the same.

A Phase III Clinical Study of Irinotecan Hydrochloride Liposome in Combination with 5-FU/LV in the Treatment of Pancreatic Cancer

Revisions of Study Protocol V1.1

Revision Location	Version 1.0	Version 1.1	Rationales for Changes
**Cover Page, Header, Footer, and Protocol Signature Page**	Version No.: 1.0; Version Date: 8 Jul., 2017	Version No.: 1.1; Version Date: 25 Oct., 2019	Version change
**Summary of Changes in the Protocol**	Added contents	Version No.: 1.1; Version Date: 25 Oct., 2019	Version change
**Inclusion Criteria**	1. Aged 18-75 years old	1. Male or female aged ≥ 18 years old	Corrected the description
	2. Pathologically confirmed pancreas adenocarcinoma (derived from pancreatic ductal epithelium), also unresectable locally advanced or metastatic pancreatic cancer as shown in clinical records;	2. Pathologically confirmed pancreatic cancer (derived from pancreatic ductal epithelium), also unresectable locally advanced or metastatic pancreatic cancer as shown in clinical records	Corrected the description
	3. Disease progression after prior gemcitabine-based therapy for locally advanced or metastatic disease (received at least 1 cycle of gemcitabine-based therapy, and developed disease progression within 6 months after the end of treatment with definite radiographic evidence of progression) as shown in clinical records, including but not limited to the following gemcitabine-based dosing regimens:<br>● Gemcitabine alone;	3. Treatment failure with gemcitabine-based systemic therapy as **first-line** treatment for locally advanced or metastatic disease (received at least 1 cycle of gemcitabine-based therapy, and developed PD or intolerance during treatment and PD after the end of treatment) as shown in clinical records, including but not limited to the following gemcitabine-based dosing regimens:<br>● Gemcitabine alone;<br>● Any gemcitabine-based regimen with or without maintenance treatment with gemcitabine;	Corrected the description<br>Added the description about intolerance for enrollment and the limit for neoadjuvant therapy

Revision Location	Version 1.0	Version 1.1	Rationales for Changes
Inclusion Criteria	● Any gemcitabine-based regimen with or without maintenance treatment with gemcitabine;<br>● Gemcitabine alone, followed by combination with platinum, fluorouracil, erlotinib, etc.;<br>● Adjuvant chemotherapy containing gemcitabine, and relapse within 6 months after the completion of treatment.	● Gemcitabine alone, followed by combination with platinum, fluorouracil, erlotinib, etc.;<br>● **Neoadjuvant/**adjuvant chemotherapy containing gemcitabine, and relapse within 6 months after the completion of treatment.<br>Note: Definition of intolerance: 1) Hematologic toxicities: Grade 3 neutropenia with fever of > 38.5 °C, Grade 3 platelets decreased with hemorrhage symptoms, and other Grade 4 or greater hematologic toxicities; 2) Non-hematologic toxicities: Grade 3 or greater non-hematologic toxicities; 3) The above-mentioned toxicities render the subject unsuitable to continue the original therapy as judged by the investigator.	
	6. BMI ≥ 18.5 kg/m^2	Deleted	Subjects with BMI < 18.5 kg/m^2 but with good ECOG PS can be enrolled in this study. Thus, extended the inclusion criteria for these subjects.
	8. Have not used hematopoietic growth factors within the last 7 days Albumin level ≥ 35 g/L	8. Have not used hematopoietic growth factors or undergone blood transfusion within the last 7 days Albumin level ≥ 30g/L	1. Excluded subjects who have undergone blood transfusion within the last 7 days<br>2. Extended the albumin level
	9. Previous surgery, radiotherapy, chemotherapy, or other anti-tumor therapy ended 4 weeks ago or earlier, and general physical conditions or related adverse reactions have recovered (toxicity ≤ Grade 1)	9. Previous surgery, radiotherapy, chemotherapy, or other anti-tumor therapy ended 4 weeks ago or earlier, and general physical conditions or related adverse reactions have recovered (toxicity ≤ Grade 1) or reached a stable state	1. Allowed subjects who reach a stable state to be enrolled.

Revision Location	Version 1.0	Version 1.1	Rationales for Changes
**Exclusion Criteria**	6. Serious cardiovascular and cerebrovascular arterial thromboembolism in the past 6 months (such as myocardial infarction, unstable angina, and stroke);<br>7. NYHA Class III or IV congestive cardiac failure, ventricular arrhythmia or hypertension that is difficult to control;	7. Poorly controlled cardiovascular and cerebrovascular diseases or clinical symptoms, including but not limited to: (1) NYHA Class ≥ III cardiac failure; (2) unstable angina; (3) myocardial infarction or stroke in the past 6 months; (4) supraventricular or ventricular arrhythmia requiring treatment or intervention; (5) hypertension that is difficult to control (systolic blood pressure > 150 mmHg and/or diastolic blood pressure > 90 mmHg after optimal treatment).	Corrected the description
**Drug Preparation Page 18**	After the infusion is completed, flush the IV line with 100 mL of 0.9% sodium chloride solution or 5% dextrose solution;	After the infusion is completed, flush the IV line with an appropriate amount of 0.9% sodium chloride solution or 5% dextrose solution;	Corrected the description
**Dosing Regimen Page 18**	Irinotecan liposome: 60 mg/m^2, intravenous infusion for 90 min, once every 2 weeks.<br>LV: 200 mg/m^2, intravenous infusion for 30 min, once every 2 weeks.<br>5-FU: 2000 mg/m^2, intravenous infusion for 46 h, once every 2 weeks.	Irinotecan liposome: 60 mg/m^2, intravenous infusion for at least 90 min, once every 2 weeks.<br>LV: 200 mg/m^2, intravenous infusion for 30 ± 10 min, once every 2 weeks.<br>5-FU: 2000 mg/m^2, intravenous infusion for 46 ± 4 h, once every 2 weeks.	Added the time window of infusion duration to facilitate clinical operation
**Dose modification due to non-hematologic toxicity Page 20/24**	All Grade 3-4 non-hematologic toxicities must recover to Grade 1 or baseline level before the next cycle of treatment	All Grade 3-4 non-hematologic toxicities must recover to Grade 1, baseline level, or stable state before the next cycle of treatment	Allowed the subject to start the next cycle of treatment when a stable state is reached due to that some toxicities are difficult to return to Grade 1 or baseline level.
**Study Procedures/Screening Period Page 33**	Added contents	(2) Study medication: Subjects must begin the assigned study treatment within 48 h after the randomization is completed.	Add contents to improve rigor.

Revision Location	Version 1.0	Version 1.1	Rationales for Changes
**Updated contents**			
**Background Phase Ib Clinical Study Page 11**	Phase Ib clinical study data	Phase Ib study results are updated including the enrollment progress, safety, and efficacy, which are detailed in the study protocol	**Updated contents**
**Diagnostic Criteria Page 14**	Diagnosed according to the "Expert Consensus of Comprehensive Diagnosis and Treatment of Pancreatic Cancer in China (Version 2014)"; staged as per American Joint Committee on Cancer (AJCC) Cancer Staging Manual (AJCC, 7th Edition)	Diagnosed according to the latest version of "CSCO Guidelines for the Diagnosis and Treatment of Pancreatic Cancer"; staged as per American Joint Committee on Cancer (AJCC) Cancer Staging Manual (AJCC 2017, 8th Edition)	**Updated contents**
**Contact for SAE reporting Page 43**	Huilong Liu, Clinical Medicine Department	Clinical Medicine Department<br>Email: hengrui_drug_safety@hrglobe.cn	**Updated contents**
**Clinical Study Progress Page 56**	Scheduled enrollment of first subject: Sep. 2017<br>Estimated time of enrollment completion: Sep. 2019<br>Estimated time of statistical summary: Dec. 2019	Enrollment of first subject: Jan. 2018<br>Estimated time of enrollment completion: Jun. 2021<br>Estimated time of statistical summary: Dec. 2021	**Updated contents**

Clinical Medicine Department II

Jiangsu Hengrui Pharmaceuticals Co., Ltd.

4 Nov., 2019

A Phase III Clinical Study of Irinotecan Hydrochloride Liposome in Combination with 5-FU/LV in the Treatment of Pancreatic Cancer

Revisions of Study Protocol V2.0

Revision Location	Version 1.1	Version 2.0	Rationales for Changes
**Cover Page, Header, Footer, and Protocol Signature Page**	Version No.: 1.1; Version Date: 25 Oct., 2019	Version No.: 2.0; Version Date: 28 Jan., 2021	Version change
**Summary of Changes in the Protocol**	Added contents	Version No.: 2.0; Version Date: 28 Jan., 2021	Version change
**List of Abbreviations**	Added contents	SC, Steering Committee	Added corresponding description of the interim analysis
**Protocol Synopsis: Study Design; 3.1 Overview of Study Design/Page 13**	The primary endpoint will be analyzed after approximately 252 deaths occur.	The primary endpoint will be analyzed after approximately 253 deaths occur.	Updated the number of death events according to the calculation results of sample size after adding interim analysis
	Added contents	In this study, blinded and unblinded steering committees (SCs) are planned to be set up, and an interim analysis will be conducted. After data review, the unblinded SC will provide recommendation on whether to submit application for registration in advance. See SC Charter for detailed information on composition, responsibilities, and procedures of SC.	Added corresponding description of the interim analysis

Revision Location	Version 1.1	Version 2.0	Rationales for Changes
**Protocol Synopsis: Sample Size Estimation; 3.2 Sample Size Estimation/Page 13**	With reference to the original NAPOLI-1 study data for pancreatic cancer (the OS was 6.1 months in the experimental group and 4.2 months in the control group) and the current status of pancreatic cancer in China, the median OS of the experimental group and the control group in this study is set as 5.0 months and 3.5 months, respectively, with significance level α = 0.025 (one-sided), a power of 80%, the enrollment duration of 24 months, the total study duration of 36 months, and a dropout rate of 20%. A design of experimental group:placebo group = 1:1 will be adopted. According to the sample size calculation formula of the log-rank test for the OS comparison between two groups (calculated by the PASS 11 software), a total of 272 subjects should be enrolled in this study (136 subjects in the experimental group and 136 subjects in the control group), and 252 deaths should be collected.	With reference to the original NAPOLI-1 study data for pancreatic cancer (the OS was 6.1 months in the experimental group and 4.2 months in the control group) and the current status of pancreatic cancer in China, the median OS of the experimental group and the control group in this study is set as 5.0 months and 3.5 months, respectively, with significance level α = 0.025 (one-sided), a power of 80%, the enrollment duration of 24 months, the total study duration of 36 months, and a dropout rate of 20%. A design of experimental group:placebo group = 1:1 will be adopted. An interim analysis is planned when 70% of OS events are collected to determine whether to conduct application for registration in advance. Based on the above parameters, at least 253 deaths should be collected and a total of 272 subjects should be enrolled (136 subjects in the experimental group and 136 subjects in the control group) according to the log-rank test for the OS comparison between the two groups and the O'Brien & Fleming type α spending function (EAST 6.5).	Added the description of sample size corresponding to the interim analysis
**Protocol Synopsis: Interim Analysis**	Added contents	In this study, one interim analysis will be performed for the primary efficacy endpoint. The main objectives of the interim analysis include but are not limited to:<br>1. Application for registration in advance due to superiority;<br>2. Continuation of the study as planned.<br>The interim analysis will be conducted when 70% of endpoint events (177) are collected. The superiority boundaries determined using the O'Brien & Fleming type α spending function are as follows:	Added corresponding description of the interim analysis

<table>
<tr><th>Revision Location</th><th>Version 1.1</th><th>Version 2.0</th><th>Rationales for Changes</th></tr>
<tr><td></td><td></td><td>
<table>
<tr><th>Time Point</th><th>Number of Endpoint (OS) Events</th><th>Superiority Boundary Z-Value (Corresponding HR)</th><th>One-Sided Nominal Significance Level of Superiority</th></tr>
<tr><td>Interim Analysis</td><td>177 (70%)</td><td>-2.438 (HR = 0.692)</td><td>0.007</td></tr>
<tr><td>Final Analysis</td><td>253</td><td>-2.000 (HR = 0.777)</td><td>0.023</td></tr>
</table>

Note: OS = Overall Survival;

The interim analyses will be completed by unblinded statisticians and their programming team. The interim analysis results will be reviewed by the unblinded SC, and the SC will determine whether to put forward the proposal of application for registration in advance to the sponsor according to the results. If the sponsor applies for registration in advance, the early unblinding will be conducted at the same time (see 12.4 for details).</td><td></td></tr>
<tr><td>Schedule of Activities</td><td>(11) Concomitant medication: All concomitant medications during the study should be recorded;</td><td>(11) Concomitant medication: Various treatments and drugs used from 30 days before signing the informed consent form to 30 days after the last dose should be recorded;</td><td>Further clearly described</td></tr>
<tr><td>1.5.3 Phase Ib clinical study/Page 11</td><td>Among 3 evaluable subjects at the dose of 80 mg/m^2, 1 subject achieved PR (nasopharyngeal carcinoma, completed 32 cycles of treatment) and 2 subjects achieved SD. Among 12 evaluable subjects at the dose of 60 mg/m^2, 1 subject achieved PR (nasopharyngeal carcinoma, completed 6 cycles of treatment), 7 subjects achieved SD, and 2 subjects achieved PD, indicating preliminary efficacy. Two subjects with pancreatic cancer were evaluated as SD. Among all 15 subjects, the ORR was 13.3%</td><td>The efficacy results showed that in terms of the best overall efficacy, the ORR was 26.7% (4/15), confirmed ORR was 13.3% (2/15), and DCR was 86.7% in the 15 subjects. Subjects with efficacy evaluated as PR: 3 subjects in the 60 mg/m^2 group (1 subject with ampullary tumor has undergone efficacy confirmation, 1 subject with nasopharyngeal carcinoma, 1 subject of breast cancer) and 1 subject in the 80 mg/m^2 group (1 subject with nasopharyngeal carcinoma has undergone efficacy confirmation), indicating promising anti-tumor activity.</td><td>Updated the study data</td></tr>
</table>

Revision Location	Version 1.1	Version 2.0	Rationales for Changes
	and the DCR was 86.7%, indicating preliminary anti-tumor activity.		
**3.5 Blinding and Procedures of Blinding Method/Page 14**	Added contents	Unblinded SC will be unblinded at the interim analysis. The interim analysis will be conducted by an unblinded statistician to ensure that the sponsor, investigators, and subjects remain blinded until the primary analysis of the study (253 death events).	Added the description of blind in the interim analysis
**5.2 Dosing Regimen/ Page 19**	Added contents	For the convenience of administration, the protocol allows a deviation of ± 5% between the actual total dose and the theoretical total dose each time.	To comply with the clinical practice standard
**6.2.3.1 Infusion reaction/Page 22**	The grade of infusion related reactions is established according to infusion reactions and allergic reactions in CTCAE 4.03, but with slight difference. See Table 6.4 for details.	The grade of infusion related reactions is established according to infusion reactions and allergic reactions in CTCAE 4.03, but with slight difference. Relevant measures and suggestions are provided for reference. See Table 6.4 for details.	Further detailed the description
**7.1 Information on Study Drugs/Pages 26-27**	Irinotecan liposome [Shelf life] 18 months (tentative)<br>Calcium folinate [Storage conditions] 2-8 °C, sealed, protected from light	Irinotecan liposome [Shelf life] 24 months<br>Calcium folinate [Storage conditions] 2-10 °C, sealed, protected from light	Updated contents
**8.2 Allowed Concomitant Medications and Treatments/ Page 31**	All treatments and medications (including the generic name, dose, route of administration, start time, end time, and indication) used concomitantly within 1 month prior to signing informed consent form and during the study should be documented in the eCRF in strict accordance with the GCP regulations. The vehicle of the drugs may not be recorded.	All treatments and medications (including the generic name, dose, route of administration, start time, end time, and indication) used concomitantly from 30 days prior to signing informed consent form to 30 days after the last dose should be documented in the eCRF in strict accordance with the GCP regulations. The vehicle of the drugs may not be recorded.	Further clearly described

Revision Location	Version 1.1	Version 2.0	Rationales for Changes
**10.2.1.1 Adverse events/Page 41**	An adverse event refers to any untoward medical occurrence in a study subject administered a pharmaceutical product. Any untoward medical occurrence after the subject receives the investigational drug until 30 days after the end of treatment is judged as an AE, regardless of the causality with the investigational drug.	An adverse event (AE) refers to any untoward medical occurrence in a study subject administered a pharmaceutical product. Any untoward medical occurrence after the subject receives the investigational drug until 30 days after the last dose is judged as an AE, regardless of the causality with the investigational drug.	Further detailed the description
**10.2.1.3 Judgment of causality between adverse events and study drugs/Page 42**	Based on the causality assessment criteria, the relationship between AEs and study drugs is classified into 5 categories: related, possibly related, unlikely related, not related, and unassessable. Events that are assessed to be related, possibly related, and unassessable will be listed as adverse drug reactions. The incidence of adverse drug reaction will be calculated using the total number of subjects with adverse drug reaction as the numerator and the total number of subjects included in the AE evaluation as the denominator. For details, refer to Table 10.2 below.	The judgment of causality between AEs and investigational drug is to determine whether the investigational drug has a reasonable possibility of causing or contributing to the AE through comprehensive evaluation. The judgment factors include whether there is a reasonable time sequence between the onset of AEs and the administration of investigational drug, the characteristics of the investigational drug, the toxicological and pharmacological effects of the investigational drug, the use of concomitant medications, the subject's underlying diseases, medical history, family history, dechallenge, and rechallenge. Generally, facts (evidence) or basis used to judge the causality should be provided.<br><br>The investigator should judge the causality between the AE and the investigational drug as "related", "possibly related", "unlikely related", "not related" and "unassessable".	Updated contents
**10.2.1.4 1) Definition of serious adverse event/Page 43**	The following unexpected medical events are included:	The following untoward medical events are included:	Corrigendum

Revision Location	Version 1.1	Version 2.0	Rationales for Changes
**10.2.1.4 2) Pregnancy/ Page 43**	Pregnancy occurring during the clinical study should be filled in the "Pregnancy Report Form" and reported as per the requirements for reporting serious adverse event.	For pregnancy occurring during the clinical study, the investigator should report the pregnancy to the sponsor within 24 h of knowing the event by filling out the "Hengrui Clinical Study Pregnancy Report/Follow-up Form".<br><br>The investigators should track a pregnancy event until its final outcomes (including any premature termination of pregnancy or childbirth), and childbirth should be followed up for 1 month after childbirth. The pregnancy outcomes should be reported to the sponsor. If pregnancy outcomes meet the SAE criteria (such as ectopic pregnancy, spontaneous abortion, intrauterine death, neonatal death, or congenital anomalies, etc.), they must be reported according to SAE procedures.<br><br>If a subject experiences any SAE during pregnancy, the SAE should be reported according to the SAE reporting procedure.<br><br>The email address for the sponsor to receive the report of pregnancy is: hengrui_drug_safety@hrglobe.cn	Specified the reporting time limit of pregnancy events and the reporting recipient as the sponsor.
**10.2.1.4 5) Hospitalization/ Page 44**	• Hospitalization due to the pre-existing disease without new AEs and aggravation of the pre-existing disease (e.g., hospitalization to examine laboratory abnormalities that have persisted before the study until now);<br>• Scheduled treatment or surgery that should be documented throughout the entire study protocol and/or in the subjects' individual baseline information;<br>• Hospitalization merely for use of blood products.	• Hospitalization due to pre-existing disease and unrelated to an AE, without aggravation of the pre-existing diseases (e.g., hospitalization to examine laboratory abnormalities that have persisted before the study until now);<br>• Scheduled treatment or surgery before the start of study unrelated to an AE that should be documented throughout the entire study protocol and/or in the subjects' individual baseline information;<br>• Hospitalization unrelated to an AE but merely for use of blood products.	Further detailed the description

Revision Location	Version 1.1	Version 2.0	Rationales for Changes
**10.2.1.5 Reporting of serious adverse events/Page 45**	SAEs that occur starting from the signing of the informed consent form until 30 days (inclusive) after the last dose should be reported. During this period, in the event of an SAE, whether it is an initial or follow-up report, the investigator must complete, sign, and date the "New Drug Clinical Study Serious Adverse Event (SAE) Report Form", report the event to the Clinical Medicine Department of sponsor Hengrui, CRA, medical director, and principal investigators by email, and submit the report to the leading site, ethics committee of the study site, China Food and Drug Administration (CFDA, now NMPA), as well as the investigator's local (provincial or city) food and drug administration by fax (the reporting method for CFDA is EMS) within 24 h of awareness. SAEs that occur 30 days after the last dose are generally not reported unless suspected to be related to the investigational drug.	SAEs that occur starting from the signing of the informed consent form until 30 days (inclusive) after the last dose should be reported. During this period, in the event of an SAE, whether it is an initial or follow-up report, the investigator must complete, sign, and date the "New Drug Clinical Study Serious Adverse Event (SAE) Report Form" immediately within 24 h of awareness, notify the sponsor within 24 h of knowing of the SAE, and submit the report to relevant authorities in time in accordance with regulatory requirements. If the investigator is aware of any SAE that occurs 30 days after the last dose, the investigator should report the event suspected to be related to the investigational drug to the sponsor immediately.<br><br>The email address for the sponsor to receive the report of SAE is: hengrui_drug_safety@hrglobe.cn	Revised the SAE reporting content, recipients, and procedures according to the latest version of GCP.
**10.2.1.5 Reporting of serious adverse events/Page 45**	Added contents	If an error is found in a previously reported SAE, such SAE may be revised, revoked, or downgraded in follow-up reports and reported in accordance with the SAE reporting procedure.	Revised the SAE reporting content, recipients, and procedures according to the latest version of GCP.

Revision Location	Version 1.1	Version 2.0	Rationales for Changes
**10.2.1.6 Suspected unexpected serious adverse reaction (SUSAR)/ Page 46**	Added contents	The suspected unexpected serious adverse reaction (SUSAR) refers to any serious adverse reaction that is suspected and unexpected, and whose nature and severity of clinical manifestations exceed the existing information such as the Investigator's Brochure of the investigational drug, the package insert of the marketed drug, or the summary of product characteristics.<br>The sponsor should immediately report SUSARs to regulatory authorities, investigators participating in the clinical study, and relevant institutions according to regulatory requirements. After receiving the SUSAR report, the investigator should timely receive and read it and report the event to the ethics committee.	Added the definition and submission procedure of SUSAR according to the latest GCP requirements
**13.2 Statistical Analysis/Page 52**	All statistical analyses will be performed using SAS 9.4. All statistical tests will adopt 2-sided tests. $P \leq 0.05$ is considered statistically significant, and 95% confidence is used for confidence interval.	All statistical analyses will be performed using SAS 9.4. In addition to the primary endpoint, all statistical tests will adopt 2-sided tests. $P \leq 0.05$ is considered statistically significant, and 95% confidence is used for confidence interval. According to the O'Brien & Fleming type α spending function, the final analysis of primary endpoint will be conducted using 95.4% confidence. The corresponding *P* value less than 0.046 (two-sided) will be considered statistically significant.	Revised corresponding description of the primary efficacy endpoint after adding interim analysis
**13.2.1 Efficacy analysis/Page 53**	For the primary efficacy endpoint OS, the final analysis will be performed when 252 deaths (≥ 80% of subjects die) are collected in this study.	For the primary efficacy endpoint OS, the final analysis will be performed when 253 deaths (≥ 80% of subjects die) are collected in this study.	Updated the number of death events according to the calculation results of sample size after adding interim analysis

<table>
<tr><th>Revision Location</th><th>Version 1.1</th><th>Version 2.0</th><th>Rationales for Changes</th></tr>
<tr><td>13.2.3 Interim analysis/Page 55</td><td>Added contents</td><td>One interim analysis is planned in this study. When the number of endpoint events reaches 70% (177), the unblinded SC will conduct the efficacy and safety evaluation on the unblinded data and provide recommendation on whether to submit application for registration in advance. The superiority boundaries determined using the O'Brien & Fleming type α spending function for the interim analysis are as follows:

Table 13.1. Superiority boundaries in the interim analysis and final analysis
<table><tr><th>Time Point</th><th>Number of Endpoint (OS) Events</th><th>Superiority Boundary Z-Value (Corresponding HR)</th><th>One-Sided Nominal Significance Level of Superiority</th></tr><tr><td>Interim Analysis</td><td>177 (70%)</td><td>-2.438 (HR = 0.692)</td><td>0.007</td></tr><tr><td>Final Analysis</td><td>253</td><td>-2.000 (HR = 0.777)</td><td>0.023</td></tr></table>
Note: OS = Overall Survival;

The interim analyses will be completed by unblinded statisticians and their programming team. The interim analysis results will be reviewed by the unblinded SC, and the SC will determine whether to put forward the proposal of application for registration in advance to the sponsor according to the results. If the sponsor applies for registration in advance, the early unblinding will be conducted at the same time (see 12.4 for details).</td><td>Added corresponding description of the interim analysis</td></tr>
</table>

Clinical Medicine Department II

Jiangsu Hengrui Pharmaceuticals Co., Ltd.

1 Feb., 2021

A RANDOMIZED, DOUBLE-BLIND, SINGLE-DUMMY, PARALLEL-CONTROLLED, MULTICENTER CLINICAL STUDY OF IRINOTECAN HYDROCHLORIDE LIPOSOME IN COMBINATION WITH 5-FU/LV AS SECOND-LINE TREATMENT FOR LOCALLY ADVANCED OR METASTATIC PANCREATIC CANCER AFTER TREATMENT FAILURE WITH GEMCITABINE-BASED THERAPY

STATISTICAL ANALYSIS PLAN

(V1.1)

Sponsor: Jiangsu Hengrui Pharmaceuticals Co., Ltd.

Statistical Institution: Department of Biostatistics, School of Public Health, Nanjing Medical University

Feb. 2022

Statistical Analysis Plan

(V1.1)

Investigational Drug:	Irinotecan Hydrochloride Liposome
Study Title:	A Randomized, Double-Blind, Single-Dummy, Parallel-Controlled, Multicenter Clinical Study of Irinotecan Hydrochloride Liposome in Combination with 5-FU/LV as Second-Line Treatment for Locally Advanced or Metastatic Pancreatic Cancer After Treatment Failure with Gemcitabine-Based Therapy
Study Phase:	Confirmatory clinical study
Sponsor:	Jiangsu Hengrui Pharmaceuticals Co., Ltd.
Principal Investigators:	The 81st Hospital of the Chinese People's Liberation Army Shukui Qin<br>Renji Hospital, Shanghai Jiaotong University School of Medicine Liwei Wang
Protocol Version Date:	Version: 2.0; Date: 28 Jan., 2021

Department of Biostatistics, School of Public Health, Nanjing Medical University

Version History

SAP Version and Date	Protocol Version and Date	Drafted by/Reviewed by	Revision Records
V0.1, 21 Dec., 2020	V1.1, 25 Oct., 2019	Yuanping Yue/Donghua Lou	Initial draft
V0.2, 31 Mar., 2021	V2.0, 28 Jan., 2021	Yuanping Yue/Donghua Lou	Updated according to protocol 2.0 and the feedback from sponsor
V1.0, 25 Jun., 2021	V2.0, 28 Jan., 2021	Yuanping Yue/Donghua Lou	Finalization
V1.1 draft, 15 Dec., 2021	V2.0, 28 Jan., 2021	Yuanping Yue/Donghua Lou	Revised according to the medical feedback
V1.1 draft, 18 Feb., 2022	V2.0, 28 Jan., 2021	Yuanping Yue/Donghua Lou	Revised according to the medical feedback and the discussion at the data review meeting
V1.1, 18 Feb., 2022	V2.0, 28 Jan., 2021	Yuanping Yue/Donghua Lou	Finalization

Signature Page of Statistical Analysis Plan

Study Title: A Randomized, Double-Blind, Single-Dummy, Parallel-Controlled, Multicenter Clinical Study of Irinotecan Hydrochloride Liposome in Combination with 5-FU/LV as Second-Line Treatment for Locally Advanced or Metastatic Pancreatic Cancer After Treatment Failure with Gemcitabine-Based Therapy

Sponsor: Jiangsu Hengrui Pharmaceuticals Co., Ltd.

Signature______________________ Date_____________________

Statistical Institution: Department of Biostatistics, School of Public Health, Nanjing Medical University

Author_______________________ Date_____________________

Reviewer_____________________ Date_____________________

TABLE OF CONTENTS

1. ABBREVIATIONS 1

2. PROTOCOL SYNOPSIS 2

2.1 Study Objectives 2

2.2 Study Population 2

2.3 Study Design 2

2.4 Sample Size Estimation 3

2.5 Randomization Method 4

2.6 Blinding 4

2.7 Interim Analysis 5

2.8 Modifications to the Content of Statistical Analysis in the Protocol 5

3. EFFICACY ENDPOINTS 6

3.1 Primary Efficacy Endpoint 6

3.2 Secondary Efficacy Endpoints 6

3.3 Response Evaluation Criteria 7

4. SAFETY ENDPOINTS 8

4.1 Adverse Events 8

4.2 Treatment-Related Adverse Events 9

4.3 Serious Adverse Events 9

4.4 Vital Signs and Physical Examinations 11

4.5 Laboratory Tests 11

4.6 12-Lead ECG 12

4.7 Body Weight 12

4.8 Pain Score 12

4.9 ECOG PS 12

5. OTHER EVALUATION ENDPOINTS 12

6. ANALYSIS SETS 12

6.1 Intention-to-Treat (ITT) Set 12

6.2 Safety Set (SS) 12

6.3 Per-Protocol Set (PPS) 12
6.4 Evaluable Patient for Tumor Response (EP) 13
6.5 Evaluable Patient for Tumor Markers 13

7. STATISTICAL ANALYSIS 13

7.1 General Principles 13
7.1.1 Significance level 13
7.1.2 Hypothesis test 13
7.1.3 Estimation of missing values 14
7.1.4 Data protocol 14
7.2 Study Population 16
7.2.1 Subject disposition 16
7.2.2 Protocol violation and deviation 16
7.2.3 Analysis of subject characteristics 16
7.3 Efficacy Evaluation 17
7.3.1 Analysis of primary efficacy endpoint 17
7.3.2 Analysis of secondary efficacy endpoints 19
7.4 Safety Evaluation 21
7.4.1 Drug exposure and dose modification 21
7.4.2 Adverse events 21
7.4.3 Vital signs 22
7.4.4 Laboratory test parameters 22
7.4.5 ECG 22
7.4.6 Physical examination 22
7.4.7 Body weight 23
7.4.8 Pain score 23
7.4.9 ECOG PS 23
7.4.10 Concomitant medication 23
7.5 Interim Analysis 23
7.6 Subgroup Analysis 23
7.7 Sensitivity Analysis 24
7.7.1 Secondary efficacy endpoint PFS 24

7.7.2 Primary efficacy endpoint OS 25
7.8 Other Analyses 25
8. STATISTICAL ANALYSIS SOFTWARE 25
9. REFERENCES 25
10. LIST OF PROTOCOL DEVIATIONS 26
11. ATTACHED TABLES 31

1. ABBREVIATIONS

AE	Adverse Event
CI	Confidence Interval
CR	Complete Response
NCI CTC AE	National Cancer Institute Common Terminology Criteria for Adverse Event
ECOG	Eastern Cooperative Oncology Group
ITT	Intention-To-Treat
Max	Maximum
Mean	Mean
MedDRA	Medical Dictionary for Regulatory Activities
Median	Median
Min	Minimum
ORR	Objective Response Rate
OS	Overall Survival
PD	Progressive Disease
PFS	Progression-Free Survival
PPS	Per-Protocol Set
PR	Partial Response
PT	Preferred Term
Q_1	25th Percentile
Q_3	75th Percentile
EP	Evaluable Patient for Tumor Response
SC	Steering Committee
SAE	Serious Adverse Event
SD	Stable Disease
SOC	System Organ Class
Std	Standard Deviation
SS	Safety Set
TEAE	Treatment-Emergent Adverse Event
TTF	Time to Treatment Failure

2. PROTOCOL SYNOPSIS

2.1 Study Objectives

- Primary objective

  To compare the overall survival (OS) of experimental group of irinotecan hydrochloride liposome in combination with 5-FU/LV vs. control group of 5-FU/LV.

- Secondary objective

  To compare the following endpoints of experimental group vs. control group:

  - ✓ Progression-free survival (PFS)
  - ✓ Time to treatment failure (TTF)
  - ✓ Objective response rate (ORR)
  - ✓ CA19-9 tumor marker response
  - ✓ Quality of life score (EORTC-QTQ-C30)
  - ✓ Safety evaluation: adverse events (AEs), including clinical symptoms and signs and laboratory tests, serious adverse events (SAEs)

2.2 Study Population

Patients with locally advanced or metastatic pancreatic cancer who have failed gemcitabine-based therapy.

2.3 Study Design

This is a randomized, double-blind, single-dummy, parallel-controlled, multicenter clinical study to compare the efficacy and safety of irinotecan hydrochloride liposome in combination with 5-FU/LV vs. placebo in combination with 5-FU/LV as second-line treatment for locally advanced or metastatic pancreatic cancer after treatment failure with gemcitabine-based therapy. Approximately 272 eligible subjects will be randomized in a 1:1 ratio to either the experimental group (irinotecan liposome in combination with 5-FU/LV) or the control group (placebo in combination with 5-FU/LV), and will be randomly stratified according to the following stratification factors:

(1) Albumin level (≥ 40 g/L vs. < 40 g/L);

(2) History of fluorouracil therapy (with vs. without);

(3) History of gemcitabine therapy (gemcitabine alone vs. gemcitabine combination).

Subjects will be treated according to the randomized dosing regimen until progressive disease (PD) (radiographic or clinical deterioration) or intolerable toxicity occurs. After treatment discontinuation, subjects will undergo a 30-day follow-up period. Afterwards, subjects will be followed up once a month by telephone or other means. Subjects' survival status will be recorded until death or study closure (whichever occurs first). The primary endpoint will be analyzed after approximately 253 deaths occur. The entire study is planned to be completed in 36 months.

In this study, blinded and unblinded steering committees (SCs) are planned to be set up, and an interim analysis will be conducted. After data review, the unblinded SC will provide recommendation on whether to submit application for registration in advance. See SC Charter for detailed information on composition, responsibilities, and procedures of SC.

2.4 Sample Size Estimation

In this study, placebo + 5-FU/LV will be used as the control and a superiority test will be performed between the two groups, with OS as the primary efficacy endpoint for sample size estimation.

With reference to the original NAPOLI-1 study data for pancreatic cancer (the OS was 6.1 months in the experimental group and 4.2 months in the control group) and the current status of pancreatic cancer in China, the median OS of the experimental group and the control group in this study is set as 5.0 months and 3.5 months, respectively, with significance level α = 0.025 (one-sided), a power of 80%, the enrollment duration of 24 months, the total study duration of 36 months, and a dropout rate of 20%. A design of experimental group:placebo group = 1:1 will be adopted. An interim analysis is planned when 70% of OS events are collected to determine whether to conduct application for registration in advance. Based on the above parameters, at least 253 deaths should be collected and a total of 272 subjects should be enrolled (136 subjects in the experimental group and 136 subjects in the control group) according to the log-rank test for the OS comparison between the two groups and the O'Brien & Fleming type α spending function (EAST 6.5).

2.5 Randomization Method

Subjects will be allocated by central randomization and enrolled competitively at all sites. The central randomization procedure will be performed using the central randomization system of Nanjing Medical University. After the investigators of each study site participating in this study screen out each eligible subject, they should fill in screening data, obtain randomization numbers, and log into the drug management system and dispense the corresponding study drugs according to randomization numbers.

Subjects will be subjected to stratified randomization based on the following factors at baseline:

(1) Albumin level (< 40 g/L vs. ≥ 40 g/L);

(2) History of fluorouracil therapy (with vs. without);

(3) History of gemcitabine therapy (gemcitabine alone vs. gemcitabine combination).

2.6 Blinding

The placebo will be provided by the sponsor and should be identical to the investigational drug in appearance. Neither the investigator nor the subjects know the allocation of study treatment. The randomization number is generated by SAS and imported into the central randomization system provided by the Department of Biostatistics, School of Public Health, Nanjing Medical University. The blind code information will be saved as electronic information. When the investigators apply for unblinding, by the consent of the sponsor and the leading site, they can log into the electronic information system to obtain the blind code information (the information on the assignment of the subjects to the experimental group or the control group).

Unblinded SC will be unblinded at the interim analysis. The interim analysis will be conducted by an unblinded statistician to ensure that the sponsor, investigators, and subjects remain blinded until the primary analysis of the study (253 death events).

2.7 Interim Analysis

The sponsor received the approval letter for protocol (V2.0) amendment from the CDE on 17 Nov., 2021, in which the additional interim analysis in protocol V2.0 was approved. Compared with the scheduled time point for interim analysis in protocol V2.0 (when 70% of OS events [177] are collected), this study has reached the requirements of interim analysis as of 9 Jul., 2021 (more than 70% of OS events were collected and 188 OS events were actually collected). The data cutoff date for the interim analysis was set as 9 Jul., 2021 (inclusive) by the sponsor. According to the 188 events actually collected, the project statistician adjusted the nominal significance level of the interim analysis accordingly. The adjusted nominal significance level of the interim analysis was calculated as 0.009 (one-sided) by EAST software (version 6.5) and the nominal significance level of the final analysis was 0.022 (one-sided).

The interim analyses will be completed by unblinded statisticians and their programming team. The interim analysis results will be reviewed by the unblinded SC, and the SC will determine whether to put forward the proposal of application for registration in advance to the sponsor according to the results.

2.8 Modifications to the Content of Statistical Analysis in the Protocol

2.8.1 The "full analysis set (FAS)" was modified to "intention-to-treat set (ITT)" and the description of FAS analysis was also replaced with that of ITT set.

2.8.2 The SC meeting was held on 16 Feb., 2022. The SC suggested that the interim analysis results reached the preset endpoint, and the application for registration can be carried out in advance. As of 18 Nov., 2021, a total of 228 OS events were collected. The significance level was adjusted based on the 228 OS events of the interim analysis and the final 253 OS events accordingly. The adjusted nominal significance level of the interim analysis was calculated as 0.018 (one-sided) by EAST software (version 6.5) and the nominal significance level of the final analysis was 0.020 (one-sided).

2.8.3 "In the analysis of the primary efficacy endpoint, the survival functions of the experimental group and the control group will be estimated by the Kaplan-Meier method, and the survival curve will be plotted. The survival functions of the two groups will be compared by the unstratified log-rank test. In addition, if the proportional hazard assumption is established between the two groups, a Cox model can be used to estimate the hazard ratio (HR) between the two groups and its overall 95% confidence interval (CI) will be calculated. For the secondary

analysis of the primary efficacy endpoint OS, the hazard ratio (HR) and its 95% CI will be estimated, taking into account important covariates such as albumin level (< 40 g/L vs. ≥ 40 g/L), history of fluorouracil therapy (with vs. without), and history of gemcitabine therapy (gemcitabine alone vs. gemcitabine combination)." was changed to "For the primary analysis of the primary efficacy endpoint OS, the survival functions of the experimental group and the control group will be estimated by the Kaplan-Meier method, and the survival curve will be plotted. The survival functions of the two groups will be compared using the stratified log-rank test. The stratification factors are albumin level (< 40 g/L vs. ≥ 40 g/L), history of fluorouracil therapy (with vs. without), and history of gemcitabine therapy (gemcitabine alone vs. gemcitabine combination). In addition, the Cox proportional hazards regression model will be used to estimate the hazard ratio (HR) between the two groups and its overall 96.4% CI will be calculated. The HR and its 96.4% CI will be estimated, taking into account important covariates such as albumin level (< 40 g/L vs. ≥ 40 g/L), history of fluorouracil therapy (with vs. without), and history of gemcitabine therapy (gemcitabine alone vs. gemcitabine combination).

For the secondary analysis of the primary efficacy endpoint OS, the OS of the two groups will be compared using the unstratified log-rank test, and the Cox proportional hazards regression model will be used to estimate the HR and its 96.4% CI."

2.8.4 "Grade 3-4 AEs" in Section 13.2.2 Safety analysis of the protocol was changed to "Grade ≥ 3 AEs"

3. EFFICACY ENDPOINTS

3.1 Primary Efficacy Endpoint

Overall survival (OS): Defined as the time from randomization to death due to any cause.

For subjects who are still alive at the end of the study, the time of the end of study is used as the censoring date of their survival. For subjects who are lost to follow-up, the time of the last visit is used as the censoring date of their survival.

3.2 Secondary Efficacy Endpoints

- Progression-free survival (PFS)

Defined as the time from the date of randomization to the date of documented tumor progression or death due to any cause.

The PFS is assessed as per RECIST V1.1. The analysis of this endpoint includes all tumor evaluations during the treatment period and the follow-up period. If the subject has several endpoints that can be evaluated as progressive disease (PD), such as relapse, new lesions, or death, then the first documented endpoint will be used to analyze PFS; if the subject uses other treatment regimens or anti-tumor treatment against the target lesion, it is also taken as censoring data.

- Time to treatment failure (TTF)

TTF is defined as the time from randomization to PD, death, or study discontinuation due to toxicities, including withdrawal of ICF by subjects.

- Objective response rate (ORR)

The proportion of subjects with a best response of CR or PR (through 4-week efficacy confirmation) during the study. Objective response is evaluated based on the Response Evaluation Criteria in Solid Tumors (RECIST) V1.1. Subjects must have measurable lesions at baseline. These lesions can be assessed as complete response (CR), partial response (PR), stable disease (SD), and progressive disease (PD) according to RECIST V1.1.

- Change in CA19-9 tumor marker response

The tumor marker response rate is assessed based on changes in CA19-9 serum concentration. Tumor marker response is defined as at least one decrease in concentration from baseline by at least 50% during treatment. Only subjects with baseline values > 30 U/mL will be included in the evaluation of tumor marker response rates.

- Quality of life score (QoL) (with reference to EORTC QLQ-C30)

3.3 Response Evaluation Criteria

According to the RECIST V1.1, the response is evaluated as complete response (CR), partial response (PR), stable disease (SD), and progressive disease (PD). The evaluation will be performed by the investigator.

4. SAFETY ENDPOINTS

4.1 Adverse Events

An adverse event refers to any untoward medical occurrence in a study subject administered a pharmaceutical product. Any untoward medical occurrence after the subject receives the investigational drug until 30 days after the end of treatment is judged as an AE, regardless of the causality with the investigational drug. For abnormalities of the physical examination or laboratory tests that are already present during the baseline period, they are also judged as AEs if the severity of these abnormalities is increased after the subjects receive the drug treatment.

AEs are found by asking subjects non-leading questions at each visit during the study. In addition, AEs are also found by subjects' reporting during or between visits, or by physical examinations, laboratory tests, or other evaluations.

The investigator should record in detail any AEs occurring in subjects and evaluate each AE (causality with the investigational drug, whether they are SAEs, etc.). The following information on AEs should be recorded:

(1) Description of all relevant symptoms

(2) Onset time

(3) Severity (graded as per CTCAE V4.03)

(4) Action (e.g., treatment continuation, dose reduction, treatment resumption after interruption, and treatment discontinuation)

(5) Whether they are SAEs

(6) Causality with the investigational drug (related, possibly related, unlikely related, not related, and unassessable)

(7) Duration

(8) Outcomes of AEs (recovered/resolved; recovered with sequelae; alleviated; persisted; worsened; death; unknown)

Once the subject stops treatment, the toxicities will be evaluated within 30 days after the last dose if necessary, until all treatment-related toxicities resolve to the baseline level (or Grade ≤ 1 as per CTCAE V4.0), become stable, or are considered irreversible.

4.2 Treatment-Related Adverse Events

Based on the causality assessment criteria, the relationship between AEs and investigational drug is classified into 5 categories: related, possibly related, unlikely related, not related, and unassessable. Events that are assessed to be related, possibly related, and unassessable will be listed as treatment-related adverse events (TRAEs). The incidence of TRAEs will be calculated using the total number of subjects with TRAEs as the numerator and the total number of subjects included in the AE evaluation as the denominator.

4.3 Serious Adverse Events

- Definition of serious adverse event

  A serious adverse event (SAE) refers to a medical occurrence during the clinical trial that results in hospitalization, prolonged hospitalization, disability, incapacity, threats to life or death, or congenital malformation. The following unexpected medical events are included:

  - ✓ Events leading to death;
  - ✓ Life-threatening events (defined as events where the subject is at immediate risk of death at the time of the onset);
  - ✓ Events leading to hospitalization or prolonged hospitalization;
  - ✓ Events leading to permanent or serious disability/incapacity;
  - ✓ Events leading to congenital anomalies or birth defects.

- Pregnancy

  For pregnancy occurring during the clinical study, the investigator should report the pregnancy to the sponsor within 24 h of knowing the event by filling out the "Hengrui Clinical Study Pregnancy Report/Follow-up Form".

  The investigators should track a pregnancy event until its final outcomes (including any premature termination of pregnancy or childbirth), and childbirth should be followed up for 1 month after childbirth. The pregnancy outcomes should be reported to the sponsor. If pregnancy outcomes meet the SAE criteria (such as ectopic pregnancy, spontaneous abortion, intrauterine death, neonatal death, or congenital anomalies, etc.), they must be reported according to SAE procedures.

If a subject experiences any SAE during pregnancy, the SAE should be reported according to the SAE reporting procedure.

- Progressive disease

PD (including progressive signs and symptoms) is not necessarily reported as an SAE. However, death due to PD during the study or safety reporting period should be reported as an SAE. Hospitalizations due to signs and symptoms of PD should not be reported as an SAE. If a subject dies during administration or within 30 days after the last dose, the event leading to the death must be reported as an SAE.

- Other anti-tumor treatments

If a subject is to start another anti-tumor treatment, AEs except death will be reported until 30 days after the last dose of the study drugs. Death that occurs within the SAE reporting period after the end of study treatment should be reported regardless of whether the subjects receive other treatments.

- Hospitalization

During the study, AEs that lead to hospitalization or prolonged hospitalization should be considered as SAEs. Any initial hospital admission by a medical facility meets this criterion (even if less than 24 hours).

Hospitalization does not include: rehabilitation institution; sanatorium; general emergency admission; day surgery (outpatient/same-day/ambulatory surgery).

Hospitalization or prolonged hospitalization not related to the worsening of AEs is not considered an SAE. For example:

- ✓ Hospitalization due to the pre-existing disease without new AEs and aggravation of the pre-existing disease (e.g., hospitalization to examine laboratory abnormalities that have persisted before the study until now);
- ✓ Hospitalization for management reasons (e.g., annual physical examination);
- ✓ Hospitalization during the study as specified in the study protocol (e.g., as required by the protocol);
- ✓ Elective hospitalization unrelated to the deterioration of an AE (e.g., elective cosmetic surgery);

- ✓ Scheduled treatment or surgery that should be documented throughout the entire study protocol and/or in the subjects' individual baseline information;
- ✓ Hospitalization merely for use of blood products.

Diagnostic or therapeutic invasive (e.g., surgery) or non-invasive procedures should not be reported as AEs. However, the disease condition leading to such procedures should be reported if it meets the definition of an AE. For example, acute appendicitis during the AE reporting period should be reported as an AE and the appendectomy should be documented as the treatment method for the event.

4.4 Vital Signs and Physical Examinations

- Vital signs: pulse (beats/min), respiratory rate (resp/min), body temperature (°C), and blood pressure (mmHg);
- Physical examination: general conditions, skin, oral cavity, eyes, ears, nose, throat, lymph nodes, head and neck, respiratory system, cardiovascular system, abdomen, nervous system, and mental state. Particular attention should be paid to the sites of tumor lesions;

4.5 Laboratory Tests

- Hematology: white blood cell count (WBC), red blood cell count (RBC), hemoglobin (Hb), platelet count (PLT), neutrophil count (NEU), and lymphocyte count (LYM);
- Urinalysis: urine pH, urine protein, urine glucose, urine ketone body, microscopic urine red blood cells, and microscopic urine white blood cells;
- Blood biochemistry: liver function [serum alanine aminotransferase (ALT), aspartate aminotransferase (AST), total protein (TP), albumin (ALB), prealbumin, globulin, total bilirubin (TBil), direct bilirubin (DBil), alkaline phosphatase (ALP), gamma-glutamyl transferase (GGT), lactate dehydrogenase (LDH), and C-reactive protein (CRP)], kidney function [serum creatinine (Cr), blood urea nitrogen (BUN), and creatinine clearance], electrolytes [serum potassium ion (K^+), sodium ion (Na^+), chloride ion (Cl^-), calcium ion (Ca^{2+}), magnesium ion (Mg^{2+}), and phosphorus (P)], and blood lipid and blood glucose [triglyceride (TG), total cholesterol (TC), low-density lipoprotein (LDL), high-density lipoprotein (HDL), and blood glucose (GLU)];
- Coagulation function: prothrombin time (PT), activated partial thromboplastin time (APTT), international normalized ratio (INR), fibrinogen (FIB), thrombin time (TT), and D-dimer;

4.6 12-Lead ECG

- Heart rate, PR interval, QT interval, and QTc interval;

4.7 Body Weight

4.8 Pain Score

4.9 ECOG PS

5. OTHER EVALUATION ENDPOINTS

No

6. ANALYSIS SETS

The analysis sets of this study include intention-to-treat (ITT) set, safety set (SS), per-protocol set (PPS), evaluable patient for tumor response (EP), and evaluable patient for tumor markers.

6.1 Intention-to-Treat (ITT) Set

According to the ITT principle, all subjects who have signed the informed consent form and have been randomized, regardless of whether they have taken drugs, constitute the ITT set of this study. The division of this population is based on the number of randomized subjects from the randomization system. This dataset is mainly used for the baseline and efficacy analysis.

6.2 Safety Set (SS)

The SS is a subset of the ITT set and includes subjects who have received at least one dose of treatment. This set is used for safety analysis. All analyses conducted in this set are based on the actual treatment intensity.

6.3 Per-Protocol Set (PPS)

The PPS is a subset of the ITT set. All subjects who meet the protocol-specified criteria, have good compliance, and have received the study treatment for at least 6 weeks constitute the PPS of this study.

The PPS will be used for the per protocol analysis of the primary efficacy endpoint and critical secondary efficacy endpoints. Before database locking, the principal investigator, statistician and sponsor should determine the final PPS during the data review meeting.

6.4 Evaluable Patient for Tumor Response (EP)

Refer to all patients who have been randomized and received treatment, meet the inclusion/exclusion criteria of the study protocol, have measurable target lesions, and have undergone tumor evaluation at least once during treatment, except for patients with early PD, such as symptom aggravation and tumor-induced death.

6.5 Evaluable Patient for Tumor Markers

Refer to patients with CA19-9 level > 30 U/mL at baseline.

7. STATISTICAL ANALYSIS

7.1 General Principles

For continuous variables, the number of non-missing subjects, mean, standard deviation, median, minimum, and maximum should be listed. The decimal places of minimum and maximum should be consistent with those of the records in the database. The mean, median, and standard deviation will retain one more decimal place than the raw data recorded in the database.

For categorical variables, a frequency table (frequency and percentage) will be used. Percentages retain one decimal place.

7.1.1 Significance level

In addition to the primary endpoint, all statistical analyses will be performed using two-sided tests. $P \leq 0.05$ is considered statistically significant, and 95% confidence is used for confidence intervals. As of 18 Nov., a total of 228 OS events have been collected. According to the O'Brien & Fleming type α spending function, the primary endpoint will be analyzed using 96.4% confidence. The corresponding *P* value less than 0.036 (two-sided) will be considered statistically significant.

7.1.2 Hypothesis test

The primary efficacy endpoint for this study is OS.

The log-rank test is used for the comparison between the experimental group and the control group:

Hypothesis:

H_0: The survival process of the experimental group is the same as that of the control group;

H_1: The survival process of the experimental group is different from that of the control group.

α level: 0.036 (two-side).

7.1.3 Estimation of missing values

The missing data of efficacy endpoints OS, PFS, and TTF will be taken as censoring data and the censoring rules are shown in Table 1;

In the tumor efficacy evaluation data, if the data of efficacy endpoint is missing, the subject will be imputed as a non-responder when calculating ORR.

The missing values will not be estimated in the safety evaluation.

7.1.4 Data protocol

1) **Missing dates:**

If a previous date is incomplete, which affects the calculation of subsequent dates, the previous date will be imputed as follows in the absence of contradiction with other dates:

➢ **Dates related to survival**

✓ Missing year, month, and day of the death date: The death date will be imputed with the last visit date (subject is still alive);

✓ Missing day and month of the death date: If the "year" of the death date is the same as the "year" of the last visit date, the death date will be imputed with the last visit date. If the "year" of the death date is greater than the "year" of the last visit date, the death date will be imputed with 1 Jan.

✓ Missing day of the death date: If the "year and month" of the death date are the same as the "year and month" of the last visit date, the death date will be imputed with the last visit date. If the "year and month" of the death date are greater than the "year and month" of the last visit date, the death date will be imputed with the 1st day.

➢ **Dates related to adverse events**

✓ Missing year, month, and day of the onset date of adverse event: The date will not be imputed but presented as missing;

✓ Missing day and month of the onset date of adverse event: If the "year" of the onset date is the same as the "year" of the date of first dose, the onset date will be imputed

with date of first dose; if the "year" of the onset date is not the same as the "year" of the date of first dose, the onset date will be imputed with 1 Jan.

- ✓ Missing day of the onset date of adverse event: If the "year and month" of the onset date of adverse event are the same as the "year and month" of the date of first dose, the onset date will be imputed with the date of first dose; if the "year and month" of the onset date of adverse event are not the same as the "year and month" of the date of first dose, the onset date will be imputed with the 1st day.
- ✓ Missing year, month, and day of the end date of adverse event: The end date will not be imputed but presented as missing.
- ✓ Missing day and month of the end date of adverse event: The end date will be imputed with 31 Dec.
- ✓ Missing day of the end date of adverse event: The end date will be imputed with the last day of the month.

➢ **Dates related to concomitant medications**

- ✓ Missing year, month, and day of the start date of concomitant medication: The date will not be imputed but presented as missing;
- ✓ Missing day and month of the start date of concomitant medication: If the "year" of the start date is the same as the "year" of the date of first dose, the start date will be imputed with date of first dose; if the "year" of the start date is not the same as the "year" of the date of first dose, the start date will be imputed with 1 Jan.
- ✓ Missing day of the start date of concomitant medication: If the "year and month" of the start date are the same as the "year and month" of the date of first dose, the start date will be imputed with date of first dose; if the "year and month" of the start date are not the same as the "year and month" of the date of first dose, the start date will be imputed with the 1st day.
- ✓ Missing year, month, and day of the end date of concomitant medication: The end date will not be imputed and the medication is considered ongoing.
- ✓ Missing day and month of the end date of concomitant medication: The end date will be imputed with 31 Dec.
- ✓ Missing day of the end date of concomitant medication: The end date will be imputed with the last day of the month.

2) **Definition of baseline:** The baseline is defined as the last non-missing measurement before the first dose. If a subject has received the investigational drug but the date of first dose is missing, the baseline will be defined as the last non-missing measurement before the enrollment.

3) **Change from baseline:** Defined as the measured value in post-treatment follow-up − the measured value at baseline.

4) **Derivation and conversion of data:**

Age (unit: year) = (date of informed consent − date of birth)/365.25, rounded down.

Body mass index = body weight (kg)/height^2 (m^2), rounded to 1 decimal place.

Disease course (unit: month) = (date of informed consent − date of initial pathological diagnosis)/30.4375, rounded to 1 decimal place.

Date of last progression (unit: month) = (date of randomization − date of last progression/recurrence)/30.4375, rounded to 1 decimal place.

Calculation formula of survival (month): (event date (or censoring date) − date of randomization + 1 day)/30.4375, rounded to 1 decimal place.

7.2 Study Population

7.2.1 Subject disposition

Describe the subject enrollment and premature discontinuation at each site: number of subjects;

Disposition of subjects in each data set;

Subjects who prematurely discontinue treatment will be described one by one: status of drug administration, reason for early withdrawal from study, etc.

7.2.2 Protocol violation and deviation

Summarize and describe the cases of protocol violations and deviations. The definitions of major and minor protocol violations are shown in Section 10.

7.2.3 Analysis of subject characteristics

Age: Described using mean, standard deviation, maximum, minimum, and median.

Gender and ethnicity: The frequency and proportion will be calculated.

Height, weight, and body mass index: Described using mean, standard deviation, maximum, minimum, and median.

Tumor history: The course of disease will be described using mean, standard deviation, maximum, minimum, and median; for histological classification, primary lesion, clinical staging before enrollment, TNM staging before enrollment, and presence of metastases, the frequency and proportion will be calculated.

Cancer surgery history, history of chemotherapy, history of targeted therapy, history of radiotherapy, history of biotherapy, history of allergy, medical history, and medication history: The frequency and proportion will be calculated.

Hepatitis B virus test and genomics examination: The frequency and proportion will be calculated.

Randomization stratification factors: albumin level (< 40 g/L vs. ≥ 40 g/L); history of fluorouracil therapy (with vs. without); history of gemcitabine therapy (gemcitabine alone vs. gemcitabine combination): The frequency and proportion will be calculated.

Baseline vital signs (pulse, respiratory rate, body temperature, and blood pressure): Described using mean, standard deviation, maximum, minimum, and median.

Baseline physical examination: The frequency and proportion will be calculated.

ECOG PS and QoL at baseline: Described using mean, standard deviation, maximum, minimum, and median.

The baseline analysis will be based on the ITT.

7.3 Efficacy Evaluation

7.3.1 Analysis of primary efficacy endpoint

The primary efficacy endpoint for this study is OS. The analysis will be performed based on the ITT and PPS.

The calculation formula of OS is detailed as follows:

OS (month) = (date of death due to any cause – date of randomization + 1)/30.4375

For the primary analysis of the primary efficacy endpoint OS, the survival functions of the experimental group and the control group will be estimated by the Kaplan-Meier method, and the survival curve will be plotted. The survival functions of the two groups will be compared using the stratified log-rank test. The stratification factors are albumin level (< 40 g/L vs. ≥ 40 g/L), history of fluorouracil therapy (with vs. without), and history of gemcitabine therapy (gemcitabine alone vs. gemcitabine combination). In addition, the Cox proportional hazards regression model will be used to estimate the hazard ratio (HR) between the two groups and its overall 96.4% CI will be calculated. The HR and its 96.4% CI will be estimated, taking into account important covariates such as albumin level (< 40 g/L vs. ≥ 40 g/L), history of fluorouracil therapy (with vs. without), and history of gemcitabine therapy (gemcitabine alone vs. gemcitabine combination).

For the secondary analysis of the primary efficacy endpoint OS, the OS of the two groups will be compared using the unstratified log-rank test, and the Cox proportional hazards regression model will be used to estimate the HR and its 96.4% CI.

The subgroup analysis of OS will be conducted according to stratification factors and prognostic factors.

Table 1. Censoring rules for survival analysis (endpoint)

Situation of Data	Result	Censoring Date
**Overall survival (OS)**		
No death information is collected after randomization but the follow-up information is available after randomization	Censoring	Finally learned survival date
No follow-up information is provided after randomization and there is no date of death	Censoring	Date of randomization
**Progression-Free Survival (PFS)**		
No baseline imaging evaluation	Censoring	Date of randomization
No post-baseline imaging evaluation (no death prior to the first scheduled imaging evaluation)	Censoring	Date of randomization
No PD or death during the study	Censoring	Date of last imaging evaluation
PD or death after missing two consecutive imaging evaluations	Censoring	Date of last imaging evaluation before missing
Treatment discontinuation due to AEs or other reasons, no evidence of subsequent progression*	Censoring	Date of last imaging evaluation
Start of other new anti-tumor treatments	Censoring	Date of last imaging evaluation before the start of new anti-tumor treatment

Situation of Data	Result	Censoring Date
**Time to Treatment Failure (TTF)**		
No treatment discontinuation at the end of the study	Censoring	Study termination date

* Other reasons include withdrawal of informed consent, protocol deviation, start of other anti-tumor treatments, lost to follow-up, intolerable toxicity, treatment discontinuation requested by the subject, investigator's decision, and study termination by the sponsor.

7.3.2 Analysis of secondary efficacy endpoints

➢ **Progression-free survival (PFS)s**

The calculation formula of PFS is detailed as follows:

PFS (month) = (date of PD or death (whichever occurs first) – date of randomization + 1)/30.4375

For the primary analysis of PFS, the PFS of the two groups will be compared using the stratified log-rank test. The stratification factors are albumin level (< 40 g/L vs. ≥ 40 g/L), history of fluorouracil therapy (with vs. without), and history of gemcitabine therapy (gemcitabine alone vs. gemcitabine combination). The median PFS will be estimated using the Kaplan-Meier method, its 95% CI will be calculated, and the Kaplan-Meier curve will be plotted. The Cox proportional hazards regression model will be adopted, and the HR and its 95% CI will be estimated, taking into account important covariates such as albumin level (< 40 g/L vs. ≥ 40 g/L), history of fluorouracil therapy (with vs. without), and history of gemcitabine therapy (gemcitabine alone vs. gemcitabine combination).

For the secondary analysis of PFS, the PFS of the two groups will be compared using the unstratified log-rank test, and the Cox proportional hazards regression model will be used to estimate the HR and its 95% CI.

The analysis for this part is based on the ITT and PPS.

➢ **Time to treatment failure (TTF)**

The calculation formula of TTF is detailed as follows:

TTF (month) = (date of PD, death, or treatment discontinuation due to toxicity or any cause (whichever occurs first) – date of randomization + 1)/30.4375

The TTF of the two groups will be compared using the stratified log-rank test. The stratification factors are albumin level (< 40 g/L vs. ≥ 40 g/L), history of fluorouracil therapy (with vs. without), and history of gemcitabine therapy (gemcitabine alone vs. gemcitabine combination). The median TTF will be estimated using the Kaplan-Meier method, its 95% CI will be calculated, and the Kaplan-Meier curve will be plotted. The Cox proportional hazards regression model will be adopted, and the HR and its 95% CI will be estimated, taking into account important covariates such as albumin level (< 40 g/L vs. ≥ 40 g/L), history of fluorouracil therapy (with vs. without), and history of gemcitabine therapy (gemcitabine alone vs. gemcitabine combination).

The analysis for this part is based on the ITT and PPS.

- **Objective response rate (ORR)**

ORR will be assessed as per RECIST V1.1.

The calculation formula of ORR is detailed as follows:

ORR (%) = (confirmed CR + confirmed PR)/total number of evaluated subjects × 100%

The ratio of the objective response subjects (confirmed CR + confirmed PR) to the total number of subjects (ORR) and its 95% CI will be calculated. The 95% CI of ORR will be calculated based on the exact probability method. The *Fisher's* exact test will be used to compare the ORR of the two groups and the 95% CI of rate difference will be calculated by the normal approximation method.

The analysis for this part is based on the ITT, PPS, and EP.

- **Analysis of tumor markers**

Serum CA19-9 will be determined within 7 days before administration, and every 6 weeks thereafter. The tumor marker response rate is assessed based on changes in CA19-9 serum concentration. Tumor marker response is defined as at least one decrease in concentration from baseline by at least 50% during treatment. Only subjects with baseline values > 30 U/mL will be included in the evaluation of tumor marker response rates.

The tumor marker response rate and its 95% CI will be calculated. The 95% CI will be calculated using the exact probability method. The inter-group comparison will be conducted by the *Fisher's* exact test and the 95% CI of rate difference will be calculated using the normal approximation method.

The analysis for this part is based on the evaluable patients for tumor markers.

- **Quality of life score (QoL)**

A linear transformation will be performed for QoL to standardize the raw score so that the standard score ranges from 0 to 100. Descriptive statistics and paired *t*-test will be performed on the results.

The analysis for this part is based on the ITT and PPS.

7.4 Safety Evaluation

The safety analysis is based on the SS.

7.4.1 Drug exposure and dose modification

The total actual dose and dose intensity of the two groups will be described using mean, standard deviation, maximum, minimum, and median.

The study treatment cycles and dose modification/discontinuation during treatment will be listed using frequency and percentage.

Dose intensity = total actual dose/(study treatment cycles × starting dose) × 100%

7.4.2 Adverse events

Treatment-emergent adverse event (TEAE) data will be coded according to the latest version of MedDRA at the time of coding and then processed in statistical analysis.

The number of events, number of subjects, and incidence of AEs/TRAEs, AEs/TRAEs leading to dose reduction, AEs/TRAEs leading to dose interruption, AEs/TRAEs leading to dose discontinuation, SAEs, treatment-related SAEs, Grade ≥ 3 AEs/TRAEs, AEs/TRAEs leading to withdrawal from study, and AEs/TRAEs leading to death will be summarized.

If the same subject experiences multiple AEs, the number of subjects will be counted as one when calculating the incidence of the AEs; if a subject experiences the same AE multiple times, the number of subjects will be counted as one when calculating the incidence of the AE.

AEs will be summarized in the frequency table based on System Organ Class (SOC) and Preferred Term (PT). The incidence will be calculated based on system and signs/symptoms (N = number of subjects with at least one occurrence for a given event).

The number of cases, number of subjects, and incidence of AEs at various severity levels as per NCI CTCAE v4.03 and with various causalities with the investigational drug will be calculated by SOC and PT (for AEs with multiple occurrences, the severity analysis of the number of subjects and the incidence will be based on the highest severity).

A list of subjects with AEs and SAEs will be provided.

7.4.3 Vital signs

Measurement values and their changes before and after treatment will be described using mean ± standard deviation, maximum, minimum, and median.

7.4.4 Laboratory test parameters

For hematology, blood biochemistry, and coagulation function, the measurement values and their changes before and after treatment will be described using mean ± standard deviation, maximum, minimum, and median. The shift table will be used to describe the normal and abnormal changes before and after treatment.

Urinalysis: The shift table will be used to describe the normal and abnormal changes before and after treatment.

The proportion of subjects with "clinically significant abnormalities" in subjects with abnormal changes should be provided, where the clinical significance of the abnormalities should be judged by the investigator.

7.4.5 ECG

For heart rate, PR interval, QT interval, and QTc interval, the measurement values and their changes before and after administration will be described using mean ± standard deviation, maximum, minimum, and median. The shift table will be used to describe the normal and abnormal changes before and after medication.

The proportion of subjects with "clinically significant abnormalities" in subjects with abnormal changes should be provided, where the clinical significance of the abnormalities should be judged by the investigator.

7.4.6 Physical examination

The shift table will be used to describe the normal and abnormal changes before and after treatment.

7.4.7 Body weight

Measurement values and their changes before and after treatment will be described using mean ± standard deviation, maximum, minimum, and median.

7.4.8 Pain score

Measurement values and their changes before and after treatment will be described using mean ± standard deviation, maximum, minimum, and median.

7.4.9 ECOG PS

The changes in ECOG PS at each follow-up visit from baseline will be described.

7.4.10 Concomitant medication

The use of concomitant medications during the study (including any changes to the concomitant medications used during the screening period, new concomitant medications added after screening, medications for treating AEs, and concomitant medications during the follow-up period) will be summarized along with the frequency of use of various drugs

7.5 Interim Analysis

As of 9 Jul., a total of 188 OS events have been collected, i.e., when 74.3% of OS events are collected, the interim analysis is conducted according to the SAP.

The SC meeting was held on 16 Feb., 2022. The SC suggested that the interim analysis results reached the preset endpoint, and the application for registration can be carried out in advance.

7.6 Subgroup Analysis

- Subgroup analyses will be performed for the primary efficacy endpoint OS based on the ITT and PPS according to the following factors, and the forest plot on HR will be produced:
  - ✓ Albumin level (< 40 g/L vs. ≥ 40 g/L)
  - ✓ History of fluorouracil therapy (with vs. without)
  - ✓ History of gemcitabine therapy (gemcitabine alone vs. gemcitabine combination)
  - ✓ Baseline ECOG PS (0 vs. 1)
  - ✓ Start of new anti-tumor treatment (yes vs. no)

- ✓ Type of new anti-tumor treatment (none vs. chemotherapy vs. target therapy vs. radiotherapy vs. surgery vs. others)
- ✓ Number of metastatic organs (< 2 vs. ≥ 2)
- ✓ Baseline CA19-9 (< 40 U/mL vs. ≥ 40 U /mL)
- ✓ Age (< 65 years vs. ≥ 65 years)
- ✓ Gender (male vs. female)
- ✓ History of radiotherapy (yes vs. no)
- ✓ Surgical history of pancreatic cancer (yes vs. no)
- ✓ Clinical stage at initial diagnosis (IV vs. others)
- ✓ Liver metastasis (yes vs. no)
- ✓ Site of pancreatic tumor (pancreatic head vs. others)
- ✓ BMI (≥ median vs. < median)
- ✓ Disease course (≥ median vs. < median)
- ✓ Date of last progression (≥ median vs. < median)

7.7 Sensitivity Analysis

7.7.1 Secondary efficacy endpoint PFS

The following censoring rules will be used to calculate PFS. The PFS of the two groups will be compared using the stratified log-rank test. The stratification factors are albumin level (< 40 g/L vs. ≥ 40 g/L), history of fluorouracil therapy (with vs. without), and history of gemcitabine therapy (gemcitabine alone vs. gemcitabine combination). The median PFS will be estimated using the Kaplan-Meier method, its 95% CI will be calculated, and the Kaplan-Meier curve will be plotted. The Cox proportional hazards regression model will be adopted, and the HR and its 95% CI will be estimated, taking into account important covariates such as albumin level (< 40 g/L vs. ≥ 40 g/L), history of fluorouracil therapy (with vs. without), and history of gemcitabine therapy (gemcitabine alone vs. gemcitabine combination).

The analysis for this part is based on the ITT and PPS.

**Progression-Free Survival (PFS)**	**Date of Progression**	**Result**
No baseline tumor evaluation	The day of randomization	Censoring
Radiographic evidence of unequivocal progression	Date of imaging examination	Progression
Clinical evidence of unequivocal progression	Date of visit or date of evidence discovery	Progression
No progression	Date of last measurable imaging examination	Censoring
Treatment discontinuation due to AEs or other reasons, no evidence of subsequent progression*	Date of treatment discontinuation	Censoring
Start of other new anti-tumor treatments	Start date of anti-tumor treatment	Censoring
Death before the first efficacy evaluation	Date of death	Progression
No radiographic evidence of progression at last evaluation, died before next evaluation	Date of death	Progression
Death after missing more than one follow-up visit	Date of death	Progression

* Other reasons include withdrawal of informed consent, protocol deviation, start of other anti-tumor treatments, lost to follow-up, intolerable toxicity, treatment discontinuation requested by the subject, investigator's decision, and study termination by the sponsor.

7.7.2 Primary efficacy endpoint OS

The 10 subjects (02009, 14006, 24001, 28002, 38002, 40003, 43005, 64002, 07014, and 46005) with incorrect randomization stratification will not be included in the PPS for analysis.

Note: Subjects 07011 and 14007 with incorrect randomization stratification are not included in the PPS since the treatment duration is less than 6 weeks and the reason for treatment discontinuation is non-PD or non-death.

7.8 Other Analyses

No

8. STATISTICAL ANALYSIS SOFTWARE

All statistical analyses will be performed using SAS 9.4.

9. REFERENCES

- National Medical Products Administration, Provisions for Drug Registration, Jul. 2020
- National Medical Products Administration, Good Clinical Practice, Jul. 2020

➢ International Conference on Harmonisation of Technical Requirements for Registration of Pharmaceuticals for Human Use. E9 Statistical Principles for Clinical Trials.

➢ National Medical Products Administration, Guidances on Biological Statistics of Drug Clinical Trials, Jun. 2016

➢ National Medical Products Administration, Guidelines on Data Management and Plan and Report of Statistical Analysis of Drug Clinical Trials, Jul. 2016

10. LIST OF PROTOCOL DEVIATIONS

Class	Subclass	Description of Protocol Deviation	Severity of Protocol Deviation	Important Protocol Deviation
**1 Inclusion/ Exclusion Criteria**	Eligibility	The age of the subject does not meet the enrollment criteria	Major	No
	Eligibility	Not meet the criterion of pathologically confirmed pancreatic cancer derived from pancreatic ductal epithelium	Critical	Yes
	Eligibility	Not meet the criterion of unresectable locally advanced or metastatic pancreatic cancer	Critical	Yes
	Eligibility	Not meet the inclusion criterion 3	Critical	Yes
	Eligibility	Not meet the laboratory requirements in inclusion criterion 7	Critical	No
	Eligibility	The washout period of prior anti-tumor treatment does not meet the enrollment requirements or the AE of previous treatment does not recover to Grade ≤ 1 as per CTCAE 4.03 or stable state	Critical	No
	Eligibility	The subject has ascites requiring clinical intervention during the screening period, which meets the exclusion criteria	Critical	No
	Eligibility	The subject has a second malignancy, which meets the exclusion criteria	Critical	Yes
	Eligibility	With significant gastrointestinal disorders during the screening period, which meets the exclusion criteria	Major	No
	Eligibility	The subject has active hepatitis during the screening period, which meets the exclusion criteria	Critical	No
	Eligibility	Not meet a certain inclusion criterion and/or meet a certain exclusion criterion - other conditions than the above	Critical	Depends on the situation

Class	Subclass	Description of Protocol Deviation	Severity of Protocol Deviation	Important Protocol Deviation
**2 Informed Consent**	Informed consent criteria	The subject has been randomized or treated with the study drug before signing the informed consent form	Critical	Yes
	Informed consent criteria	The subject signs the informed consent form after starting any study-related procedures	Critical	Yes
	Informed consent criteria	An incorrect version of ICF is signed (not signing the latest version)	Critical	No
	Informed consent criteria	The contact information of the ethics committee or study site is not shown in the informed consent form	Major	No
	Informed consent criteria	The informed consent form is not dated	Major	No
	Informed consent criteria	The informed consent process is not documented	Major	No
	Informed consent criteria	Failure to follow the informed consent procedure	Critical	No
	Informed consent criteria	The investigator who signs the informed consent form is not in the authorization form or is not authorized	Major	No
**3 Visit Schedule**	Criteria for visit schedule	The subject does not complete some visit examinations	Depends on the situation	Depends on the situation
	Criteria for visit schedule	The routine follow-up visit is not performed	Major	No
	Criteria for visit schedule	The safety follow-up visit is not performed	Major	No
	Criteria for visit schedule	The withdrawal visit is not performed	Major	No
	Criteria for visit schedule	The tumor progression follow-up is not performed	Depends on the situation	No
	Criteria for visit schedule	Subject visit exceeds the time window	Depends on the situation	No
	Criteria for visit schedule	Prior to the occurrence of radiographically confirmed progressive disease, the imaging examination method and site of examination do not conform to the protocol requirements or the imaging examination method is inconsistent with that at baseline (except for difference in examination methods due to contrast media allergy during the treatment)	Depends on the situation	Depends on the situation
	Criteria for visit schedule	Subject is lost to follow-up	Depends on the situation	No

Class	Subclass	Description of Protocol Deviation	Severity of Protocol Deviation	Important Protocol Deviation
**4 Study Procedures**	Criteria for study procedures	A certain examination or an indicator in the examination exceeds the time window	Depends on the situation	No
	Criteria for study procedures	A certain examination or an indicator in the examination is missing	Depends on the situation	No
	Criteria for study procedures	A certain examination or an indicator in the examination is excessively performed	Depends on the situation	No
	Criteria for study procedures	The administration is not given within 48 h after enrollment	Depends on the situation	No
	Management criteria	The responsibility authorization form has not been updated before the start of the study procedures	Minor	No
	Criteria for study procedures	Authorization requirements are not followed	Major	No
	Criteria for ethical approval	Documents without ethical approval are dispensed to the subject	Critical	No
	The principal investigator's supervision of the entire study	Insufficient supervision, especially the lack of evidence of supervision by the principal investigator regarding the subject safety	Major	Yes
	Criteria for source documents	The study site changes the storage location of the source records of the subjects without notifying the CRA or providing access to the source records	Major	Yes
**5 Concomitant Medication**	Concomitant medication criteria	During the treatment, subject has used an anti-tumor traditional Chinese medicine approved by the NMPA before the radiographically confirmed disease progression	Critical	Yes
	Concomitant medication criteria	During the treatment, the subject has used a prohibited anti-tumor drug (except for the anti-tumor traditional Chinese medicine) before the radiographically confirmed disease progression	Critical	Yes
	Concomitant medication criteria	During the treatment, the subject has received an anti-tumor treatment prohibited by the study protocol (including the anti-tumor traditional Chinese medicine) after the radiographically confirmed disease progression	Critical	No
	Concomitant medication criteria	During the study, the subject has received non-anti-tumor treatment prohibited by the study protocol (e.g., immunomodulator)	Critical	No

Class	Subclass	Description of Protocol Deviation	Severity of Protocol Deviation	Important Protocol Deviation
**6 Subject Discontinuation**	Criteria for study procedures	The subject meets the criteria for study discontinuation, but does not discontinue the treatment (except for reason of intolerable toxicity)	Depends on the situation	No
	Criteria for study procedures	The subject does not meet the criteria for study discontinuation, but is withdrawn from the study per the investigator's judgment	Depends on the situation	Depends on the situation
	Criteria for study procedures	Interruption or discontinuation of study treatment that does not follow the requirements of the protocol	Depends on the situation	Depends on the situation
**7 Laboratory**	Biological samples of the study	The blood sample for gene testing has been collected but is unqualified	Minor	No
	Biological samples of the study	The blood sample for gene testing has been collected, but the volume is not sufficient	Minor	No
	Biological samples of the study	Study personnel for blood sampling are not authorized	Major	No
	Biological samples of the study	Not meet the biological sample storage/transport conditions	Critical	No
	Biological samples of the study	Blood samples are not processed as required by the protocol	Major	No
**8 Safety Monitoring**	SAE	The SAE is recorded with inappropriate method or reported at incorrect time points	Critical	No
	Review of safety report	The investigator does not review and sign the safety reports of subjects in time	Major	No
	AE/SAE	The follow-up and outcome recording of AE/SAE are not conducted as required by the protocol	Depends on the situation	No
	AE	Incorrect or inappropriate recording time of AEs	Depends on the situation	No
**9 Study Drug**	Time of administration	The administration time does not meet the requirements of the protocol	Major	No
	Out-of-window administration	Out-of-window administration (If out-of-window visit occurs first, there is no need to report the out-of-window administration)	Major	No

Class	Subclass	Description of Protocol Deviation	Severity of Protocol Deviation	Important Protocol Deviation
	Sequence of administration	The sequence of chemotherapy does not meet the protocol requirements	Major	No
	Dose	The dose of chemotherapy is higher than that specified in the protocol	Critical	Depends on the situation
	Dose	The dose of chemotherapy is lower than that specified in the protocol	Critical	Depends on the situation
	Compliance to study drugs	Missed dose of study drugs	Critical	Depends on the situation
	Criteria for study procedures	The study drugs are not kept blinded as required by the protocol	Critical	Yes
	Criteria for study procedures	Dose modification of study drugs does not follow the requirements of the protocol	Depends on the situation	Depends on the situation
	Drug overtemperature	The storage temperature of the drug exceeds the required temperature range but conforms to the storage instruction	Minor	No
	Drug overtemperature	The storage temperature of the drug exceeds the required temperature range and does not conform to the storage instruction without reported abnormality	Critical	No
	Temperature record	The drug administrator fails to record the highest temperature and lowest temperature as per the routine requirements	Critical	Yes
	Drug overtemperature	The storage temperature of the drug exceeds the required temperature range, and the study site has used the drug prohibited by the sponsor	Critical	Yes
	Drug preparation	The drug preparation process does not meet the protocol requirements	Critical	No
	Disposal of drugs	The study site or the subject discards the vial of used drug. Discrepancies in the inventory of the study drugs/not follow the authorization letter for destruction of the sub-site	Depends on the situation	No
	Randomization without study medication	The subject does not receive the study medication for some reasons after randomization	Major	No
	Randomization procedures	Some examinations should have been performed before randomization, but they are completed after randomization	Major	No

Class	Subclass	Description of Protocol Deviation	Severity of Protocol Deviation	Important Protocol Deviation
	Stratification error	Filling error of randomization stratification factors or randomization error due to that the stratification factor is not in line with the actual condition	Critical	Yes
	Compliance to study drugs	The subject is given the drug with other drug codes, especially irinotecan liposome	Major	Yes
**10 Subject Diary Card**	Criteria for study procedures	Less than 80% of the diary card is completed	Major	No
	Criteria for study procedures	The subject does not bring the diary card to the study site	Minor	No
	Criteria for study procedures	The investigator does not review and sign the subject diary card in time	Major	No
**11 Others**	Others	Other deviations not mentioned above	No	Depends on the situation

11. ATTACHED TABLES
